# Endophytes of Brazilian Medicinal Plants With Activity Against Phytopathogens

**DOI:** 10.3389/fmicb.2021.714750

**Published:** 2021-09-01

**Authors:** Jucélia Iantas, Daiani Cristina Savi, Renata da Silva Schibelbein, Sandriele Aparecida Noriler, Beatriz Marques Assad, Guilherme Dilarri, Henrique Ferreira, Jürgen Rohr, Jon S. Thorson, Khaled A. Shaaban, Chirlei Glienke

**Affiliations:** ^1^Postgraduate Program of Microbiology, Parasitology and Pathology, Department of Pathology, Federal University of Paraná, Curitiba, Brazil; ^2^Department of Biomedicine, Centro Universitário Católica de Santa Catarina, Joinville, Brazil; ^3^Postgraduate Program of Genetics, Federal University of Paraná, Curitiba, Brazil; ^4^Department of General and Applied Biology, Biosciences Institute, State University of São Paulo, Rio Claro, Brazil; ^5^Department of Pharmaceutical Sciences, College of Pharmacy, University of Kentucky, Lexington, KY, United States; ^6^Center for Pharmaceutical Research and Innovation, College of Pharmacy, University of Kentucky, Lexington, KY, United States

**Keywords:** phytopathogens, endophytes, secondary metabolites, natural product, endophytic fungi

## Abstract

Plant diseases caused by phytopathogens are responsible for significant crop losses worldwide. Resistance induction and biological control have been exploited in agriculture due to their enormous potential. In this study, we investigated the antimicrobial potential of endophytic fungi of leaves and petioles of medicinal plants *Vochysia divergens* and *Stryphnodendron adstringens* located in two regions of high diversity in Brazil, Pantanal, and Cerrado, respectively. We recovered 1,304 fungal isolates and based on the characteristics of the culture, were assigned to 159 phenotypes. One isolate was selected as representative of each phenotype and studied for antimicrobial activity against phytopathogens. Isolates with better biological activities were identified based on DNA sequences and phylogenetic analyzes. Among the 159 representative isolates, extracts from 12 endophytes that inhibited the mycelial growth (IG) of *Colletotrichum abscissum* (≥40%) were selected to expand the antimicrobial analysis. The minimum inhibitory concentrations (MIC) of the extracts were determined against citrus pathogens, *C. abscissum*, *Phyllosticta citricarpa* and *Xanthomonas citri* subsp. *citri* and the maize pathogen *Fusarium graminearum*. The highest activity against *C. abscissum* were from extracts of *Pseudofusicoccum stromaticum* CMRP4328 (IG: 83% and MIC: 40 μg/mL) and *Diaporthe vochysiae* CMRP4322 (IG: 75% and MIC: 1 μg/mL), both extracts also inhibited the development of post-bloom fruit drop symptoms in citrus flowers. The extracts were promising in inhibiting the mycelial growth of *P. citricarpa* and reducing the production of pycnidia in citrus leaves. Among the isolates that showed activity, the genus *Diaporthe* was the most common, including the new species *D. cerradensis* described in this study. In addition, high performance liquid chromatography, UV detection, and mass spectrometry and thin layer chromatography analyzes of extracts produced by endophytes that showed high activity, indicated *D. vochysiae* CMRP4322 and *P. stromaticum* CMRP4328 as promising strains that produce new bioactive natural products. We report here the capacity of endophytic fungi of medicinal plants to produce secondary metabolites with biological activities against phytopathogenic fungi and bacteria. The description of the new species *D. cerradensis*, reinforces the ability of medicinal plants found in Brazil to host a diverse group of fungi with biotechnological potential.

## Introduction

Plant diseases caused by microorganisms lead to significant crop losses worldwide (Oerke, 2006; Cantrell et al., 2012; Tan et al., 2018; Sang and Kim, 2020). It is estimated that the fungal diseases cause approximately 20% of yield reductions in commercial crops and major foods worldwide (Wang et al., 2016; Fisher et al., 2018; Zhao et al., 2020). In Brazil, economically important crops such as citrus and maize are affected by different phytopathogens that can cause losses in production or render products unviable for export (Ferrigo et al., 2016; Guarnaccia et al., 2017; Ference et al., 2018).

The production of citrus is an important agricultural activity, and in Brazil it occupies a relevant position in the production of fruit, both for the fresh fruit market and to produce concentrate orange juice (Carvalho et al., 2019; González-González et al., 2020). However, this important crop is affected by different pathogens, such as *Colletotrichum abscissum*, *Phyllosticta citricarpa, Xanthomonas citri* subsp. *citri* (Silva-junior et al., 2014; Guarnaccia et al., 2017; Behlau, 2021). The post-bloom fruit drop (PDF) is caused by *C. abscissum* and affects citrus crops at the blossom stage, causing petal lesions, and abscission of young fruits (De Menezes et al., 2014; Silva-junior et al., 2014). The pathogen *P. citricarpa* causes the citrus black spot (CBS) disease, which affects fruits and leaves of citrus, and cause early fruit drop (Glienke et al., 2011; Guarnaccia et al., 2019; Hendricks et al., 2020). Moreover, citrus canker, caused by *X. citri* subsp. *citri*, is a major bacterial disease affecting citrus production in Brazil (Ference et al., 2018).

Maize production stands out in Brazilian agriculture. The country is the third largest global producer and the second largest maize exporter (Allen and Valdes, 2016). However, several species within the *Fusarium graminearum* species complex are the cause of major diseases in maize crops including *F. graminearum*, an important phytopathogen in maize crops, that frequently infects cobs and stalks of maize (*Zea mays* L.) causing the disease fusarium head blight (FHB). This pathogen results in visibly damaged kernels that can lower the grade, and, therefore, the value of the harvested crop (Ferrigo et al., 2016; Walder et al., 2017; Pfordt et al., 2020).

The control or prevention of pathogens is mainly based on the application of synthetic agrochemicals and the use of cultural practices. Although agrochemicals used represent 50% of citrus production costs, control of citrus pathogens is often unsatisfactory (Agostini et al., 2006; Qin et al., 2016; Schreuder et al., 2018; Guarnaccia et al., 2019; Hendricks et al., 2020), and the presence of agrochemicals residues may limit the export of these products (Food and Drug Administration, 2012). The continuous use of agrochemicals also led to an increase in the occurrence and selection of resistant fungal pathogens (Rodrigues et al., 2007; Deising et al., 2008; Hendricks et al., 2013). In addition, synthetic agrochemicals also led to concerns about environmental contamination and human health (Dayan et al., 2009; Kim et al., 2018). In this context, the discovery of new and safer compounds with activity against citrus and maize pathogens is desired.

Potential sources of new secondary metabolites are endophytic fungi that can produce chemically diverse compounds with a broad range of bioactivities (Aly et al., 2011; Tan et al., 2018). Endophytes are microorganisms that reside within the internal tissues of living plants, without visibly harming the host (Quiroga et al., 2001; Hardoim et al., 2015). These microorganisms, when associated with medicinal plants, possess high potential to produce new bioactive metabolites (Savi et al., 2018, 2019a; Singh et al., 2021). Microorganisms have been regarded as a promising source with the most potential for structurally novel antifungal metabolites because of the isolation of many natural fungicides from the microbial resource (Copping and Duke, 2007; Pham et al., 2019).

Among the endophytic fungi, the genus *Diaporthe* has been isolated from different host plants, and is reported as great source of compounds, especially with antibacterial and antifungal activities (Gomes et al., 2013; Santos et al., 2016; Noriler et al., 2019). *Diaporthe* species are commonly isolated from medicinal plants, found in a range of biomes and recent studies have demonstrated antifungal activity of *Diaporthe* endophytes isolated from medicinal plants against citrus phytopathogens such as *C. abscissum* and *P. citricarpa* (Tonial et al., 2017; Noriler et al., 2018, 2019; Savi et al., 2020). However, only a small fraction of the approximately one million known endophytes have been investigated (Savi et al., 2019a; Manganyi and Ateba, 2020). In addition, several rare medicinal plants produce important bioactive compounds to survive in unique environments and may host new and diverse fungal endophytes (Strobel, 2003), which have rarely been isolated and characterized (Tan et al., 2018).

The Cerrado (savanna) and Pantanal (wetland) are recognized as Brazilian biomes with high diversity of species (Myers et al., 2000; Strassburg et al., 2017; Guerreiro et al., 2019). *Stryphnodendron adstringens* (Martius) Coville (Forero, 1972) is a native plant in the Brazilian savanna, recognized by medicinal properties and was added in the “Plants for the Future Initiative Midwest region,” an action of the Brazilian Ministry of the Environment (Souza-Moreira et al., 2018; Pellenz et al., 2019; de Souza et al., 2020). This species has been harbored a richness endophytic fungi community with biotechnological potential (Carvalho et al., 2012; Noriler et al., 2018). Additionally, *Vochysia divergens* Pohl (Vochysiaceae) is one of the main medicinal tree species in the Pantanal region (de Carvalho Filho et al., 2021). Endophytic fungi reported by *V. divergens* have been showed antimicrobial, antifungal and cytotoxicity activities (Hokama et al., 2016; Savi et al., 2018; Noriler et al., 2018, 2019). Considering the need for more effective treatments of plant diseases caused by citrus and maize pathogens, the present study identified fungal endophytes of two medicinal plants, *V. divergens* and *S. adstringens*, and investigated their antimicrobial potential against the citrus pathogens *C. abscissum*, *P. citricarpa*, and *X. citri* subsp. *citri*, as well as against the maize pathogen *F. graminearum*.

## Materials and Methods

### Biological Material

#### Pathogens Cultures

The pathogens *C. abscissum* (CMRP704 – Ca142), *P. citricarpa* (CMRP06) and *F. graminearum* (LGMF1703) are deposited in the CMRP Taxonline Microbiological Collections of Paraná Network, at the Federal University of Paraná, Brazil^1^ and *X. citri* subsp. *citri* (*X. citri*; isolate 306 – IBSBF 1594) deposited in the IBSBF (culture collection of the Biological Institute Culture Collection of Phytopathogenic Bacteria).

#### Plants

The leaves and petioles of *V. divergens* and *S. adstringens* used for the isolation of endophytes were collected in January 2018 from two Brazilian regions: the Pantanal that is a wetland characterized by periods of flood and drought (Ivory et al., 2019), and the Cerrado that is a savannah characterized by the natural presence of fire (Strassburg et al., 2017). The leaves and petioles of *V. divergens* were collected in the Miranda River of the Pantanal (19°36′35″S 56°58′35″W) and on the Red River in Corumbá, Mato Grosso do Sul, Brazil. *S. adstringens* leaves and petioles were collected in the Cerrado along the BR262 (20°18′10.8″S 56°15′44.3″W). Samples were collected from 20 plants of *S. adstringens* and 20 plants of *V. divergens*. For both species, five leaves and five petioles were collected of each individual plant. The plant tissues were stored at 4°C and the isolation of endophytes was performed within 72 h.

### Isolation of Endophytes

The leaves and petioles used for the isolation of endophytic fungi were processed using the protocol described by Petrini (1986), with modifications: the leaves and petioles were submerged in autoclaved water for 1 min, immersion in 70% ethanol (v/v) for 1 min, then for 3 min in sodium hypochlorite 3% (v/v), then for 1 min in 70% ethanol (v/v), and then, washed in sterilized distilled water for 1 min. After surface sterilization, the samples were cut into five pieces of 8 × 8 mm and aseptically transferred to Petri dishes containing potato dextrose agar (PDA) supplemented with nalidixic acid (50 μg/mL), at pH 5.8. The Petri dishes were incubated at 28°C for 30 days. The growth of endophytes was checked daily, and emerging mycelia were transferred to culture tubes containing PDA for further identification. To test the efficacy of surface disinfection method the last rinsed distilled water was collected and plated on the PDA plates and incubated at 28°C. The absence of fungal colonies growth from the last rinsed water plates indicates the efficacy of the surface sterilization procedure and confirming that the isolates were endophytes originated from within the host plant tissues.

After macroscopic characteristic analysis, the isolates were grouped into phenotypes according to colony color, growth rate, hyphal aspect, and presence/absence of spores. One isolate of each phenotype was randomly selected for molecular identification and bioprospecting. For each representative of the phenotypes, a pure culture was obtained from a single spore or hyphal tip according to Gilchrist-Saavedra et al. (2006).

### DNA Extraction, PCR Amplification, Sequencing, and Phylogenetic Analyses

Genomic DNA was extracted from 3-day old cultures using the method described by Raeder and Broda (1985). PCR amplification of partial regions of ITS (internal transcribed spacer) using corresponding primer pairs V9G/ITS4 (White et al., 1990; De Hoog and Gerrits van den Ende, 1998). For the *Diaporthe* genus were also used partial sequences of beta-tubulin (*tub2*), translation elongation factor 1-α (*tef1*), calmodulin (*cal*), and histone H3 (*his3*) genes using corresponding primer pairs, e.g., Bt2a/Bt2b (Glass and Donaldson, 1995), EF1-728F/EF1-986R (Carbone and Kohn, 1999), CAL- 228F/CAL-737R (Carbone and Kohn, 1999), and CYLH3F/H3-1b (Glass and Donaldson, 1995; Crous et al., 2004), respectively. PCR reaction was performed using Top Taq Master Mix (QIAGEN), purified with the Exo1 and FastAP enzymes (Thermo Fisher Scientific, United States) and sequenced using the BigDye Terminator Cycle Sequencing Kit v 3.1 Kit. The products were purified with Sephadex G50 and submitted to an ABI3500^®^ automated sequencer (Applied Biosystems, Foster City, CA, United States). Chromatograms were inspected using the MEGA 7 software (Kumar et al., 2015) and the consensus sequences were compared to those available in the NCBI/GenBank database (National Center for Biotechnology Information – http://www.ncbi.nlm.nih.gov/BLAST/) using the Blast tool, limiting to the sequences of the type strains.

The species found were also compared to the list of validated species of the MycoBank database^2^. The alignment was performed using the MAFFT version 7 (Katoh and Toh, 2008) and phylogeny based on Bayesian inference was performed in the MrBayes version 3.2.1 (Ronquist et al., 2011), using two parallel runs with one cold and three heated chains each, using the number of generations needed to reach split frequencies of ≤0.01 and a sampling frequency set to every 100 generations. The phylogenetic analyzes of *Diaporthe* genus were performed following the clades described by Gomes et al. (2013); Gao et al. (2017); Guarnaccia et al. (2018); Yang et al. (2018); Manawasinghe et al. (2019); Guo et al. (2020), and Hilário et al. (2021). In the present study we analyzed 173 *Diaporthe* isolates (Supplementary Table 1) (eight endophytic isolates from this study).

All the identified isolates were deposited in the CMRP Taxonline Microbiological Collections of Paraná Network (see text footnote 1) at the Federal University of Paraná (UFPR).

### Taxonomy

The isolate CMRP4331 was cultivated in different culture media: PDA, oatmeal agar (OA), and 2% malt extract agar (MEA) at 25°C in darkness and colony diameters were measured 14 days after inoculation (Crous et al., 2009). Colony colors were rated according to Rayner (1970). To induce sporulation, the isolates were cultured on 2% tap water agar supplemented with sterile pine needles (PNA). Cultures were incubated at 25°C with a 12/12 h fluorescent light/dark cycle. 50 alpha conidia and 36 beta conidia were measured to calculate the mean size and standard deviation (SD). Moreover, the morphological characteristics were examined captured using an Olympus microscope equipped with an SC30 camera. Descriptions were deposited in the MycoBank database^3^.

### Antifungal Activity

#### Production of Extracts

A total of 159 isolates representing each phenotype were used to prepare the extracts. The isolates were cultured for 7 days on PDA dishes, pH 5.8 at 28°C. Three mycelial disks (1.2 cm) of endophytes were inoculated into Erlenmeyer flasks (250 mL) containing 100 mL ME (Malt extract; Schulz et al., 1995) and cultured under agitation (180 rpm, 28°C) for 14 days. The mycelium was separated from the culture medium by filtration with Whatman n°4 filter paper. The fermentative liquid was mixed with 4% (w/v) XAD-16 resin and stirred overnight, followed by centrifugation. The resin was washed with distilled water (three times) and then extracted with MeOH (three times). The obtained methanol extract was dried using rotary evaporator at 40°C. The generated dry extracts of 159 isolates were diluted in methanol to a final concentration of 10 mg/mL (Savi et al., 2020).

#### Evaluation of Mycelial Growth Inhibition Against Pathogenic Fungi

The first screening of antifungal activity was evaluated against *C. abscissum*. 100 μL of the extract was added to a Petri dish with PDA medium, and a pathogen mycelial disk (8 mm) was subsequently placed in the center of the plate. The fungicide Carbendazim (Derosal^®^; 1.0 mg/mL) and methanol were used as positive and negative controls, respectively. The plates were incubated in BOD (Biochemical Oxygen Demand) at 24°C for 7 days. The inhibition of pathogen growth caused by each extract was evaluated by comparing the treatments with the controls (Savi et al., 2015, 2020; Hokama et al., 2016). The experiment was performed in triplicate and the data were submitted to Mann Whitney test in GraphPad Prims v. 6.01. The percentage of inhibition (Quiroga et al., 2001) was calculated with the following formula: Pi = (Cd – Td)/Cd, where: Pi = Percent inhibition; Cd = control growth diameter; Td = treatment growth diameter.

The extracts that showed the elite results in inhibiting the growth of the pathogen *C. abscissum* were selected for all other tests, including the evaluation of mycelial growth inhibition of *P. citricarpa* and *F. graminearum* that followed the same experimental design. The growth inhibition rates were analyzed 7 and 21 days after inoculation for *F. graminearum* and *P. citricarpa*, respectively. The experiments were performed in triplicate.

#### Determination of the Minimum Inhibitory Concentration for Pathogenic Fungi

The minimum inhibitory concentrations (MIC) analyzes were performed adapted from Tonial et al. (2017). MIC was determined in 96-well plates, with 100 μL malt extract medium in each well (*C. abscissum* and *P. citricarpa*). Potato dextrose medium was used for *F. graminearum*. 50 μL of the extract were pipetted into the adjacent well for serial dilutions, and 10 μL of conidial suspension of the pathogens *C. abscissum*, *P. citricarpa*, and *F. graminearum* (10^6^ conidia/mL) were added in each well. The experiments were performed in triplicate and the plates were incubated at 28°C for 14 days. The MIC was determined as the lowest concentration of the extract capable of completely inhibiting pathogen growth (Hörner et al., 2008).

#### Determination of the Minimum Inhibitory Concentration for Pathogenic Bacteria

To perform tests against *X. citri* subsp. *citri*, this pathogen was cultivated in NYG/NYG-agar medium (nitrogen-yeast-glycerol: 5 gL^–1^ of peptone, 3 gL^–1^ of yeast extract, 2% glycerol; solid medium bacterial agar was added to 15 gL^–1^) at 29°C (Zamuner et al., 2020). The inhibitory concentration values (IC) of the extracts for *X. citri* were determined using the Resazurin Microtiter Assay Plate (REMA) adapted by Silva et al. (2013). Stock solutions were prepared by dissolving the extracts in NYG medium containing 1% DMSO (v/v) to the final concentration of 100 μg/mL. Exposure of the cells was done in 96-multiwell plates, in which each of the extracts was serially diluted per column of the plate (example: A1-H1, A2-H2, etc.) using NYG medium as the diluent to the resulting concentration range varying from 100 to 0.78 μg/mL. Cells of *X. citri* were cultivated in NYG from the starting OD_600 nm_ of ∼0.1 until the OD_600 nm_ of ∼0.40 (which contains 10^7^ cells/mL). A fraction of this culture was added to each of the wells in order to give the final concentration of 10^5^ cells/well in a total volume of 100 μL per well. The negative control was NYG medium without any extract, whereas the positive control was kanamycin at 20 μg/mL. The control of vehicle was 1% DMSO (v/v) mixed in the NYG medium. The REMA plates were incubated for 16 h at 29 ± 1°C. Development was performed by adding 15 μL of 0.1 mg/mL resazurin (Sigma-Aldrich, Germany) solution into each well, followed by 2-h incubation at 29 ± 1°C. Cell respiration was monitored by the fluorescence of resorufin (the reduced form of resazurin in the presence of NADH) using the BioTek plate reader Synergy H1N1 set to the excitation and emission wavelengths of 530 and 590 nm, respectively. The percentages of cell growth inhibition per extract concentration were plotted using the software Microcal Origin 8.0, and polynomial regression was used to estimate IC values (concentrations of extracts able to inhibit 100% of the cells in culture; Morão et al., 2018). To determine if an extract has bactericidal or bacteriostatic effect, aliquots from REMA were transferred to NYG-agar plates before resazurin was added by using a 96-plate replicator (8 × 12 Sigma-Aldrich, Germany). Plates were incubated at 29°C for 48 h to verify the ability of the treated cells to resume growth. Three independent experiments were performed in triplicates each.

#### Inhibition of Post-Bloom Fruit Drop Development in Citrus Flowers

To evaluate the ability of 12 extracts into inhibit the PFD development, citrus flowers were collected at the UFPR, Curitiba, Paraná, Brazil. Flowers without PFD symptoms were added in Petri dishes containing water agar medium (15 g of agar per 1 L of deionized H_2_O). In a petal of each flower were added 5 μL of endophyte extracts and 5 μL of conidial suspension of *C. abscissum* (10^6^ conidia/mL). Methanol was used as negative control and fungicide Carbendazim (Derosal^®^; 1.0 mg/mL) was the positive control. The dishes were kept at 28°C for 48 h, and the qualitative analysis was performed. The presence of PFD symptoms in the flower was considered a negative activity against PFD, and the absence of symptoms was considered a positive activity of extract. The test was performed in triplicate (Savi et al., 2020).

#### Inhibition of *Phyllosticta citricarpa* Pycnidia Development in Citrus Leaves

To evaluate the potential of 12 extracts to inhibit the development of *P. citricarpa* pycnidia, autoclaved *Citrus sinensis* leaves were used (20 min; 120°C; 1 atm). Leaves disks (Ø 10 mm) with 10 μL of the extract were placed on Petri dishes with water agar medium. Four disks of 2 mm *P. citricarpa* mycelia were inoculated close to each leaf fragment. Methanol was used as negative control and fungicide Carbendazim (Derosal^®^; 1.0 mg/mL) was the positive control. The Petri dishes were maintained for 21 days at 28°C with 12 h photoperiod. The pycnidia of *P. citricarpa* formed above the leaves were counted under a stereoscopic microscope. This test was performed in triplicate (Santos et al., 2016).

### General Experimental Procedures

High performance liquid chromatography, UV detection, and mass spectrometry (HPLC-UV/MS) analyses were accomplished with an Agilent *Infinity Lab LC/MSD* mass spectrometer (*MS* Model G6125B; Agilent Technologies, Santa Clara, CA, United States) equipped with an Agilent 1260 Infinity II Series Quaternary LC system and a Phenomenex NX-C18 column (250 × 4.6 mm, 5 μm) [Method: solvent A: H_2_O/0.1% formic acid, solvent B: CH_3_CN; flow rate: 0.5 mL/min; 0–30 min, 5–100% B (linear gradient); 30–35 min, 100% B; 35–36 min, 100–5% B; 36–40 min, 5% B]. Thin layer chromatography (TLC) was carried out using Polygram SIL G/UV254 (Macherey-Nagel and Co., Dueren, Germany).

## Results

### Phenotypes and Screening of the Antifungal Activity

**FIGURE 1:**
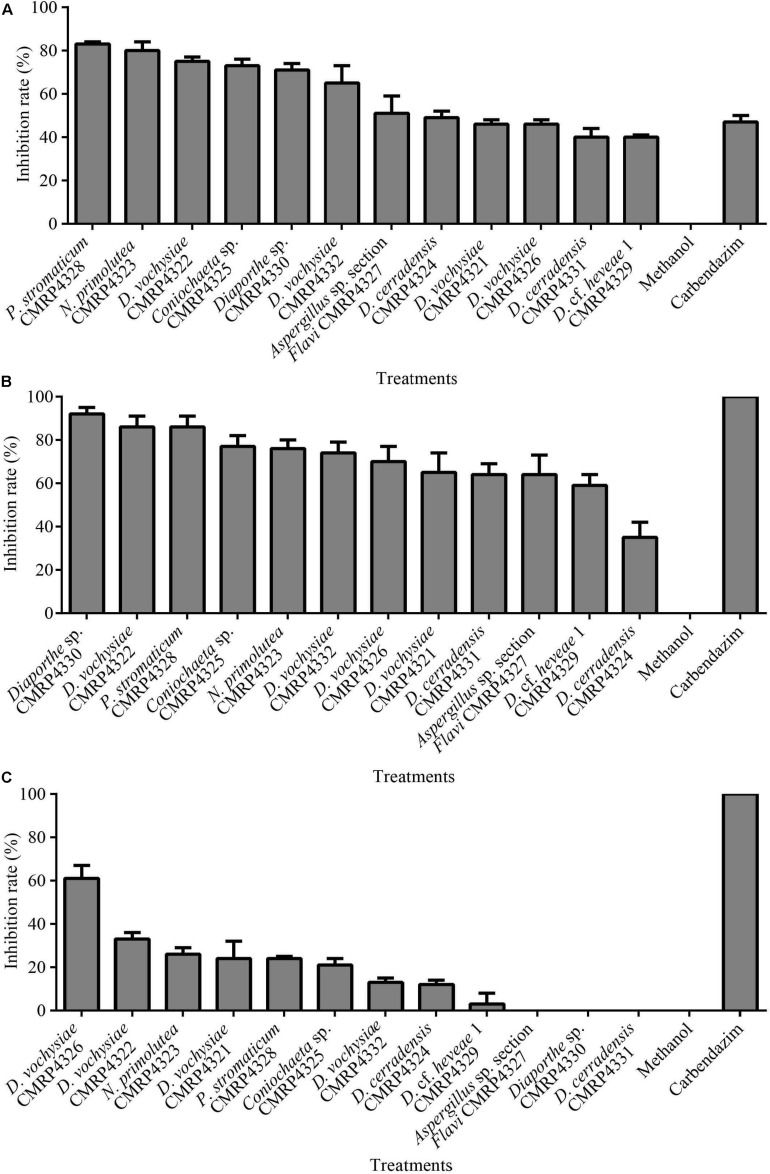
Mean values of mycelial growth inhibition (in%) of the pathogens *Colletotrichum abscissum* **(A)**, *Phyllosticta citricarpa* **(B)**, and *Fusarium graminearum* **(C)** in the presence of 100 μL of extracts obtained after culture of endophytes in malt extract medium. Note: Fungicide: 100 μL Carbendazim (Derosal; 0.1 mg/mL) was used as positive control.

A high number (1,304) of cultivable endophytic fungi were isolated from *V. divergens* and *S. adstringens*, and the isolates were grouped into 159 phenotypes based on morphological characteristics. The antifungal activity of the extracts produced from the fermentation of the 159 representative isolates (one of each phenotype) was determined (Supplementary Table 2). Twelve endophytic fungi produced metabolites that inhibited mycelial growth (IG) of the pathogen *C. abscissum* equal to or greater than 40%: CMRP4328 – IG: 83%, CMRP4323 – IG: 80%, CMRP4322 – IG: 75.3%, CMRP4325 – IG: 72.6%, CMRP4330 – IG: 71.3%, CMRP4332 – IG: 65.3%, CMRP4327 – IG: 51.3%, CMRP4324 – IG: 48.6%, CMRP4321 – IG: 46%, CMRP4326 – IG: 46%, CMRP4329 – IG: 40% and CMRP4331 – IG: 40% (Supplementary Table 2, Figure 1, and Supplementary Figures 1, 2).

Of the 12 isolates selected, seven were isolated from plants of *S. adstringens* (six from leaves and one from a petiole) located in the Cerrado biome, and five isolates were obtained from plants of *V. divergens* (two from leaves and three from petioles) of the Pantanal biome in Brazil (Supplementary Table 3).

### Identification of Isolates

**FIGURE 2:**
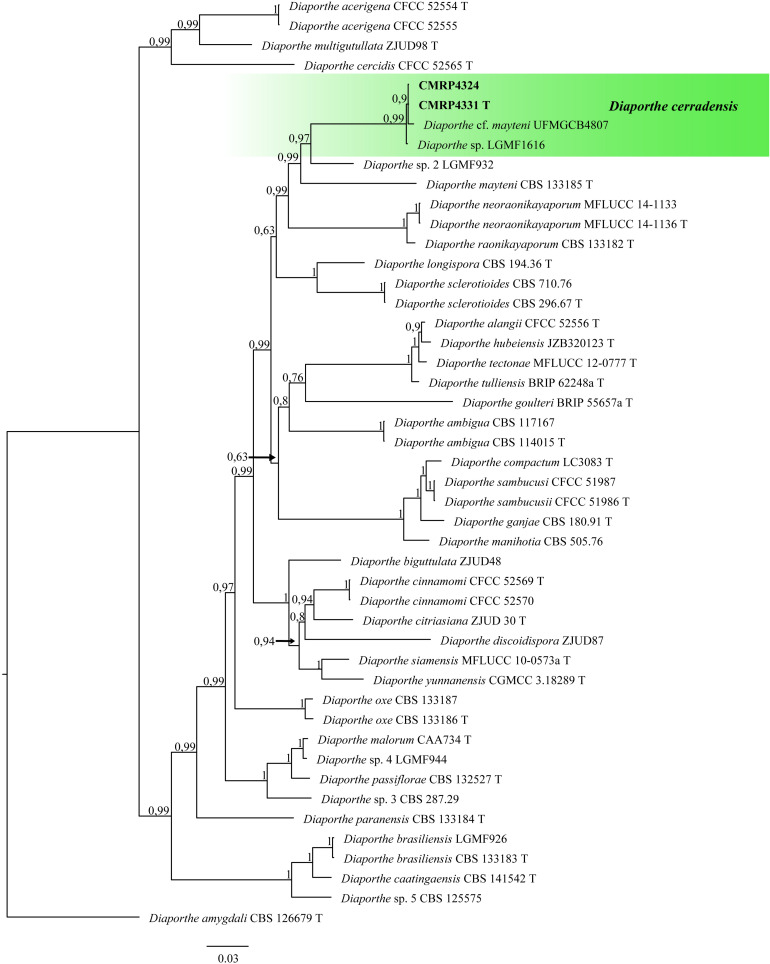
Bayesian Inference phylogenetic tree of *Diaporthe* species based on multiple alignment of ITS, *tub2*, *tef1*, *his3*, and *cal* partial sequences. The data matrix had 47 taxa and 2,133 characters. The species *Diaporthe amygdali* (CBS 126679 T) was used as outgroup. Strains marked with a “*T*” correspond to type sequences. The scale bar of 0.03 represents the number of changes. The sequence of the isolates here studied is presented with its isolation code (CMRP4331 and CMRP4324) highlighted in bold.

The 12 isolates were identified based on phylogenetic analysis using ITS (in addition to other loci) sequences as belonging to the Ascomycota phylum, with ten isolates belonging to the Sordariomycetes class, one to Eurotiomycetes and one to the Dothideomycetes class. *Diaporthe* was the genus with the highest number of isolates with antifungal potential, represented by eight isolates (Supplementary Table 3): the isolates CMRP4321, CMRP4322, CMRP4326, and CMRP4332 (*D. sojae* complex; Supplementary Figure 3), the CMRP4329 isolate (Supplementary Figure 4), the isolate CMRP4330 (*D. arecae* complex; Supplementary Figure 5) and CMRP4324 and CMRP4331 isolates (Figure 2). The other isolates were identified as *Aspergillus* section *Flavi A.* nomius-clade (CMRP4327; Supplementary Figure 6), *Coniochaeta* sp. (CMRP4325; Supplementary Figure 7), *Nemania primolutea* (CMRP4323; Supplementary Figure 8), and *Pseudofusicoccum stromaticum* (CMRP4328; Supplementary Figure 9 and Supplementary Table 4).

#### Phylogenetic Analyses

##### *Diaporthe* genus

###### *Diaporthe vochysiae* (CMRP4321, CMRP4322, CMRP4326, and CMRP4332)

In the *D. sojae* species complex, 60 representatives of the complex including the outgroup sequences of *Diaporthe amygdali* (CBS126679) were subjected to multi-locus phylogenetic analyses with concatenated ITS, *tub2*, *tef1*, *his3*, and *cal* sequences. A total of 2,439 characters including gaps (593 for ITS, 442 for *tub2*, 369 for *tef1*, 516 for *his3*, and 519 for *cal*) were used in the phylogenetic analysis, 647 characters were parsimony-informative, 921 were variable and 1453 characters were conserved.

In this clade, the isolates CMRP4321, CMRP4322, CMRP4326, and CMRP4332 were closely related to the type strain of *D. vochysiae* (Supplementary Figure 3), and therefore, we suggest them as belonging to this species.

###### *Diaporthe cf. heveae 1* (CMRP4329)

The multi-locus phylogenetic analyses with 29 representative isolates including the outgroup sequences of *D. perjuncta* (CBS 114435 T). A total of 2,160 characters including gaps (525 for ITS, 396 for *tub2*, 326 for *tef1*, 451 for *his3*, and 459 for *cal*) with 620 characters being parsimony-informative, 792 were variable, and 1,315 characters were conserved. The isolate CMRP4329 was assigned as *Diaporthe* cf. *heveae* 1 (Supplementary Figure 4).

###### *D. arecae* species complex (CMRP4330)

The isolate CMRP4330 was aligned with 49 representative isolates from *D. arecae* species complex including *D. caulivora* (CBS 127268 T) as outgroup. Were analyzed and 2,112 characters including gaps (517 for ITS, 340 for *tub2*, 323 for *tef1*, 457 for *his3*, and 475 for *cal*) were included in the multi-locus dataset, 474 characters were parsimony-informative, 721 were variable, and 1,361 characters were conserved. The CMRP4330 isolate was positioned on a single branch and, therefore, we suggest that this isolate belongs to a new species of the genus *Diaporthe*, named *Diaporthe* sp. (Supplementary Figure 5), since all attempts to produce are productive structure have failed.

###### *Diaporthe cerradensis* (CMRP4331 and CMRP4324)

The phylogenetic tree to *D. cerradensis* was obtained with 47 representative isolates including the outgroup sequences of *D. amygdali* (CBS 126679 T), a total of 2,133 characters including gaps (505 for ITS, 399 for *tub2*, 356 for *tef1*, 469 for *his3*, and 404 for *cal*). 761 characters were parsimony-informative, 926 were variable, and 1,157 characters were conserved. Based on these multi-locus phylogenic analyses, the isolates CMRP4324 and CMRP4331 were described as a new species and named *D. cerradensis* (Figure 2). In addition to these, the results for the first 10 megablast sequences using GenBank^4^ are in Supplementary Tables 5–9. The highest similarity was for the isolate *Diaporthe* cf. *mayteni* for the partial *tef1* (99.69%) and *tub* (99.59%). There are no *his* and *cal* sequences for *Diaporthe* cf. *mayteni*. Two endophytic strains were clustered in the same clade of *D. cerradensis*, the strain *Diaporthe* cf. *mayteni* UFMGCB4807 isolated from a leaf of *Carapa guianensis* (Ferreira et al., 2015) and *Diaporthe* sp. LGMF1616 isolated from a leaf of *S. adstringens* (Noriler et al., 2018), both also isolated in the Cerrado biome, Brazil. Below, we describe these new species.

Species Description

*Diaporthe cerradensis* CMRP4331

*Diaporthe cerradensis*: Iantas, Noriler & Glienke, *sp. nov*. MycoBank 839350;

*Etymology*: Named after the region where the strain was collected (Cerrado, Brazilian biome)

Pycnidial conidiomata globose, conical or irregular, solitary or aggregated, exposed on the PNA surface, dark brown to black, cream translucent conidial drops exuded from the ostioles, 170–350 μm diameter. *Conidiophores* hyaline, densely aggregated, unbranched, aseptate, subcylindrical to cylindrical, 4.5–6.5 × 2–4.3 μm. *Conidiogenous cells* hyaline and subcylindrical, tapering toward the apex 12.2–15.1 × 2–3.5 μm. *Alpha conidia* common, hyaline, fusiform, biguttulate, 6.4–8.3 × 2–3 μm, mean ± SD = 7.32 ± 0.38 × 2.31 ± 0.18 (*n* = 50). *Beta conidia* less common hyaline, aseptate, filiform, curved 18.8–29.6 × 1–1.6 μm, mean ± SD = 24.8 ± 2.7 × 1.4 ± 0.1 μm (*n* = 36). *Gamma conidia* not observed.

**FIGURE 3:**
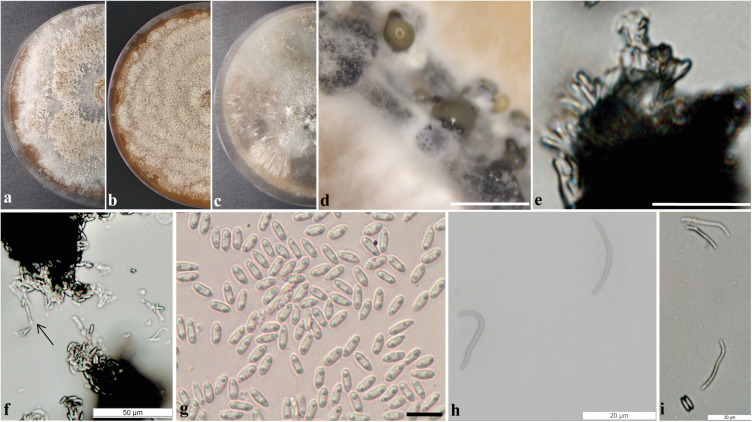
*Diaporthe cerradensis* (CMRP4331). **(a–c)** Colonies on PDA, MEA, and OA, respectively; **(d)** conidiomata sporulating on PNA; **(e)** conidiophores; **(f)** conidiogenous cells; **(g)** alpha conidia; **(h)** beta conidia; and **(i)** alpha and beta conidia. — Scale bars: ***d*** = 200 μm; ***e*** = 50 μm; and ***g*** = 10 μm.

Culture characteristics: Colonies on flat PDA, aerial mycelium forming concentric rings with cotton texture, white and light yellow on the surface, colonies reaching 72 mm in diameter after 2 weeks at 25°C; reverse yellow amber. In flat OA, with a white aerial mycelium, olive gray on the surface, colonies reaching 65 mm in diameter; reverse white, gray and greenish olivaceous. In the flat MEA, aerial white mycelium and amber patches on the surface, colonies reaching 52 mm diameter; reverse brown (Figure 3).

Specimen examined: Brazil, Miranda, Cerrado, Mato Grosso do Sul, 20°18′10.8″S, 56°15′44.3″W, endophytic species isolated from a leaf of *S. adstringens* (popular name Barbatimão), January 05, 2018 by C. Glienke and D. C. Savi. Holotype: UPCB97125 (Herbarium of the Department of Botany code, University Federal of Paraná), ex-type culture CMRP4331 CMRP – Taxonline Microbiological Collections of Paraná Network (see text footnote 1) at the UFPR.

Notes: *Diaporthe cerradensis* forms a well-supported and independent clade distinct from the previously described species of *Diaporthe*. *D. cerradensis* is most closely related to the species *D. mayteni*, *D. raonikayaporum*, and *D. neoraonikayaporum*. However, *D. cerradensis* differs from these species in the ITS sequences (36 different unique fixed alleles by *D. mayteni*, 30 by *D. raonikayaporum*, and 29 by *D. neoraonikayaporum*), *tef1* (27 different unique fixed alleles by *D. mayteni*, 45 by *D. raonikayaporum*, and 41 by *D. neoraonikayaporum*), *tub2* (19 different unique fixed alleles by *D. mayteni*, 26 by *D. raonikayaporum*, 23 by *D. neoraonikayaporum*), *his3* (30 different unique fixed alleles by *D. mayteni* and 40 by *D. raonikayaporum*), and *cal* (21 different unique fixed alleles by *D. mayteni*, 28 by *D. raonikayaporum*, 26 by *D. neoraonikayaporum*) loci. Furthermore, *D. cerradensis* differs from *D. mayteni* in having larger conidiogenous cells (12.2–15.1 × 2–3.5 vs 10–13 × 2–3 μm) and alpha conidia (6.4–8.3 × 2–3 vs 5–7 × 2–3 μm; Gomes et al., 2013). Conidiogenous cells of *D. cerradensis* are also larger than those of *D. raonikayaporum* (12.2–15.1 × 2–3.5 vs 5–10 × 2–3 μm; Gomes et al., 2013). In addition, alpha conidia of *D. cerradensis* are larger than those of *D. neoraonikayaporum* (6.4–8.3 × 2–3 vs 4–6 × 1.4–1.9 μm; Doilom et al., 2017).

##### *Aspergillus* sp. section *Flavi A.* nomius-clade (CMRP4327)

The isolate CMRP4327 was aligned with 54 sequences of 37 accepted species of the genus *Aspergillus* section *Flavi*, and *Aspergillus muricatus* (NRRL35674 T) was used as the outgroup. The alignment of the partial sequences of ITS consisted of 610 characters, 426 of these were conserved, 83 were parsimony informative, and 161 were uninformative. In the phylogenetic tree, the isolate CMRP4327 is closely related to the species *A. nomius* and *A. pseudonomius* (Supplementary Figure 6) and therefore, was denominated as belonging to the *A. nomius* clade.

##### *Coniochaeta* sp. (CMRP4325)

The phylogenetic analysis comprises the isolate CMRP4325, 35 species accepted in the *Coniochaeta* genus, and *Cheatosphaeria garethjonessi* MFLU161019 that was used as outgroup. The alignment of partial sequence of the ITS consisted of 612 characters, of which 331 were conserved, 171 were parsimony informative, and 261 uninformative. In the phylogenetic analysis, the isolate CMRP4325 is in the same branch as the *Coniochaeta boothii* species but seems to be a distinct form (Supplementary Figure 7). Multigene analysis will be necessary for the identification of this isolate.

##### *Nemania primolutea* (CMRP4323)

The alignment consisted of the isolate CMRP4323, 13 accepted species of the *Nemania* genus, and *Biscogniauxia nummularia* (MUCL51395) as the outgroup taxa. The ITS analysis comprises of 679 characters, which 322 were conserved, 175 were parsimony informative, and 290 were uninformative. Based on this phylogenetic analysis, the isolate CMRP4323 was identified as *N. primolutea* (Supplementary Figure 8).

##### *Pseudofusicoccum stromaticum* (CMRP4328)

The phylogenetic analysis was performed with the partial sequences of ITS and *tef1* of the isolate CMRP4328 and 15 sequences of 9 accepted species of the *Pseudofusicoccum* genus and using *Endomelanconiopsis microspora* (CBS353.97) as the outgroup taxa. The alignment of the partial sequence of ITS consisted of 575 characters, with 491 conserved sites, 16 were parsimony informative, and 74 uninformative. The partial *tef1* (Translation elongation factor 1 alpha) alignment consisted of 308 characters, with 211 conserved sites, 16 were parsimony informative, and 95 uninformative. In the phylogenetic tree the isolate CMRP4328 grouped with *P. stromaticum* and therefore, it was identified as belonging to this species (Supplementary Figure 9).

### Biological Activity

#### Extracts From Endophytic Fungi Inhibit the Mycelial Growth and Germination of Conidia of Plant Pathogens

In the screening for antifungal activity among the 159 extracts evaluated, 12 extracts showed highest mycelium growth inhibition against *C. abscissum* (Figure 1A) and were selected to be evaluated for their activity against the pathogens *P. citricarpa* and *F. graminearum*. Seven extracts inhibited more than 70% of the mycelial growth of the pathogen *P. citricarpa* (Figure 1B): *Diaporthe* sp. CMRP4330 (IG: 92%, *p* = 0.01), *P. stromaticum* CMRP4328 (IG: 86%, *p* = 0.01), *D. vochysiae* CMRP4322 (IG: 86%, *p* = 0.01), *Coniochaeta* sp. CMRP4325 (IG: 77%, *p* = 0.05), *N. primolutea* CMRP4323 (IG: 76%, *p* = 0.05), *D. vochysiae* CMRP4332 (IG: 74%, *p* = 0.05), and *D. vochysiae* CMRP4326 (IG: 70%, *p* = 0.05). Against the maize pathogen *F. graminearum* the extract from *D. vochysiae* CMRP4326 inhibited 61% of mycelial growth (*p* = 0.01; Figure 1C). *D. cerradensis* CMRP4324 and CMRP4331 inhibited the mycelial growth of citrus pathogens *C. abscissum* (IG = 49 and 40%, respectively) and *P. citricarpa* (IG = 35 and 64%, respectively).

For the 12 selected extracts, we also evaluated the minimum concentrations that inhibit the germination of the conidia of the pathogens. The values are showed in Table 1, and the underlined values represent extracts that also showed evidenced activity in the inhibition of mycelium growth (Figure 1 and Supplementary Figure 2). The extracts produced by the isolates *D. vochysiae* CMRP4322 completely inhibited the conidial germination of *C. abscissum* with the MIC value 1 μg/mL, and other isolates such as *N. primolutea* CMRP4323 (MIC: 40 μg/mL), *D. cerradensis* CMRP4331 (MIC: 40 μg/mL), *D. vochysiae* CMRP4326 (MIC: 40 μg/mL), and *P. stromaticum* CMRP4328 (MIC: 40 μg/mL) showed the lowest MIC values against *C. abscissum*.

**TABLE 1:** Minimum inhibitory concentration (μg/mL) of extracts produced by the endophytes against the pathogens *Colletotrichum abscissum, Phyllosticta citricarpa*, *Fusarium graminearum*, and *Xanthomonas citri* subsp. *citri* (in bold extracts with bactericidal activity), post-bloom fruit drop (PFD) symptoms in the presence of extracts and number of pycnidia produced in leaves by *P. citricarpa* in the presence of extracts.

		*C. abscissum*		*P. citricarpa*		*F. graminearum*	*X. citri*
Treatments		MIC μg/mL	PDF symptoms	MIC μg/mL	Pycnidia	MIC μg/mL	MIC μg/mL
*D. vochysiae*	CMRP4321	120	+	36	154 ± 7^a^	110	**85**
	CMRP4322	1	–	10	83 ± 6^*c^	40	100
	CMRP4326	40	+	40	138 ± 8^b^	10	101
	CMRP4332	36	+	36	33 ± 7^*e^	36	**99**
*D. cerradensis*	CMRP4331	40	+	10	91 ± 9^*c^	110	101
	CMRP4324	36	+	36	89 ± 16^*c^	110	100
*D.* cf. *heveae* 1	CMRP4329	36	+	36	155 ± 4^a^	110	106
*Diaporthe* sp.	CMRP4330	36	+	10	22 ± 5^*e^	36	100
*Coniochaeta* sp.	CMRP4325	36	+	40	148 ± 11^a^	36	101
*N. primolutea*	CMRP4323	40	–	40	52 ± 4^*d^	40	100
*P. stromaticum*	CMRP4328	40	–	10	42 ± 10^*e^	10	**79**
*Aspergillus* sp. section *Flavi*	CMRP4327	36	+	36	171 ± 12^a^	110	92
Carbendazim (control)		NE	–	NE	0^f^	NE	NE
Control with methanol		NE	+	NE	168 ± 14^a^	NE	NE

Underlined MIC – extracts with higher mycelial growth inhibition values, MIC against X. citri (in bold extracts with bactericidal activity), - absence of symptoms, + presence of symptoms, and NE not evaluated. *Samples that were significant in the reduction of growth compared to the control in the ANOVA (p < 0.01). The same subscript letter in the same column means the values are not different.

The lowest MIC values (10 μg/mL) that inhibit the germination of *P. citricarpa* conidia were produced by four isolates, including the new species *D. cerradensis* CMRP4331, and *D. vochysiae* CMRP4322, *Diaporthe* sp. CMRP4330, and *P. stromaticum* CMRP4328 (Table 1). The extracts of the isolates *D. vochysiae* CMRP4326 and *P. stromaticum* CMRP4328 inhibited the conidia germination of *F. graminearum* with MIC values of 10 μg/mL.

#### Extracts of Endophytic Fungi Have Activity Against Citrus Pathogenic Bacteria

Four extracts showed inhibitory activity against *X. citri* subsp. *citri* in which the IC values were below 100 μg/mL: *P. stromaticum* CMRP4328 (MIC: 79 μg/mL), *D. vochysiae* CMRP4321 (MIC: 85 μg/mL) *Aspergillus* sp. section *Flavi* (MIC: 92 μg/mL), and *D. vochysiae* CMRP4332 (MIC: 99 μg/mL). Extracts from *D. vochysiae* CMRP4321, *P. stromaticum* CMRP4328, and *D. vochysiae* CMRP4332 displayed bactericidal action (Table 1).

#### Inhibition of Post-Bloom Fruit Drop Development in Citrus Flowers

**FIGURE 4:**
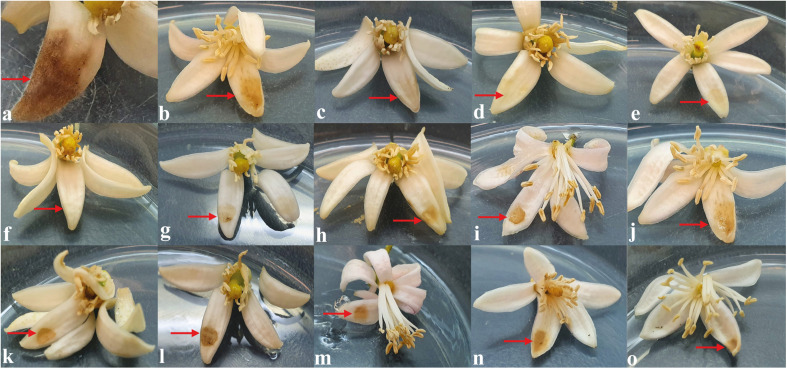
*In vitro* assay to evaluate the activity of the extracts to inhibit the development of post-bloom fruit drop (PFD) in citrus flowers, 48 h after inoculation with 5 μL of conidial suspension of *Colletotrichum abscissum* (10^6^ conidia/mL; red arrows) added with: **(a)** 5 μL of saline solution (showing the characteristic symptom of PDF), **(b)** 5 μL of methanol (control), **(c)** 10 μL with carbendazim (Derosal – control), and 5 μL of each endophytic extracts: **(d)** *Diaporthe vochysiae* CMRP4322, **(e)** *Nemania primolutea* CMRP4323, **(f)** *Pseudofusicoccum stromaticum* CMRP4328, **(g)** *D. vochysiae* CMRP4321, **(h)** *D. vochysiae* CMRP4326, **(i)** *D. vochysiae* CMRP4332, **(j)** *D. cerradensis* CMRP4331, **(k)** *D. cerradensis* CMRP4324, **(l)** *D.* cf. *heveae* 1 CMRP4329, **(m)** *Diaporthe* sp. CMRP4330, **(n)** *Coniochaeta* sp. CMRP4325, and **(o)** *Aspergillus* sp. section *Flavi* CMRP4327.

The ability of the 12 extracts, which showed great antifungal activity against *C. abscissum*, to inhibit the PFD symptoms in citrus flowers, was evaluated. This qualitative analysis assesses the presence or absence of symptoms of PFD (typical peach-orange necrotic lesions). After 48 h of inoculation, the extracts produced by *D. vochysiae* CMRP4322, *N. primolutea* CMRP4223, and *P. stromaticum* CMRP4328 completely inhibit symptoms in flowers (Supplementary Table 1 and Figure 4). The lesions observed in the flowers treated with the other extracts are similar to the typical PFD lesion shown in Figure 4a.

#### Inhibition of *P. citricarpa* Pycnidia Development in Citrus Leaves

**FIGURE 5:**
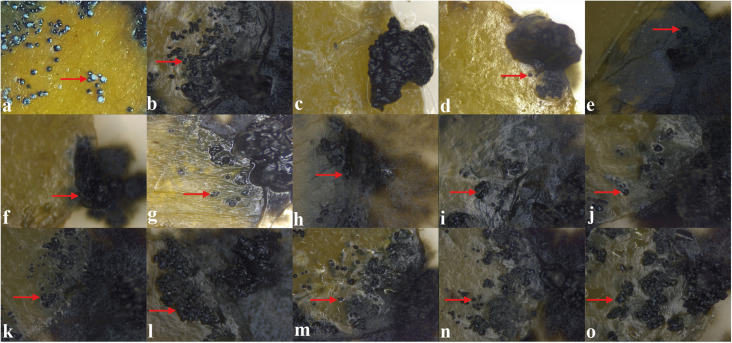
*In vitro* assay to evaluate the activity of the extracts to inhibit the development of pycnidia in citrus leaves, 21 days after the inoculation of mycelial disks of *Phyllosticta citricarpa* added with: **(a)** 10 μL of saline solution (showing characteristic pycnidia), **(b)** 10 μL of methanol (control), **(c)** 10 μL of carbendazim (Derosal; control), and 10 μL of each endophytic extracts: **(d)** *Diaporthe* sp. CMRP4330, **(e)** *D. vochysiae* CMRP4332, **(f)** *Pseudofusicoccum stromaticum* CMRP4328, **(g)** *Nemania primolutea* CMRP4323, **(h)** *D. vochysiae* CMRP4322, **(i)** *D. cerradensis* CMRP4324, **(j)** *D. cerradensis* CMRP4331, **(k)** *D. vochysiae* CMRP4326, **(l)** *Coniochaeta* sp. CMRP4325, **(m)** *D. vochysiae* CMRP4321, **(n)** *D.* cf. *heveae* 1 CMRP4329, and **(o)** *Aspergillus* sp. section *Flavi* CMRP4327. The red arrows represent the pycnidia formation.

We also evaluated the ability of extracts to inhibit the formation of *P. citricarpa* pycnidia in citrus leaves, and five extracts were able to inhibit more than 50% of pycnidia production (Supplementary Table 1 and Figure 5), *Diaporthe* sp. CMRP4330 (86%; Figure 5d), *D. vochysiae* CMRP4332 (81%; Figure 5e), *P. stromaticum* CMRP4328 (75%; Figure 5f), and *N. primolutea* CMRP4323 (69%; Figure 5g). We highlight the extracts from strains *Diaporthe* sp. CMRP4330 and *P. stromaticum* CMRP4328 that were able to reduce pycnidia formation and the mycelial growth.

### HPLC-UV/MS and TLC Analyses of Selected Fungal Extracts

**FIGURE 6:**
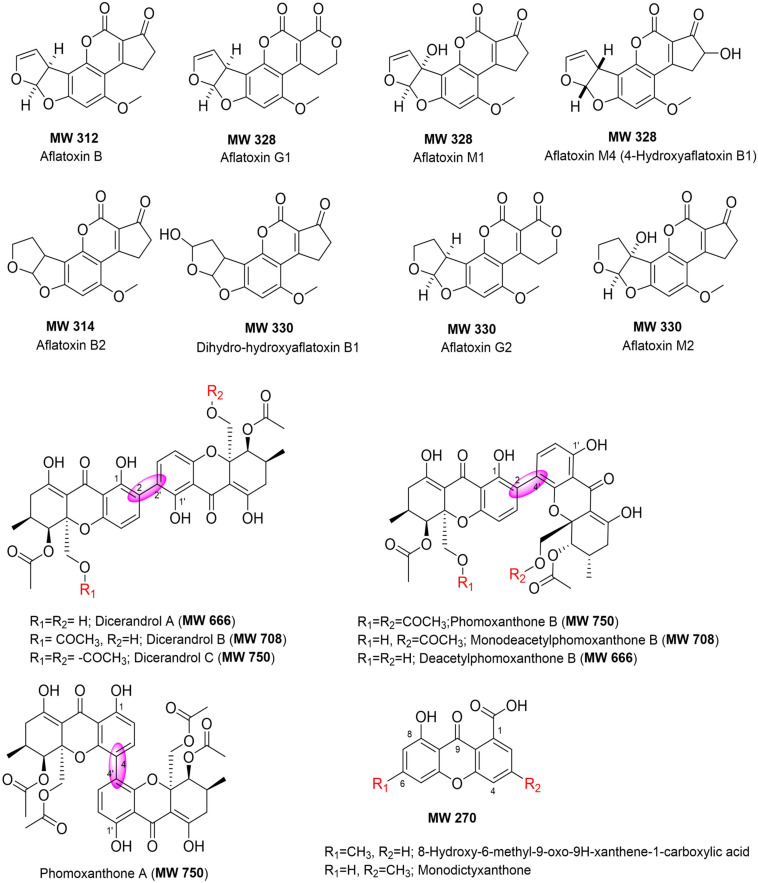
Chemical structures of compounds identified in the extracts of selected endophytic fungi (using HPLC-UV/vis, MS and TLC analyses, and AntiBase search).

The bioactive extracts produced by the top interesting 12 endophytic fungi (*D. vochysiae* CMRP4321, CMRP4322, CMRP4326, and CMRP4332, *D. cerradensis* CMRP4324 and CMRP431, *D.* cf. *heveae* 1 CMRP4329, *Diaporthe* sp. CMRP4330, *N. primolutea* CMRP4323, *Coniochaeta* sp. CMRP4325, *Aspergillus* sp. section *Flavi* CMRP4327, and *P. stromaticum* CMRP4328) have been subjected for chemical screening (TLC and HPLC-UV/MS analyses) followed by metabolite data analysis. The extracts generated from the small-scale fermentations of these fungal strains have been dissolved in MeOH and subjected to HPLC-UV/MS and TLC analyses. The major class of compounds are aflatoxins and xanthones (Figure 6). The HPLC-UV/MS analyses of these extracts displayed several interesting UV/vis and MS peaks (Supplementary Figures 10–42). For example, HPLC-UV/MS analyses of the extracts produced by four strains (*D. vochysiae* CMRP4321, CMRP4322, and CMRP4332, and *P. stromaticum* CMRP4328) indicate the presence of several interesting compounds with similar UV/vis chromophores (Supplementary Figures 10–16, 30, 31, 38–41). In the same manner, the LCMS analysis of the fungus *Coniochaeta* sp. CMRP4325 extract showed two major peaks with molecular weights of 324 and 306 Daltons, respectively, with Δ*m/z* = 18 Daltons difference, consistent with loss of water. AntiBase (Laatsch, 2017) search afforded no matched hits, which likely indicates two new fungal metabolites (Supplementary Figures 22, 23). The HPLC-UV/MS analysis of another fungus extract (*Aspergillus* sp. section *Flavi* CMRP4327) was very rich in terms of its metabolites (Supplementary Figures 26–29) and shows several peaks with similar UV/vis that matched with the aflatoxin class of compounds (Figure 6). The LC mass spectra of these peaks revealed three peaks with different retention times, but the same molecular weight (MW 338) as well as other major peaks with MW of 354, 312, and 422 Daltons. In addition, the TLC of the *Aspergillus* sp. section *Flavi* CMRP4327 extract (Supplementary Figure 42) displayed several blue-fluorescent UV bands at 254/365 nm. The extract of *D.* cf. *heveae* 1 CMRP4329 showed three major HPLC peaks with similar UV/vis chromophores, with a major peak of MS = 324 Daltons. No mass match for these compounds were found in an AntiBase search, indicating new compounds in this crude extract. The fungus strain *Diaporthe* sp. CMRP4330 displayed two major peaks (HPLC *R*_t_ = 20.96 and 21.19 min) with a similar UV/vis spectrum and the same molecular weight (MW 338; Supplementary Figure 34). An AntiBase search showed no hits matching the molecular weights and UV/vis of these two HPLC peaks, which indicates novelty of these two compounds. The generated extracts by the remaining fungal strains (*N. primolutea* CMRP4323, *D. cerradensis* CMRP4324 and CMRP4331, and *D. vochysiae* CMRP4326) did not show major peaks in the HPLC-MS analyses, however, they might contain some none-UV/vis bioactive compounds, or the extracts produced from these strains did not contain compound with good solubility in MeOH.

## Discussion

Plant diseases caused by microorganisms remain one of the major obstacles in agriculture, and more effective treatments for diseases caused by pathogens of citrus and maize are necessary. An ecologically attractive alternative to control plant diseases is the use of secondary metabolites produced by endophytes (Khare et al., 2018; Savi et al., 2019a). In the past decade we isolated a large number of endophytic fungi from leaves and petioles of the medicinal plants *V. divergens* and *S. adstringens*, respectively, located in two highly diverse biomes in Brazil: Pantanal and Cerrado (Carvalho et al., 2012; Hokama et al., 2016; Noriler et al., 2018). Here, we report on 12 endophytes producing secondary metabolites that were able to inhibit the mycelial growth of important pathogens of citrus (*C. abscissum*, *P. citricarpa*, and *X. citri*). We also report on extracts that inhibited the maize pathogenic fungus *F. graminearum*.

The isolates with biological activity (Supplementary Table 3) belong to the Ascomycota phylum, one of the most prevalent eukaryotic groups in the world (Park et al., 2017). In addition, Sordariomycetes was the most prevalent class among the endophytes selected. *Diaporthe* was the dominant genus among the endophytes that showed potential for production of compounds with antifungal activity. The genus *Diaporthe* belongs to the family Diaporthaceae and is composed of hundreds of species (Chepkirui and Stadler, 2017). We performed multilocus DNA sequence analyses using five loci (ITS, *tef1*, *tub2*, *his3*, and *cal*) commonly used in previous phylogenetic studies of *Diaporthe* species (Gomes et al., 2013; Guarnaccia et al., 2018; Yang et al., 2018; Guo et al., 2020). The final phylogenetic trees distinguished four species of the *Diaporthe* genus that showed potential against plant pathogens, including the new species *D. cerradensis* described in this study. This species was collected in the Cerrado, the Brazilian savanna and the extracts produced by two representatives of this species (CMRP4324 and CMRP4331) showed interesting results, especially against the pathogenic citrus fungi *C. abscissum* and *P. citricarpa*.

The genus *Diaporthe* is a rich source of bioactive secondary metabolites, including volatile and non-volatile compounds, which have been shown to be effective against plant pathogens (Singh et al., 2011; Noriler et al., 2019; Savi et al., 2020; Xu et al., 2021). This genus has also been recognized as a producer of several new compounds of great interest in biotechnology (Nicoletti and Fiorentino, 2015; Santos et al., 2016; Tahir et al., 2017). Santos et al. (2016) reported that endophytic isolates of *D. terenbinthifolii* were able to colonize citrus plants and produce secondary metabolites with activity against *P. citricarpa*, suggesting that this species could be used as a potential biological controller of the CBS disease. The multilocus phylogenetic analyses revealed four isolates belonging to the *D. vochysiae* species. This species was described by Noriler et al. (2019), in which compounds produced by the isolate LGMF1583 were characterized and tested, showing compounds with activity against human pathogenic bacteria.

Fungi endophytes isolates from two Brazilian biomes, Cerrado and Pantanal showed potential to produce secondary metabolites with activity against phytopathogens. In the present study, we isolated the endophyte *D. vochysiae* from both biomes and four strains showed potential to inhibit the growth of both target phytopathogens of this study. This species was initially isolated from the Pantanal biome as an endophyte of *V. divergens* (Noriler et al., 2019) and the extract produced from this isolate showed activities against human pathogens. The four strains belonging to the new species *D. cerradensis* were isolated from plants of the Cerrado biome, two obtained in this study, one (LGMF1616) obtained by Noriler et al. (2018) also from *S. adstringens* leaves and one isolate (UFMGCB4807, previously called *Diaporthe* cf. *mayteni*) was obtained as an endophyte from plant leaves of *C. guianensis* by Ferreira et al. (2015).

Both plant tissues (leaf and petiole) harbored endophytes producers of secondary metabolites with biological activity. One example is the species *D.* cf. *heveae* 1 isolated in the present study as an endophyte of petiole of *S. adstringens*, and isolated from leaf of *S. adstringens* by Noriler et al. (2018). The secondary metabolites from both strains showed activity against plant pathogens (Noriler et al., 2018; Savi et al., 2020). Our data reinforces the importance of both Brazilian biomes as a source of endophytes with biotechnological potential. Further studies can provide more information about fungi endophytic community structures across tissues and biomes.

Extracts produced by the isolates *D. vochysiae* CMRP4322, *P. stromaticum* CMRP4328, and *N. primolutea* CMRP4323 also inhibited the mycelial growth of *C. abscissum* more than the fungicide carbendazim, demonstrating the potential of the metabolites from these endophytic isolates to control citrus pathogens. These same extracts inhibited the development of PFD symptoms in the flowers (Figures 1A, 4). These results are promising since the treatment with the fungicide carbendazim can reduce, but not eliminate the phytopathogen development in field (Savi et al., 2020). In addition to the inhibition of mycelial growth, the isolate *D. vochysiae* CMRP4322 also inhibited the germination of *C. abscissum* conidia at lower than 1 μg/mL concentrations. These results are quite interesting due to the PFD cycle caused by the production of conidia in already infected flowers, which are then splashed on surrounding leaves, twigs and floral buds that remain after the blossom period (Agostini et al., 1992; Silva-junior et al., 2014; Pinho et al., 2015; Silva A. O. et al., 2016; Savi et al., 2019b). In Brazil, PDF disease leads to large losses in citrus production, estimated 80%, when rainy periods occur during the bloom phase (Goes et al., 2008; Silva A. O. et al., 2016).

Isolates *D. vochysiae* CMRP4322, *Diaporthe* sp. CMRP4330, and *P. stromaticum* CMRP4328 produced extracts that significantly inhibited the mycelial growth of *P. citricarpa*. We also showed that these extracts considerably reduced the formation of pycnidia on leaf surfaces (Figures 1B, 5). *P. citricarpa* affects fruit, leaves and twigs of several citrus hosts causing diverse symptoms (Kotzé, 2000; Guarnaccia et al., 2019). To control the CBS several fungicide applications may be necessary, leading to an increase in the costs of citrus production. However, if this disease is not fully controlled, it depreciates the commercial value of the fruits (Silva G. J. et al., 2016; Schreuder et al., 2018; Hendricks et al., 2020). In addition, citrus fruits with CBS symptoms are subject to quarantine regulations in the European Union restricting market access for countries with the presence of CBS disease and reduce the availability of citrus fruits to consumers in the off-season in Europe (Agostini et al., 2006; EU, 2015; Moyo et al., 2020).

Four isolates, *D. vochysiae* CMRP4322, *Diaporthe* sp. CMRP4330, *D. cerradensis* CMRP4331, and *P. stromaticum* CMRP4328 showed low values of MIC (10 μg/mL) capable of inhibiting the germination of *P. citricarpa* conidia. According to Sposito et al. (2011) and Tran et al. (2018), conidia are important sources of inoculum of *P. citricarpa* and play an important role in increasing the prevalence and spread of CBS disease. Therefore, these four extracts may be explored as an alternative option for CBS control. Endophytic microorganisms or their secondary metabolites can be used as alternatives to reduce the application of fungicides in the control of CBS and PFD (Santos et al., 2016; Tonial et al., 2017; Savi et al., 2020).

Three extracts analyzed (*D. vochysiae* CMRP4321 and CMRP4332, as well as *P. stromaticum* CMRP4328) were bactericidal against *X. citri* subsp. *citri*. This pathogenic bacterium causes citrus canker, a serious disease that affects citrus production worldwide (Fu et al., 2020). *X. citri* can colonize all above ground tissues of citrus plants, including young leaves, thorns, shoots, and fruits (Ferreira et al., 2016; Ference et al., 2018). As one of the most important citrus pathogens, *X. citri* causes serious economic losses due to reduced crop yield and quality, the costs of eradication measures, containment and obstacles to fruit exports. Moreover, the control of this bacterium results in an important environmental alert related to increased use of copper-based pesticides and the development of resistance to antimicrobials (Miller et al., 2009; Robène et al., 2020; Behlau, 2021).

The isolate *D. vochysiae* CMRP4326 inhibited the mycelial growth of a maize pathogen *F. graminearum.* Moreover, two extracts of isolates *D. vochysiae* CMRP4326 and *P. stromaticum* CMRP4328 completely inhibited the germination of conidia of this pathogen at low concentration (10 μg/mL). *F. graminearum* is an important pathogen affecting the yield and production of cereals, including maize and wheat worldwide (Schöneberg et al., 2016). In addition to causing direct yield loss through tissue rotting, *F. graminearum* can also cause crop contamination with mycotoxins, rendering the grain unsafe for human and livestock consumption and imposing higher costs on producers (Arunachalam and Doohan, 2013; Maresca, 2013; Pfordt et al., 2020). Different strategies are used to reduce the impact of FBH, including crop rotation, tillage practices, and fungicide application and planting of less susceptible cultivars (Jones et al., 2018). These strategies reached only low to moderate success (Wegulo et al., 2011; Mousa et al., 2015). Therefore, our results point to a promising alternative since secondary metabolites reported here prevent the germination of asexual spores, which is essential to break the cycle of the diseases.

HPLC-UV/MS analyses of the extracts produced by four strains (*D. vochysiae* CMRP4321, CMRP4322, and CMRP4332, and *P. stromaticum* CMRP4328) indicate the presence of several interesting compounds with similar UV/vis chromophores. AntiBase (Laatsch, 2017) search using MS and UV/vis, resulted in several xanthone-analogs (Figure 6) that matches with MS and UV/vis, including dicerandrol A (MW 666), dicerandrol B (MW 708), dicerandrol C (MW 750); phomoxanthone B (MW 750), or its mono- or di-deacetylated analogs (MW 708 and 666, respectively), all of them have been reported previously as fungal metabolites (Isaka et al., 2001; Wagenaar and Clardy, 2001; Zhang et al., 2008; Cao et al., 2012; Choi et al., 2013; Ding et al., 2013; Ronsberg et al., 2013; Kumla et al., 2017). The HPLC-UV/MS analysis of another fungus extract (*Aspergillus* sp. section *Flavi* CMRP4327) was very rich in metabolites (Supplementary Figures 26–29). AntiBase (Laatsch, 2017) and literature search using the aforementioned data indicates that this strain is a potential producer of aflatoxins (Asao et al., 1965; Benkerroum, 2020; Zhang and Banerjee, 2020). However, no aflatoxin analogs have been reported previously with MW 338, which designates this strain a potential producer of new aflatoxin-analogs (Laatsch, 2017).

Based on the aforementioned LCMS and TLC results, this work highlights that some of the selected strains including *D. vochysiae* CMRP4322, *D.* cf. *heveae* 1 CMRP4329, and *Diaporthe* sp. CMRP4330, *Coniochaeta* sp. CMRP4325, *Aspergillus* sp. section *Flavi* CMRP4327, and *P. stromaticum* CMRP4328, can now be considered promising strains for potential discovery of new bioactive natural products, and have been selected for future studies (including scale-up fermentations, isolation, and structure elucidation of the new bioactive compounds produced by these strains). Results will be published later somewhere else.

The compounds produced by endophytic microorganisms have been showing promise as a more environmentally friendly alternative (Wang et al., 2019). We reported here the capacity of 12 endophytic fungi isolated from tissues of the medicinal plants *V. divergens* and *S. adstringens* to produce secondary metabolites with biological activities against phytopathogenic fungi and bacteria. These metabolites directly affect the mycelium growth, germination of conidia, or symptoms development of important fungi pathogens of citrus (*C. abscissum* and *P. citricarpa*), and act also inhibiting the mycelial growth of the maize pathogen *F. graminearum*. In addition, some of these metabolites revealed bactericidal activity against an important pathogenic bacterium of citrus, *X. citri* subsp. *citri.* The herein described *D. cerradensis* is a new species of the *Diaporthe* genus. Its discovery again shows the capacity of medicinal plants found in Brazil to host a diverse group of fungi with biotechnological potential. Further studies are needed to identify the bioactive compounds and also for the field evaluation of the endophytes or their metabolites in the control of plant pathogens.

## Data Availability Statement

The datasets presented in this study can be found in online repositories. The names of the repository/repositories and accession number(s) can be found below: [Diaporthe cerradensis MB#839350 – (https://www.mycobank.org/)].

## Author Contributions

CG, DS, JI, and KS: conceived and designed the experiments. JI, RS, SN, BA, GD, and HF: performed the experiments. JI, DS, KS, and CG: analyzed the data. JI, DS, CG, KS, GD, and HF: writing – original draft. JI, DS, CH, JR, KS, and JT: writing – review and editing. CG, JR, KS, and JT: supervision. All authors read and approved the final manuscript.

## Conflict of Interest

The authors declare that the research was conducted in the absence of any commercial or financial relationships that could be construed as a potential conflict of interest.

## Publisher’s Note

All claims expressed in this article are solely those of the authors and do not necessarily represent those of their affiliated organizations, or those of the publisher, the editors and the reviewers. Any product that may be evaluated in this article, or claim that may be made by its manufacturer, is not guaranteed or endorsed by the publisher.

## Footnotes

1
www.cmrp-taxonline.com

2
http://www.mycobank.org/

3
www.MycoBank.org

4
https://blast.ncbi.nlm.nih.gov
