# Supplementary material for: Endophytes of Brazilian Medicinal Plants With Activity Against Phytopathogens
Source: Front Microbiol. 2021 Sep 1;12:714750. doi: 10.3389/fmicb.2021.714750 (PMC8442585; doi:10.3389/fmicb.2021.714750)
Supplement: Supplementary file 1 [file Data_Sheet_1.docx]

**Supplementary material**

**Endophytes of Brazilian Medicinal Plants with Activity Against Phytopathogens**

Jucélia Iantas^1^, Daiani Cristina Savi^2,3^, Renata da Silva Schibelbein^1^, Sandriele Aparecida Noriler^1^, Beatriz Marques Assad^3^, Guilherme Dilarri^4^, Henrique Ferreira^4^, Jürgen Rohr^5^, Jon S. Thorson^5,6^, Khaled A. Shaaban^5,6,^*, Chirlei Glienke^1,3^*

^1^Postgraduate Program of Microbiology, Parasitology and Pathology, Department of Pathology, Federal University of Paraná (UFPR), Curitiba, Paraná State, Brazil

^2^Department of Biomedicine, Centro Universitário Católica de Santa Catarina, Joinville, SC, Brazil.

#### ^3^Postgraduate Program of Genetics, Federal University of Paraná (UFPR), Curitiba, Paraná State, Brazil.

^4^Department of General and Applied Biology, Biosciences Institute, State University of São Paulo, UNESP, Rio Claro, SP, Brazil.

^5^Department of Pharmaceutical Sciences, College of Pharmacy, University of Kentucky, Lexington, Kentucky, United States.

^6^Center for Pharmaceutical Research and Innovation, College of Pharmacy, University of Kentucky, Lexington, Kentucky, United States.

*Correspondence: [ch.glienke@gmail.com](mailto:ch.glienke@gmail.com), [khaled_shaaban@uky.edu](mailto:khaled_shaaban@uky.edu)

| **Contents** | **Page** |
| --- | --- |
| Table S1. Collection details and GenBank accession numbers of isolates of *Diaporthe* genus included in this study. | 7 |
| Table S2. Screening of antifungal activity evaluated against the pathogen *Colletotrichum abscissum*. The fungicide Carbendazim (1.0 mg/mL) was used as positive control and methanol as negative control. | 15 |
| Table S3. Endophytic fungi used in this study isolated from the medicinal plants *Stryphnodendron adstringens* (*S.a*) and *Vochysia divergens* (*V.d*) from leaves or petioles. | 19 |
| Figure S1. Front (a) and back view (b), respectively of colonies on PDA of twelve endophytic fungi used in this study isolated from the medicinal plants *Stryphnodendron adstringens* and *Vochysia divergens*.. | 19 |
| Figure S2. Antifungal activity of extracts of the isolates representing the twelve phenotypes against the mycelial growth of the pathogens *Colletotrichum abscissum* (A), *Phyllosticta citricarpa* (B) and *Fusarium graminearum* (C). | 20 |
| Table S4. Collection details and GenBank accession numbers of isolates of *Aspergillus* section *Flavi*, *Coniocheata*, *Nemania* and *Pseudofusicoccum* genus included in this study. | 21 |
| Table S5. GenBank megablast result for the first 10 sequences using ITS (internal transcribed spacer) partial sequence from CMRP4331 isolate. | 26 |
| Table S6. GenBank megablast result for the first 10 sequences using *tub* (beta-tubulin) partial sequence from CMRP4331 isolate. | 26 |
| Table S7. GenBank megablast result for the first 10 sequences using *tef1* (translation elongation factor 1-alpha) partial sequence from CMRP4331 isolate. | 26 |
| Table S8. GenBank megablast result for the first 10 sequences using *his (*histone H3) partial sequence from CMRP4331 isolate. | 27 |
| Table S9. GenBank megablast result for the first 10 sequences using *cal* (calmodulin) partial sequence from CMRP4331 isolate. | 27 |
| Figure S3. Bayesian Inference phylogenetic tree of *Diaporthe* species based on multiple alignment ofITS, *tub2*, *tef1*, *his3* and *cal* partial sequences. The data matrix had 60 taxa and 2439 characters. The species *Diaporthe amygdali* (CBS126679) was used as outgroup. Strains marked with a “T” correspond to type sequences. The scale bar of 0.02 represents the number of changes. The sequence of the isolates here studied is presented with its isolation code (CMRP4321, CMRP4322, CMRP4326 and CMRP 4332) highlighted in bold. | 28 |
| Figure S4. Bayesian Inference phylogenetic tree of *Diaporthe* species based on multiple alignment of ITS, *tef1*, *tub2*, *his3 and cal* partial sequences. The data matrix had 29 taxa and 2160 characters. The species *Diaporthe perjuncta* (CBS 114435 T) was used as outgroup. Strains marked with a “T” correspond to type sequences. The scale bar of 0.02 represents the number of changes. The sequence of the isolate here studied is presented with its isolation code (CMRP4329) highlighted in bold. | 29 |
| Figure S5. Bayesian Inference phylogenetic tree of *Diaporthe* species based on multiple alignment of ITS, *tef1*, *tub2*, *his3 and cal* partial sequences. The data matrix had 49 taxa and 2112 characters. The species *Diaporthe caulivora* (CBS 127268 T) was used as outgroup. Strains marked with a “T” correspond to type sequences. The scale bar of 0.03 represents the number of changes. The sequence of the isolate here studied is presented with its isolation code (CMRP4330) highlighted in bold. | 30 |
| Figure S6. Bayesian Inference phylogenetic tree of *Aspergillus* section *Flavi* species based on alignment of ITS partial sequences. The data matrix had 55 taxa and *Aspergillus muricatus* (NRRL 35674) was used as outgroup. Strains marked with a “T” correspond to type sequences. The scale bar of 0.02 represents the number of changes. The sequence of the isolate here studied is presented with its isolation code (CMRP4327) highlighted in bold. | 31 |
| Figure S7. Bayesian Inference phylogenetic tree of *Coniocheata* genus based on alignment of ITS partial sequences. The data matrix had 45 taxa and *Cheatosphaeria garethjonessi (*MFLU161019) was used as outgroup. Strains marked with a “T” correspond to type sequences. The scale bar of 0.05 represents the number of changes. The sequence of the isolate here studied is presented with its isolation code (CMRP4325) highlighted in bold. | 32 |
| Figure S8. Bayesian Inference phylogenetic tree of *Nemania* genus based on alignment of ITS partial sequences. The data matrix had 16 taxa and *Biscogniauxia nummularia* (MUCL51395) was used as outgroup. Strains marked with a “T” correspond to type sequences. The scale bar of 0.06 represents the number of changes. The sequence of the isolate here studied is presented with its isolation code (CMRP4323) highlighted in bold. | 33 |
| Figure S9. Bayesian Inference phylogenetic tree of *Pseudofusicoccum* genus based on alignment of ITS partial sequences. The data matrix had 16 taxa and *Endomelanconiopsis microspora* (CBS353.97) was used as outgroup. Strains marked with a “T” correspond to type sequences. The scale bar of 0.02 represents the number of changes. The sequence of the isolate here studied is presented with its isolation code (CMRP4328) highlighted in bold. | 34 |
| Figure S10. HPLC/UV analyses of the extract produced by *Diaporthe vochysiae* CMRP4321. HPLC-conditions: solvent A: H_2_O/0.1% FA; solvent B: CH_3_CN; flow rate: 0.5 mL min^-1^; 0-30 min, 5-100% B (linear gradient); 30-35 min, 100% B; 35-36 min, 100-5% B (linear gradient); 36-40 min, 5% B; 254 nm. UV-vis inset of full wavelength scan (190-600 nm). | 35 |
| Figure S11. (+)-ESI-MS spectra of the compounds detected in the extract produced by *Diaporthe vochysiae* CMRP4321. LCMS-conditions: solvent A: H_2_O/0.1% FA; solvent B: CH_3_CN; flow rate: 0.5 mL min^-1^; 0-30 min, 5-100% B (linear gradient); 30-35 min, 100% B; 35-36 min, 100-5% B (linear gradient); 36-40 min, 5% B. Note – no clear mass peaks were detected in the (–)-ESI-MS. | 36 |
| Figure S12. HPLC/UV analyses of the extract produced by *Diaporthe vochysiae* CMRP4322. HPLC-conditions: solvent A: H_2_O/0.1% FA; solvent B: CH_3_CN; flow rate: 0.5 mL min^-1^; 0-30 min, 5-100% B (linear gradient); 30-35 min, 100% B; 35-36 min, 100-5% B (linear gradient); 36-40 min, 5% B; 254 nm, 280 nm, 320 nm, 400 nm. UV-vis inset of full wavelength scan (190-600 nm). | 37 |
| Figure S13. HPLC/UV analyses of the extract produced by *Diaporthe vochysiae* CMRP4322. HPLC-conditions: solvent A: H_2_O/0.1% FA; solvent B: CH_3_CN; flow rate: 0.5 mL min^-1^; 0-30 min, 5-100% B (linear gradient); 30-35 min, 100% B; 35-36 min, 100-5% B (linear gradient); 36-40 min, 5% B; 254 nm. UV-vis inset of full wave length scan (190-600 nm). | 38 |
| Figure S14. Summary of LCMS analyses of the compounds detected in the extract produced by *Diaporthe vochysiae* CMRP4322. LCMS-conditions: solvent A: H_2_O/0.1% FA; solvent B: CH_3_CN; flow rate: 0.5 mL min^-1^; 0-30 min, 5-100% B (linear gradient); 30-35 min, 100% B; 35-36 min, 100-5% B (linear gradient); 36-40 min, 5% B; 320 nm, 254 nm. | 39 |
| Figure S15. (+) and (–)-ESI-MS spectra of the compounds detected in the extract produced by *Diaporthe vochysiae* CMRP4322. LCMS-conditions: solvent A: H_2_O/0.1% FA; solvent B: CH_3_CN; flow rate: 0.5 mL min^-1^; 0-30 min, 5-100% B (linear gradient); 30-35 min, 100% B; 35-36 min, 100-5% B (linear gradient); 36-40 min, 5% B. | 40 |
| Figure S16. (+) and (–)-ESI-MS spectra of the compounds detected in the extract produced by *Diaporthe vochysiae* CMRP4322. LCMS-conditions: solvent A: H_2_O/0.1% FA; solvent B: CH_3_CN; flow rate: 0.5 mL min^-1^; 0-30 min, 5-100% B (linear gradient); 30-35 min, 100% B; 35-36 min, 100-5% B (linear gradient); 36-40 min, 5% B. | 41 |
| Figure S17. HPLC/UV analyses of the extract produced by *Nemania primolutea* CMRP4323. HPLC-conditions: solvent A: H_2_O/0.1% FA; solvent B: CH_3_CN; flow rate: 0.5 mL min^-1^; 0-30 min, 5-100% B (linear gradient); 30-35 min, 100% B; 35-36 min, 100-5% B (linear gradient); 36-40 min, 5% B; 210 nm, 254 nm. UV-vis inset of full wavelength scan (190-600 nm). | 42 |
| Figure S18. (+) and (–)-ESI-MS spectra of the major compounds detected in theextract produced by *Nemania primolutea* CMRP4323. LCMS-conditions: solvent A: H_2_O/0.1% FA; solvent B: CH_3_CN; flow rate: 0.5 mL min^-1^; 0-30 min, 5-100% B (linear gradient); 30-35 min, 100% B; 35-36 min, 100-5% B (linear gradient); 36-40 min, 5% B.Note – no clear mass peaks were detected in the (–)-ESI-MS. | 43 |
| Figure S19. HPLC/UV analyses of the extract produced by *Diaporthe cerradensis* CMRP4324. HPLC-conditions: solvent A: H_2_O/0.1% FA; solvent B: CH_3_CN; flow rate: 0.5 mL min^-1^; 0-30 min, 5-100% B (linear gradient); 30-35 min, 100% B; 35-36 min, 100-5% B (linear gradient); 36-40 min, 5% B; 254 nm. UV-vis inset of full wavelength scan (190-600 nm). | 44 |
| Figure S20. (+) and (–)-ESI-MS spectra of the compounds detected in the extract produced by *Diaporthe cerradensis* CMRP4324. LCMS conditions: solvent A: H_2_O/0.1% FA; solvent B: CH_3_CN; flow rate: 0.5 mL min^-1^; 0-30 min, 5-100% B (linear gradient); 30-35 min, 100% B; 35-36 min, 100-5% B (linear gradient); 36-40 min, 5% B. | 45 |
| Figure S21. (+) and (–)-ESI-MS spectra of the compounds detected in the extract produced by *Diaporthe cerradensis* CMRP4324. LCMS conditions: solvent A: H_2_O/0.1% FA; solvent B: CH_3_CN; flow rate: 0.5 mL min^-1^; 0-30 min, 5-100% B (linear gradient); 30-35 min, 100% B; 35-36 min, 100-5% B (linear gradient); 36-40 min, 5% B | 46 |
| Figure S22. HPLC/UV analyses of the extract produced by *Coniochaeta* sp. CMRP4325. HPLC-conditions: solvent A: H_2_O/0.1% FA; solvent B: CH_3_CN; flow rate: 0.5 mL min^-1^; 0-30 min, 5-100% B (linear gradient); 30-35 min, 100% B; 35-36 min, 100-5% B (linear gradient); 36-40 min, 5% B; 254 nm. UV-vis inset of full wavelength scan (190-600 nm). | 47 |
| Figure S23. (+) and (–)-ESI-MS spectra of the major compounds detected in the extract produced by *Coniochaeta* sp. CMRP4325. LCMS conditions: solvent A: H_2_O/0.1% FA; solvent B: CH_3_CN; flow rate: 0.5 mL min^-1^; 0-30 min, 5-100% B (linear gradient); 30-35 min, 100% B; 35-36 min, 100-5% B (linear gradient); 36-40 min, 5% B. | 48 |
| Figure S24. HPLC/UV analyses of the extract produced by *Diaporthe vochysiae* CMRP4326. HPLC-conditions: solvent A: H_2_O/0.1% FA; solvent B: CH_3_CN; flow rate: 0.5 mL min^-1^; 0-30 min, 5-100% B (linear gradient); 30-35 min, 100% B; 35-36 min, 100-5% B (linear gradient); 36-40 min, 5% B; 254 nm. UV-vis inset of full wavelength scan (190-600 nm). | 49 |
| Figure S25. (+) and (–)-ESI-MS spectra of the major compounds detected in the extract produced by *Diaporthe vochysiae* CMRP4326. LCMS conditions: solvent A: H_2_O/0.1% FA; solvent B: CH_3_CN; flow rate: 0.5 mL min^-1^; 0-30 min, 5-100% B (linear gradient); 30-35 min, 100% B; 35-36 min, 100-5% B (linear gradient); 36-40 min, 5% B. Note – no clear mass was detected for the major peak at *R*_t_=37.52 min | 50 |
| Figure S26. HPLC/UV analyses of the extract produced by *Aspergillus* sp. section *Flavi* CMRP4327. HPLC-conditions: solvent A: H_2_O/0.1% FA; solvent B: CH_3_CN; flow rate: 0.5 mL min^-1^; 0-30 min, 5-100% B (linear gradient); 30-35 min, 100% B; 35-36 min, 100-5% B (linear gradient); 36-40 min, 5% B; 254 nm. UV-vis inset of full wavelength scan (190-600 nm). | 51 |
| Figure S27. Summary of LCMS analyses of the compounds detected in the extract produced by *Aspergillus* sp. section *Flavi* CMRP4327. LC-MS conditions: H_2_O/0.1% formic acid (solvent A), CH_3_CN/0.1% formic acid (solvent B); flow rate: 0.5 mL min^-1^; 0-2 min, 5% B; 2-30 min, 5-100% B; 30-35 min, 100% B; 35-36 min, 100-5% B; 36-40 min, 5% B. | 52 |
| Figure S28. (+) and (–)-ESI-MS spectra of the compounds detected in the extract produced by *Aspergillus* sp. section *Flavi* CMRP4327. LCMS conditions: solvent A: H_2_O/0.1% FA; solvent B: CH_3_CN; flow rate: 0.5 mL min^-1^; 0-30 min, 5-100% B (linear gradient); 30-35 min, 100% B; 35-36 min, 100-5% B (linear gradient); 36-40 min, 5% B. | 53 |
| Figure S29. (+) and (–)-ESI-MS spectra of the compounds detected in the extract produced by *Aspergillus* sp. section *Flavi* CMRP4327. LCMS conditions: solvent A: H_2_O/0.1% FA; solvent B: CH_3_CN; flow rate: 0.5 mL min^-1^; 0-30 min, 5-100% B (linear gradient); 30-35 min, 100% B; 35-36 min, 100-5% B (linear gradient); 36-40 min, 5% B. | 54 |
| Figure S30. HPLC/UV analyses of the extract produced by *Pseudofusicoccum stromaticum* CMRP4328. HPLC-conditions: solvent A: H_2_O/0.1% FA; solvent B: CH_3_CN; flow rate: 0.5 mL min^-1^; 0-30 min, 5-100% B (linear gradient); 30-35 min, 100% B; 35-36 min, 100-5% B (linear gradient); 36-40 min, 5% B; 210 nm and 254 nm. UV-vis inset of full wavelength scan (190-600 nm). | 55 |
| Figure S31. (+) and (–)-ESI-MS spectra of the compounds detected in the extract produced by *Pseudofusicoccum stromaticum* CMRP4328. LCMS conditions: solvent A: H_2_O/0.1% FA; solvent B: CH_3_CN; flow rate: 0.5 mL min^-1^; 0-30 min, 5-100% B (linear gradient); 30-35 min, 100% B; 35-36 min, 100-5% B (linear gradient); 36-40 min, 5% B. Note – no clear mass was detected for the major peaks at *R*_t_=30.5 – 34.2 min. | 56 |
| Figure S32. HPLC/UV analyses of the extract produced by *Diaporthe* cf. *heveae* 1 CMRP4329. HPLC-conditions: solvent A: H_2_O/0.1% FA; solvent B: CH_3_CN; flow rate: 0.5 mL min^-1^; 0-30 min, 5-100% B (linear gradient); 30-35 min, 100% B; 35-36 min, 100-5% B (linear gradient); 36-40 min, 5% B; 254 nm. UV-vis inset of full wavelength scan (190-600 nm). | 57 |
| Figure S33. (+) and (–)-ESI-MS spectra of the compounds detected in the extract produced by *Diaporthe* cf. *heveae* 1 CMRP4329. LC-MS conditions: H_2_O/0.1% formic acid (solvent A), CH_3_CN/0.1% formic acid (solvent B); flow rate: 0.5 mL min^-1^; 0-2 min, 5% B; 2-30 min, 5-100% B; 30-35 min, 100% B; 35-36 min, 100%-5% B; 36-40 min, 5% B. Note – no clear mass was detected for the peaks at *R*_t_=31.89 and 32.57 min. | 58 |
| Figure S34. HPLC/UV analyses of the extract produced by *Diaporthe* sp. CMRP4330. HPLC-conditions: solvent A: H_2_O/0.1% FA; solvent B: CH_3_CN; flow rate: 0.5 mL min^-1^; 0-30 min, 5-100% B (linear gradient); 30-35 min, 100% B; 35-36 min, 100%-5% B (linear gradient); 36-40 min, 5% B; 254 nm, 210 nm, 280 nm. UV-vis inset of full wavelength scan (190-600 nm). | 59 |
| Figure S35. (+) and (–)-ESI-MS spectra of the compounds detected in the extract produced by *Diaporthe* sp. CMRP4330. LC-MS conditions: H_2_O/0.1% formic acid (solvent A), CH_3_CN/0.1% formic acid (solvent B); flow rate: 0.5 mL min^-1^; 0-2 min, 5% B; 2-30 min, 5-100% B; 30-35 min, 100% B; 35-36 min, 100%-5% B; 36-40 min, 5% B. | 60 |
| Figure S36. HPLC/UV analyses of the extract produced by *Diaporthe cerradensis* CMRP4331. HPLC-conditions: solvent A: H_2_O/0.1% FA; solvent B: CH_3_CN; flow rate: 0.5 mL min^-1^; 0-30 min, 5-100% B (linear gradient); 30-35 min, 100% B; 35-36 min, 100%-5% B (linear gradient); 36-40 min, 5% B; 254 nm, 280 nm. No major peaks were detected in the HPLC-UV analysis of this fungal extract. | 61 |
| Figure S37. (+)-ESI-MS spectra of the compounds detected in tthe extract produced by *Diaporthe cerradensis* CMRP4331. LC-MS conditions: H_2_O/0.1% formic acid (solvent A), CH_3_CN/0.1% formic acid (solvent B); flow rate: 0.5 mL min^-1^; 0-2 min, 5% B; 2-30 min, 5-100% B; 30-35 min, 100% B; 35-36 min, 100%-5% B; 36-40 min, 5% B.Note – no clear mass peaks were detected in the (–)-ESI-MS. | 62 |
| Figure S38. HPLC/UV analyses of the extract produced by *Diaporthe vochysiae* CMRP4332. HPLC-conditions: solvent A: H_2_O/0.1% FA; solvent B: CH_3_CN; flow rate: 0.5 mL min^-1^; 0-30 min, 5-100% B (linear gradient); 30-35 min, 100% B; 35-36 min, 100%-5% B (linear gradient); 36-40 min, 5% B; 320 nm, 254 nm, 210 nm, 400 nm, 280 nm. UV-vis inset of full wavelength scan (190-600 nm). | 63 |
| Figure S39. HPLC/UVand summary of LCMS analyses of the compounds detected in the extract produced by *Diaporthe vochysiae* CMRP4332. HPLC-conditions: solvent A: H_2_O/0.1% FA; solvent B: CH_3_CN; flow rate: 0.5 mL min^-1^; 0-30 min, 5-100% B (linear gradient); 30-35 min, 100% B; 35-36 min, 100%-5% B (linear gradient); 36-40 min, 5% B; 254 nm. UV-vis inset of full wavelength scan (190-600 nm). | 64 |
| Figure S40. (+) and (–)-ESI-MS spectra of the compounds detected in the extract produced by *Diaporthe vochysiae* CMRP4332. LC-MS conditions: H_2_O/0.1% formic acid (solvent A), CH_3_CN/0.1% formic acid (solvent B); flow rate: 0.5 mL min^-1^; 0-2 min, 5% B; 2-30 min, 5-100% B; 30-35 min, 100% B; 35-36 min, 100%-5% B; 36-40 min, 5% B. | 65 |
| Figure S41. (+) and (–)-ESI-MS spectra of the compounds detected in the extract produced by *Diaporthe vochysiae* CMRP4332. LC-MS conditions: H_2_O/0.1% formic acid (solvent A), CH_3_CN/0.1% formic acid (solvent B); flow rate: 0.5 mL min^-1^; 0-2 min, 5% B; 2-30 min, 5-100% B; 30-35 min, 100% B; 35-36 min, 100%-5% B; 36-40 min, 5% B. | 66 |
| Figure S42. TLC (CH_2_Cl_2_/10%MeOH) screening of the extracts produced by 12 selected endophytic fungi (*Diaporthe vochysiae* CMRP4321 (63), *Diaporthe vochysiae* CMRP4322 (67), *Nemania primolutea* CMRP4323 (68), *Diaporthe cerradensis* CMRP4324 (72), *Coniochaeta* sp. CMRP4325 (126), *Diaporthe vochysiae* CMRP4326 (128), *Aspergillus* sp. section *Flavi* CMRP4327 (129), *Pseudofusicoccum stromaticum* CMRP4328 (133), *Diaporthe* cf. *heveae* 1 CMRP4329 (140), *Diaporthe* sp. CMRP4330 (145), *Diaporthe cerradensis* CMRP4331 (152), *Diaporthe vochysiae* CMRP4332 (158)). | 67 |

Table 1. Collection details and GenBank accession numbers of isolates of *Diaporthe* genus included in this study.

|  |  |  |  | **GenBank accession no.^2^** | | | | |
| --- | --- | --- | --- | --- | --- | --- | --- | --- |
| **Species** | **Collection number^1^** | **Host** | **Country** | **ITS** | *tub2* | *tef1* | *his3* | *cal* |
| *Diaporthe acaciarum* | CBS138862* | *Acacia tortilis* | Tanzania | KP004460 | KP004509 | - | KP004504 | - |
| *D. acaciigena* | CBS 129521* | *Acacia retinodes* | Australia | KC343005 | KC343973 | KC343731 | KC343489 | KC343247 |
| *D. acerigena* | CFCC 52554* | *Acer tataricum* | China | MH121489 | - | MH121531 | MH121449 | MH121413 |
|  | CFCC 52555 | *A. tataricum* | China | MH121490 | - | MH121532 | MH121450 | MH121414 |
| *D. acuta* | PSCG 047* | *Pyrus pyrifolia* | China | MK626957 | MK691225 | MK654802 | MK726161 | MK691125 |
| *D. alangii* | CFCC 52556* | *Alangium kurzii* | China | MH121491 | MH121573 | MH121533 | MH121451 | MH121415 |
| *D. ambigua* | CBS 114015* | *Pyrus communis* | South Africa | KC343010 | KC343978 | KC343736 | KC343494 | KC343252 |
|  | CBS 117167 | *Aspalathus linearis* | South Africa | KC343011 | KC343979 | KC343737 | KC343495 | KC343253 |
| *D. ampelina* | CBS 114016* | *Vitis vinifera* | France | AF230751 | JX275452 | AY745056 | - | JX197443 |
|  | CPC 28254 | *V. vinifera* | UK | MG280970 | MG281143 | MG281491 | MG281316 | MG281664 |
| *D. amygdali* | CBS 126679* | *Prunus dulcis* | Portugal | KC343022 | KC343990 | KC343748 | KC343506 | KC343264 |
| *D. anacardii* | CBS 720.97* | *Anacardium ocidentale* | East Africa | KC343024 | KC343992 | KC343750 | KC343508 | KC343266 |
|  | CBS 144610 | unidentified leaf | South Africa | MK442578 | - | MK442692 | - | MK442651 |
| *D. angelicae* | CBS 111592* | *Heracleum sphondylium* | Austria | KC343027 | KC343995 | KC343753 | KC343511 | KC343269 |
| *D. arctii* | CBS 139280 = DP0482* | *Arctium lappa* | Austria | KJ590736 | KJ610891 | KJ590776 | KJ659218 | KJ612133 |
| *D. arecae* | CBS 161.64* | *Areca catechu* | India | KC343032 | KC344000 | KC343758 | KC343516 | KC343274 |
| *D. arengae* | CBS 114979* | *Arenga engleri* | Hong Kong | KC343034 | KC344002 | KC343760 | KC343518 | KC343276 |
| *D. asheicola* | CBS 136967* | *Vaccinium ashei* | Chile | KJ160562 | KJ160518 | KJ160594 | - | KJ160542 |
|  | CBS 136968 | *V. ashei* | Chile | KJ160563 | KJ160519 | KJ160595 | - | KJ160543 |
| *D. australafricana* | CBS 113487* | *V. vinifera* | South Africa | KC343039 | KC344007 | KC343765 | KC343523 | KC343281 |
|  | CBS 111886 | *V. vinifera* | Australia | KC343038 | KC344006 | KC343764 | KC343522 | KC343280 |
| *D. baccae* | CBS 136972* | *Vaccinium corymbosum* | Italy | KJ160565 | MF418509 | KJ160597 | MF418264 | MG281695 |
| *D. batatas* | CBS 122.21* | *Ipomoea batatas* | USA | KC343040 | KC344008 | KC343766 | KC343524 | KC343282 |
| *D. beilharziae* | BRIP 54792* | *Indigofera australis* | Australia | JX862529 | KF170921 | JX862535 | - | - |
| *D. biguttulata* | CGMCC 3.17248 = ZJUD47* | *Citrus limon* | China | KJ490582 | KJ490403 | KJ490461 | KJ490524 | - |
| *D. bohemiae* | CBS 143347* | *Vitis* spp. | Czech Republic | MG281015 | MG281188 | MG281536 | MG281361 | MG281710 |
| *D. brasiliensis* | CBS 133183* | *Aspidosperma tomentosum* | Brazil | KC343042 | KC344010 | KC343768 | KC343526 | KC343284 |
|  | LGMF926 | *A. tomentosum* | Brazil | KC343043 | KC344011 | KC34376 | KC343527 | KC343285 |
| *D. caatingaensis* | CBS 141542 = URM7486* | *Tacinga inamoena* | Brazil | KY085927 | KY115600 | KY115603 | - | - |
| *D. carpini* | CBS 114437* | *Carpinus betulus* | Sweden | KC343044 | KC344012 | KC343770 | KC343528 | KC343286 |
| *D. caryae* | CFCC 52563* | *Carya illinoensis* | China | MH121498 | MH121580 | MH121540 | MH121458 | MH121422 |
|  | CFCC 52564 | *C. illinoensis* | China | MH121499 | MH121581 | MH121541 | MH121459 | MH121423 |
|  | PSCG 528 | *P. pyrifolia* | China | MK626953 | MK691316 | MK654896 | MK726203 | MK691201 |
|  | PSCG 380 | *P. pyrifolia* | China | MK626951 | MK691313 | MK654893 | MK726200 | MK691198 |
| *D. caulivora* | CBS 127268* | *Glycine max* | Croatia | KC343045 | KC344013 | KC343771 | KC343529 | KC343287 |
| *D. cercidis* | CFCC 52565* | *Cercis chinensis* | China | MH121500 | MH121582 | MH121542 | MH121460 | MH121424 |
| ***D. cerradensis*** | **CMRP4331** | ***Stryphnodendron adstringens*** | **Brazil** | [**MN173198**](https://www.ncbi.nlm.nih.gov/nuccore/MN173193) | **MW751671** | **MT311685** | **MW751663** | **MW751655** |
| ***D. cerradensis*** | **CMRP4324** | ***S. adstringens*** | **Brazil** | [**MN173195**](https://www.ncbi.nlm.nih.gov/nuccore/MN173193) | **MW751667** | **MT311681** | **MW751659** | **MW751651** |
| *D. cerradensis = D.* cf. *mayteni* | UFMGCB4807 | *Carapa guianensis* | Brazil | KJ677018 | KP189348 | KP189356 | - | - |
| *D. cerradensis = Diaporthe* sp. | LGMF1616 | *S. adstringens* | Brazil | MG976419 | - | - | - | - |
| *D. chamaeropis* | CBS 454.81* | *Chamaerops humilis* | Greece | KC343048 | KC344016 | KC343774 | KC343532 | KC343290 |
| *D. cinnamomi* | CFCC 52569* | *Cinnamomum* sp. | China | MH121504 | MH121586 | MH121546 | MH121464 | - |
|  | CFCC 52570 | *Cinnamomum* sp. | China | MH121505 | MH121587 | MH121547 | MH121465 | - |
| *D. citriasiana* | ZJUD 30* | *Citrus unshiu* | China | JQ954645 | KC357459 | JQ954663 | - | KC357491 |
| *D. compactum* | LC3083 | *Camellia sinensis* | China | KP267854 | KP293434 | KP267928 | KP293508 | - |
| *D. convolvuli* | CBS 124654 = DP0727* | *Convolvulus arvensis* | Turkey | KC343054 | KC344022 | KC343780 | KC343538 | KC343296 |
| *D. crataegi* | CBS 114435* | *Crataegus rhipidophylla* | Sweden | KC343055 | KC344023 | KC343781 | KC343539 | KC343297 |
| *D. cuppatea* | CBS 117499 = STE-U 5431* | *Aspalathus linearis* | South Africa | KC343057 | KC344025 | KC343783 | KC343541 | KC343299 |
| *D. cytosporella* | FAU461* | *Citrus limon* | Italy | KC843307 | KC843221 | KC843116 | MF418283 | KC843141 |
| *D. detrusa* | CBS 109770 | *Berberis vulgaris* | Austria | KC343061 | KC344029 | KC343787 | KC343545 | KC343303 |
| *D. discoidispora* | ZJUD89* = CGMCC 3.17255 | *Citrus unshiu* | China | KJ490624 | KJ490445 | KJ490503 | KJ490566 | - |
|  | ZJUD87 | *Citrus sinensis* | China | KJ490622 | KJ490443 | KJ490501 | KJ490564 | - |
| *D. dorycnii* | MFLUCC 17-1015* | *Dorycnium hirsutum* | Italy | KY964215 | KY964099 | KY964171 | - | - |
| *D. endophytica* | CBS 133811 = LGMF916* | *Schinus terebinthifolius* | Brazil | KC343065 | KC344033 | KC343791 | KC343549 | KC343307 |
|  | LGMF911 | *S. terebinthifolius* | Brazil | KC343066 | KC344034 | KC343792 | KC343550 | KC343308 |
| *D. eugeniae* | CBS 444.82 | *Eugenia aromatica* | Indonesia | KC343098 | KC344066 | KC343824 | KC343582 | KC343340 |
| *D. fibrosa* | CBS 109751 | *Rhamnus cathartica* | Austria | KC343099 | KC344067 | KC343825 | KC343583 | KC343341 |
| *D. foeniculina* | CBS 111553* | *Foeniculum vulgare* | Spain | KC343101 | KC344069 | KC343827 | KC343585 | KC343343 |
| *D. fraxini-angustifoliae* | MFLUCC 15-0748 | *V. vinifera* | China | KT459428 | KT459430 | KT459446 | - | KT459462 |
| *D. fulvicolor* | PSCG 051* | *P. pyrifolia* | China | MK626859 | MK691236 | MK654806 | MK726163 | MK691132 |
| *D. ganjae* | CBS 180.91* | *Cannabis sativa* | USA | KC343112 | KC344080 | KC343838 | KC343596 | KC343354 |
| *D. goulteri* | BRIP 55657a* | *Helianthus annuus* | Australia | KJ197290 | KJ197270 | KJ197252 | - | - |
| *D. gulyae* | BRIP 54025* | *H. annuus* | Australia | JF431299 | - | JN645803 | - | - |
| *D. helianthi* | CBS 592.81* | *H. annuus* | Serbia | KC343115 | KC344083 | KC343841 | KC343599 | KC343357 |
|  | CBS 344.94 | *H. annuus* | - | KC343114 | KC344082 | KC343840 | KC343598 | KC343356 |
| *D.* cf. *heveae* 1 | CBS 852.97 | *Hevea brasiliensis* | Brazil | KC343116 | KC344084 | KC343842 | KC343600 | KC343358 |
|  | CP 2019 Tg12 | *Tibouchina granulosa* | Brazil | MN148263 | MN151275 | MN151292 | - | - |
|  | CP 2019 Tg180 | *T. granulosa* | Brazil | MN148284 | MN151274 | MN151293 | - | - |
|  | LGMF1631 | *S. adstringens* | Brazil | MG976433 | MK007530 | MK007529 | - | - |
|  | **CMRP4329** | ***S. adstringens*** | **Brazil** | [**MN173197**](https://www.ncbi.nlm.nih.gov/nuccore/MN173193) | **MW751669** | **MT311683** | **MW751661** | **MW751653** |
| *D. hispaniae* | CBS 143351 = CPC 303213* | *V. vinifera* | Spain | MG281123 | MG281296 | MG281644 | MG281471 | MG281820 |
| *D. hongkongensis* | CBS 115448* | *Dichroa febrífuga* | China | KC343119 | KC344087 | KC343845 | KC343603 | KC343361 |
| *D. hubeiensis* | JZB320123* | *Vitis vinifera* | China | MK335809 | MK500148 | MK523570 | - | MK500235 |
| *D. hungariae* | CBS 143353 = CPC 301303* | *V. vinifera* | Hungary | MG281126 | MG281299 | MG281647 | MG281474 | MG281823 |
| *D. impulsa* | CBS 114434* | *Sorbus aucuparia* | Sweden | KC343121 | KC344089 | KC343847 | KC343605 | KC343363 |
| *D. inconspicua* | CBS 133813* | *Maytenus ilicifolia* | Brazil | KC343123 | KC344091 | KC343849 | KC343607 | KC343365 |
| *D. infecunda* | LGMF912 = CPC 20288* | *Schinus terebinthifolius* | Brazil | KC343128 | KC344096 | KC343854 | KC343612 | KC343370 |
| *D. kongii* | BRIP 54031* | *Helianthus annuus* | Australia | JF431301 | - | JN645797 | - | - |
| *D. limonicola* | CPC 28200 = CBS 142549* | *Citrus limon* | Malta | MF418422 | MF418582 | MF418501 | MF418342 | MF418256 |
| *D. litchicola* | BRIP 54900* | *Litchi chinensis* | Australia | JX862533 | KF170925 | JX862539 | - | - |
| *D. lithocarpus* | CGMCC 3.15175* | *Lithocarpus glabra* | China | KC153104 | KF576311 | KC153095 | - | KF576235 |
| *D. longicolla* | FAU599* | *Glycine max* | USA | KJ590728 | KJ610883 | KJ590767 | KJ659188 | KJ612124 |
|  | FAU644 | *G. max* | USA | KJ590730 | KJ610885 | KJ590769 | KJ659190 | KJ612126 |
| *D. longispora* | CBS 194.36* | *Ribes* sp. | Canada | KC343135 | KC344103 | KC343861 | KC343619 | KC343377 |
| *D. lusitanicae* | CBS 123212* | *Foeniculum vulgare* | Portugal | KC343136 | KC344104 | KC343862 | KC343620 | KC343378 |
| *D. macadamiae* | BRIP 66526* | *Macadamia* sp. | South Africa | MN708230 | MN696539 | MN696528 | - | - |
| *D. malorum* | CBS 142383 = CAA734* | *Malus domestica* | Portugal | KY435638 | KY435668 | KY435627 | KY435648 | KY435658 |
| *D. manihotia* | CBS 505.76 | *Manihot utilissima* | Rwanda | KC343138 | KC344106 | KC343864 | KC343622 | KC343380 |
| *D. masirevicii* | BRIP 57892a* | *Helianthus annuus* | Australia | KJ197277 | KJ197257 | KJ197239 | - | - |
|  | BRIP 54256 | *Glycine max* | Australia | KJ197277 | KJ197256 | KJ197238 | - | - |
| *D. mayteni* | CBS 133185 | *Maytenus ilicifolia* | Brazil | KC343139 | KC344107 | KC343865 | KC343623 | KC343139 |
| *D. melitensis* | CPC 27873 = CBS 142551* | *C. limon* | Malta | MF418424 | MF418584 | MF418503 | MF418344 | MF418258 |
| *D. melonis* | CBS 507.78* | *Glycine soja* | USA | KC343141 | KC344109 | KC343867 | KC343625 | KC343383 |
|  | CBS 435.87 | *G. soja* | Indonesia | KC343141 | KC344109 | KC343867 | KC343625 | KC343383 |
| *D. middletonii* | BRIP 54884e* | *Rapistrum rugostrum* | Australia | KJ197286 | KJ197266 | KJ197248 | - | - |
| *D. miriciae* | BRIP 54736j* | *H. annuus* | Australia | KJ197282 | KJ197262 | KJ197244 | - | - |
|  | BRIP 55662c | *G. max* | Australia | KJ197283 | KJ197263 | KJ197245 | - | - |
| *D. multigutullata* | CGMCC 3.17258 = ZJUD98* | *Citrus grandis* | China | KJ490633 | KJ490454 | KJ490512 | KJ490575 | - |
| *D. musigena* | CBS 129519* | *Musa* sp. | Australia | KC343143 | KC344111 | KC343869 | KC343627 | KC343385 |
| *D. nebulae* | PMM1681* | *Vitis vinifera* | South Africa | KY511337 | KY511369 | MH708552 | - | - |
|  | Phom240 | *V. vinifera* | South Africa | KY511315 | KY511346 | MH708543 | - | - |
| *D. novem* | CBS 127270* | *G. max* | Croatia | KC343156 | KC344124 | KC343882 | KC343640 | KC343398 |
| *D. neoarctii* | CBS 109490* | *Ambrosia trifida* | USA | KC343145 | KC344113 | KC343871 | KC343629 | KC343387 |
| *D. neoraonikayaporum* | MFLUCC 14-1136 | *Tectona grandis* | Thailand | KU712449 | KU743988 | KU749369 | - | KU749356 |
|  | MFLUCC 14-1133 | *T. grandis* | Thailand | KU712448 | KU743987 | KU749368 | - | KU749355 |
| *D. neotheicola* | CBS 123209 | *Foeniculum vulgare* | Portugal | GQ250192 | - | GQ250316 | - | - |
| *D. oxe* | CBS 133186* | *Maytenus ilicifolia* | Brazil | KC343164 | KC344132 | KC343890 | KC343648 | KC343406 |
|  | CBS 133187 | *M. ilicifolia* | Brazil | KC343165 | KC344133 | KC343891 | KC343649 | KC343407 |
| *D. paranensis* | CBS 133184* | *M. ilicifolia* | Brazil | KC343171 | KC344139 | KC343897 | KC343655 | KC343413 |
| *D. parvae* | PSCG 034* | *Pyrus bretschneideri* | China | MK626919 | MK691248 | MK654858 | MK726210 | - |
|  | PSCG 035 | *P. bretschneideri* | China | MK626920 | MK691249 | MK654859 | MK726211 | MK691169 |
| *D. pascoei* | BRIP 54847* | *Persea americana* | Australia | JX862532 | KF170924 | JX862538 | - | - |
| *D. passiflorae* | CBS 132527* | *Passiflora edulis* | South America | JX069860 | - | - | KY435654 | - |
| *D. passifloricola* | CBS 141329* | *Passiflora foetida* | Malaysia | KX228292 | KX228387 | - | KX228367 | - |
| *D. pescicola* | MFLUCC 16-0105* | *Prunus persica* | China | KU557555 | KU557579 | KU557623 | - | KU557603 |
| *D. perjuncta* | CBS 109745* | *Ulmus glabra* | Austria | KC343172 | KC344140 | KC343898 | KC343656 | KC343414 |
| *D. perseae* | CBS 151.73* | *Persea gratissima* | Netherlands | KC343173 | KC344141 | KC343899 | KC343657 | KC343415 |
| *D. phillipsii* | CAA817* | *Vaccinium corymbosum* | Portugal | MK792305 | MN000351 | MK828076 | MK871445 | MK883831 |
|  | CAA818 | *V. corymbosum* | Portugal | MK792307 | MN000352 | MK828078 | MK871447 | MK883833 |
| *D. podocarpi-macrophylli* | LC6200 | *Podocarpus macrophyllus* | China | KX986769 | KX999201 | KX999161 | KX999240 | KX999276 |
| *D. portugallica* | CPC 34247* | *Camellia sinensis* | Portugal | MH063905 | MH063917 | MH063911 | MH063899 | MH063893 |
|  | CPC 34248 | *C. sinensis* | Portugal | MH063906 | MH063918 | MH063912 | MH063900 | MH063894 |
| *D. pseudophoenicicola* | CBS 462.69* | *Phoenix dactylifera* | Spain | KC343183 | KC344151 | KC343909 | KC343667 | KC343425 |
| *D. pseudomangiferae* | CBS 101339* | *Mangifera indica* | Dominican Republic | KC343181 | KC344149 | KC343907 | KC343665 | KC343423 |
| *D. pterocarpi* | MFLUCC 10-0571* | *Pterocarpus indicus* | Thailand | JQ619899 | JX275460 | JX275416 | - | JX197451 |
| *D. pterocarpicola* | MFLUCC 10-0580a* | *P. indicus* | Thailand | JQ619887 | JX275441 | JX275403 | - | JX197433 |
| *D. raonikayaporum* | CBS 133182* | *Spondias mombin* | Brazil | KC343188 | KC344156 | KC343914 | KC343672 | KC343430 |
| *D. ravennica* | MFLUCC 15–0480 | *Tamarix sp.* | Italy | KU900336 | KX377688 | KX426703 | - | - |
| *D. rhusicola* | CBS 129528* | *Rhus pendulina* | South Africa | JF951146 | KC843205 | KC843100 | - | KC843124 |
| *D. rudis* | CBS 113201* | *V. vinifera* | Portugal | KC343234 | KC344202 | KC343960 | KC343718 | KC343476 |
|  | CBS 114011 | *V. vinifera* | Portugal | KC343235 | KC344203 | KC343961 | KC343719 | KC343477 |
|  | CBS 143346 | *V. vinifera* | Czech Republic | MG281131 | MG281304 | MG281652 | MG281479 | MG281828 |
|  | CBS 266.85 | *Rosa rugosa* | Netherlands | KC343237 | KC344205 | KC343721 | KC343721 | KC343479 |
|  | CBS 794.96 | *Aucuba japonica* | United Kingdom | KC343243 | KC344211 | KC343969 | KC343727 | KC343485 |
| *D. sackstonii* | BRIP 54669b* | *Helianthus annuus* | Australia | KJ197287 | KJ197267 | KJ197249 | - | - |
| *D. sambucusii* | CFCC 51986* | *Sambucus williamsii* | China | KY852495 | KY852511 | KY852507 | KY852503 | KY852499 |
|  | CFCC 51987 | *S. williamsii* | China | KY852496 | KY852512 | KY852508 | KY852504 | KY852500 |
| *D. sclerotioides* | CBS 296.67* | *Cucumis sativus* | Netherlands | KC343193 | KC344161 | KC343919 | KC343677 | KC343435 |
|  | CBS 710.76 | *C. sativus* | Netherlands | KC343194 | KC344162 | KC343920 | KC343678 | KC343436 |
| *D. schini* | CBS 133181* | *S. terebinthifolius* | Brazil | KC343191 | KC344159 | KC343917 | KC343675 | KC343433 |
|  | LGMF910 | *S. terebinthifolius* | Brazil | KC343192 | KC344160 | KC343918 | KC343676 | KC343434 |
| *D. sennae* | CFCC 51636* | *Senna bicapsularis* | China | KY203724 | KY228891 | KY228885 | KY228879 | KY228875 |
| *D. serafiniae* | BRIP 55665a* | *H. annuus* | Australia | KJ197274 | KJ197254 | KJ197236 | - | - |
| *D. siamensis* | MFLUCC 10 0573a | *Dasymaschalon sp.* | Thailand | JQ619879 | JX275429 | JX275393 | - | - |
| *D. sojae* | FAU635* | *G. max* | USA | KJ590719 | KJ610875 | KJ590762 | KJ659208 | KJ612116 |
|  | FAU455 | *Stokesia laevis* | USA | KJ590712 | KJ610868 | KJ590755 | KJ659201 | KJ612109 |
|  | PSCG 177 | *P. pyrifolia* | China | MK626940 | MK691302 | MK654882 | MK726189 | MK691188 |
|  | PSCG 518 | *P. pyrifolia* | China | MK626945 | MK691312 | MK654883 | MK726198 | MK691192 |
| ***Diaporthe* sp.** | **CMRP4330** | ***Vochysia divergens*** | **Brazil** | [**MN173199**](https://www.ncbi.nlm.nih.gov/nuccore/MN173193) | **MW751670** | **MT311684** | **MW751662** | **MW751654** |
| *Diaporthe* sp.1 | CBS119639* | *Homo sapiens* | Germany | KC343202 | KC344170 | KC343928 | KC343686 | KC343444 |
| *Diaporthe* sp.2 | LGMF932 | *Maytenus ilicifolia* | Brazil | KC343204 | KC344172 | KC343930 | KC343688 | KC343446 |
| *Diaporthe* sp.3 | CBS 287.29 | *Pseudotsuga menziesii* | Scotland | KC343205 | KC344173 | KC343931 | KC343689 | KC343447 |
| *Diaporthe* sp.4 | LGMF944 | *Maytenus ilicifolia* | Brazil | KC343206 | KC344174 | KC343932 | KC343690 | KC343448 |
| *Diaporthe* sp.5 | CBS 125575 | *Acer opalus* | Italy | KC343207 | KC344175 | KC343933 | KC343691 | KC343449 |
| *D. spinosa* | PSCG 383* | *P. pyrifolia* | China | MK626849 | MK691234 | MK654811 | MK726156 | MK691129 |
| *D. sterilis* | CBS 136969* | *Vaccinium corymbosum* | Italy | KJ160579 | KJ160528 | KJ160611 | MF418350 | KJ160548 |
| *D. stewartii* | CBS 193.36 | *Cosmos bipinnatus* | USA | FJ889448 | JX275421 | GQ250324 | - | JX197415 |
| *D. subordinaria* | CBS 464.90* | *Plantago lanceolata* | New Zealand | KC343214 | KC344182 | KC343940 | KC343698 | KC343456 |
| *D. taoicola* | MFLUCC 16-0117* | *Prunus persica* | China | KU557567 | KU557591 | KU557635 | - | - |
|  | PSCG 292 | *P. pyrifolia* | China | MK626871 | MK691232 | MK654800 | MK726168 | MK691115 |
| *D. tectonae* | MFLUCC 12-0777* | *Tectona grandis* | China | KU712430 | KU743977 | KU749359 | - | KU749345 |
| *D. tectonendophytica* | MFLUCC 13–0471* | *T. grandis* | China | KU712439 | KU743986 | KU749367 | KX999266 | KU749354 |
| *D. terebinthifolii* | CBS 133180* | *S. terebinthifolius* | Brazil | KC343216 | KC344184 | KC343942 | KC343700 | KC343458 |
|  | LGMF907 | *S. terebinthifolius* | Brazil | KC343217 | KC344185 | KC343943 | KC343701 | KC343459 |
| *D. thunbergiicola* | MFLUCC 12–0033* | *Thunbergia laurifolia* | Thailand | KP715097 | - | KP715098 | - | - |
| *D. tulliensis* | BRIP 62248a* | *Theobroma cacao* | Australia | KR936130 | KR936132 | KR936133 | - | - |
| *D. ueckerae* | FAU656* | *Cucumis melo* | USA | KJ590726 | KJ610881 | KJ590747 | KJ659215 | KJ612122 |
| *D. unshiuensis* | ZJUD52* | *Citrus* sp. | China | KJ490587 | KJ490408 | KJ490466 | KJ490529 | - |
|  | ZJUD49 | *Citrus* sp. | China | KJ490584 | KJ490405 | KJ490463 | KJ490526 | - |
|  | CFCC 52595 | *Carya illinoinensis* | China | MH121530 | MH121607 | MH121572 | MH121488 | - |
|  | PSCG120 | *P. pyrifolia* | China | MK626926 | MK691288 | MK654868 | MK726174 | MK691174 |
|  | PSCG131 | *P. pyrifolia* | China | MK626934 | MK691293 | MK654869 | MK726176 | MK691176 |
|  | ZJUD50 | *Citrus japonica* | China | KJ490585 | KJ490406 | KJ490464 | KJ490527 | - |
| *D. velutina* | CGMCC 3.18286  =LC 4421* | *Neolitsea* sp. | China | KX986790 | KX999223 | KX999182 | - | - |
| *D. vexans* | CBS 127.14 | *Solanum melongena* | USA | KC343229 | KC344197 | KJ590774 | KC34371 | KC343471 |
| *D. vochysiae* | LGMF1583* | *V. divergens* | Brazil | MG976391 | MK007527 | MK007526 | MK033323 | MK007528 |
|  | **CMRP4321** | ***V. divergens*** | **Brazil** | [**MN173193**](https://www.ncbi.nlm.nih.gov/nuccore/MN173193) | **MW751665** | **MT311679** | **MW751657** | **MW751649** |
|  | **CMRP4322** | ***V. divergens*** | **Brazil** | [**MN173194**](https://www.ncbi.nlm.nih.gov/nuccore/MN173193) | **MW751666** | **MT311680** | **MW751658** | **MW751650** |
|  | **CMRP4326** | ***S. adstringens*** | **Brazil** | [**MN173196**](https://www.ncbi.nlm.nih.gov/nuccore/MN173193) | **MW751668** | **MT311682** | **MW751660** | **MW751652** |
|  | **CMRP4332** | ***V. divergens*** | **Brazil** | [**MN173200**](https://www.ncbi.nlm.nih.gov/nuccore/MN173193) | **MW751672** | **MT311686** | **MW751664** | **MW751656** |
| *D. zaobaisu* | PSCG 031* | *Pyrus bretschneideri* | China | MK626922 | MK691245 | MK654855 | MK726207 | - |
| *D. yunnanensis* | CGMCC 3.18289 =LC6168* | *Coffea* sp. | China | KX986796 | KX999228 | KX999188 | KX999267 | KX999290 |

^1^Collection – Type strains included in analysis are indicated with *. Culture collections abbreviations: BRIP = Australian plant pathogen culture collection, Queensland, Australia; CAA = Personal Culture Collection of Artur Alves, Universidade de Aveiro, Portugal; CBS = Culture Collection of the Westerdijk Fungal Biodiversity Institute, Utrecht, The Netherlands; CGMCC = China General Microbiological Culture Collection; CMRP = Microbiological Collections of Paraná Network, Federal University of Paraná, Curitiba, Brazil; CPC = Culture Collection of Pedro Crous, housed at CBS; CFCC: China Forestry Culture Collection Center, China; FAU: Isolates in culture collection of Systematic Mycology and Microbiology Laboratory, USDA-ARS, Beltsville, MD, USA; LC = Working collection of Lei Cai, housed at Institute of Microbiology, CAS, China; LGMF = Culture Collection of Laboratory of Genetics of Microorganisms, Federal University of Paraná, Curitiba, Brazil; MFLUCC = Mae Fah Luang University Culture Collection; UFMGCB = Coleção de Microrganismos e Células da Universidade Federal de Minas Gerais; ZJUD: Zhejiang University.

^2^GenBank - ITS: internal transcribed spacers and intervening 5.8S nrDNA; *tub2:* partial beta-tubulin gene; *tef1*: translation elongaton factor 1-α; *his3:* partial histone H3 gene, and *cal*: partial calmodulin gene.

-: Sequence not available.

In bold the isolates of this study;

Table S2. Screening of antifungal activity evaluated against the pathogen *Colletotrichum abscissum*. The fungicide Carbendazim (1.0 mg/mL) was used as positive control and methanol as negative control.

| Phenotype | Strain | Mean mycelial growth | Inhibition rate (%) | Significance |
| --- | --- | --- | --- | --- |
| 1 | CMRP4506 | 4.9 | 2 | - |
| 2 | CMRP4507 | 3.9 | 21.3 | - |
| 3 | CMRP4508 | 3.9 | 22 | - |
| 4 | CMRP4509 | 3.8 | 24.7 | - |
| 5 | CMRP4510 | 4.7 | 5.3 | - |
| 6 | CMRP4511 | 4.6 | 8 | - |
| 7 | CMRP4512 | 5.0 | 0 | - |
| 8 | CMRP4513 | 4.1 | 18 | - |
| 9 | CMRP4514 | 4.0 | 19.3 | - |
| 10 | CMRP4515 | 3.7 | 26.7 | - |
| 11 | CMRP4516 | 4.2 | 15.3 | - |
| 12 | CMRP4517 | 3.5 | 29.3 | - |
| 13 | CMRP4518 | 4.6 | 8 | - |
| 14 | CMRP4519 | 3.3 | 33.3 | * |
| 15 | CMRP4520 | 4.0 | 19.3 | - |
| 16 | CMRP4521 | 4.8 | 4 | - |
| 17 | CMRP4522 | 4.7 | 6 | - |
| 18 | CMRP4523 | 3.9 | 21.3 | - |
| 19 | CMRP4524 | 4.5 | 10.7 | - |
| 20 | CMRP4525 | 4.1 | 18.7 | - |
| 21 | CMRP4526 | 4.6 | 8 | - |
| 22 | CMRP4527 | 4.1 | 18.7 | - |
| 23 | CMRP4528 | 4.7 | 6.7 | - |
| 24 | CMRP4529 | 4.1 | 18 | - |
| 25 | CMRP4530 | 3.4 | 31.3 | * |
| 26 | CMRP4531 | 4.2 | 15.3 | - |
| 27 | CMRP4532 | 4.4 | 12 | - |
| 28 | CMRP4533 | 4.5 | 10 | - |
| 29 | CMRP4534 | 4.4 | 11.3 | - |
| 30 | CMRP4535 | 4.4 | 12 | - |
| 31 | CMRP4536 | 3.8 | 23.3 | - |
| 32 | CMRP4537 | 4.3 | 14.7 | - |
| 33 | CMRP4538 | 4.2 | 16.7 | - |
| 34 | CMRP4539 | 4.0 | 20.7 | - |
| 35 | CMRP4540 | 4.6 | 7.3 | - |
| 36 | CMRP4541 | 4.2 | 15.3 | - |
| 37 | CMRP4542 | 3.8 | 24 | - |
| 38 | CMRP4543 | 4.0 | 20.7 | - |
| 39 | CMRP4544 | 4.5 | 9.3 | - |
| 40 | CMRP4545 | 4.1 | 17.3 | - |
| 41 | CMRP4546 | 4.1 | 17.3 | - |
| 42 | CMRP4547 | 4.5 | 9.3 | - |
| 43 | CMRP4548 | 4.5 | 10 | - |
| 44 | CMRP4549 | 4.5 | 10.7 | - |
| 45 | CMRP4550 | 4.5 | 10 | - |
| 46 | CMRP4551 | 4.3 | 13.3 | - |
| 47 | CMRP4552 | 3.7 | 25.3 | - |
| 48 | CMRP4553 | 3.8 | 24.7 | - |
| 49 | CMRP4554 | 4.5 | 10 | - |
| 50 | CMRP4555 | 4.7 | 6.7 | - |
| 51 | CMRP4573 | 4.0 | 20 | - |
| 52 | CMRP4574 | 4.2 | 15.3 | - |
| 53 | CMRP4575 | 4.3 | 14.7 | - |
| 54 | CMRP4576 | 4.2 | 15.3 | - |
| 55 | CMRP4577 | 3.3 | 34.7 | * |
| 56 | CMRP4578 | 4.3 | 13.3 | - |
| 57 | CMRP4579 | 4.4 | 11.3 | - |
| 58 | CMRP4580 | 4.6 | 7.3 | - |
| 59 | CMRP4581 | 4.0 | 20.7 | - |
| 60 | CMRP4582 | 4.3 | 14 | - |
| 61 | CMRP4583 | 4.3 | 13.3 | - |
| 62 | CMRP4584 | 4.6 | 8.7 | - |
| **63** | **CMRP4321** | **2.7** | **46** | ****** |
| 64 | CMRP4585 | 3.6 | 27.3 | - |
| 65 | CMRP4586 | 4.4 | 12 | - |
| 66 | CMRP4587 | 3.7 | 25.3 | - |
| **67** | **CMRP4322** | **1.2** | **75.3** | ****** |
| **68** | **CMRP4323** | **1.8** | **80** | ******* |
| 69 | CMRP4588 | 4.0 | 20 | - |
| 70 | CMRP4589 | 4.4 | 12.7 | - |
| 71 | CMRP4590 | 4.4 | 12 | - |
| **72** | **CMRP4324** | **2.6** | **48.7** | ****** |
| 73 | CMRP4591 | 4.4 | 12 | - |
| 74 | CMRP4592 | 4.4 | 12 | - |
| 75 | CMRP4593 | 4.2 | 16.7 | - |
| 76 | CMRP4594 | 3.4 | 31.3 | * |
| 77 | CMRP4595 | 4.2 | 15.3 | - |
| 78 | CMRP4596 | 4.4 | 12 | - |
| 79 | CMRP4597 | 4.0 | 20 | - |
| 80 | CMRP4598 | 3.6 | 28.7 | - |
| 81 | CMRP4669 | 4.2 | 16 | - |
| 82 | CMRP4599 | 4.3 | 13.3 | - |
| 83 | CMRP4600 | 4.3 | 14.7 | - |
| 84 | CMRP4601 | 4.4 | 12.7 | - |
| 85 | CMRP4602 | 3.2 | 35.3 | * |
| 86 | CMRP4603 | 3.5 | 29.3 | * |
| 87 | CMRP4604 | 3.4 | 32.7 | * |
| 88 | CMRP4605 | 4.1 | 18 | - |
| 89 | CMRP4606 | 3.8 | 24 | - |
| 90 | CMRP4607 | 4.5 | 9.3 | - |
| 91 | CMRP4608 | 4.2 | 16.7 | - |
| 92 | CMRP4609 | 4.5 | 10.7 | - |
| 93 | CMRP4610 | 3.6 | 27.3 | - |
| 94 | CMRP4611 | 4.2 | 16 | - |
| 95 | CMRP4612 | 3.5 | 29.3 | * |
| 96 | CMRP4613 | 4.0 | 20.7 | - |
| 97 | CMRP4614 | 4.0 | 19.3 | - |
| 98 | CMRP4615 | 3.4 | 32 | * |
| 99 | CMRP4616 | 4.4 | 11.3 | - |
| 100 | CMRP4617 | 4.1 | 18 | - |
| 101 | CMRP4618 | 4.4 | 11.3 | - |
| 102 | CMRP4619 | 4.2 | 16 | - |
| 103 | CMRP4620 | 4.6 | 8 | - |
| 104 | CMRP4621 | 4.3 | 14.7 | - |
| 105 | CMRP4622 | 3.4 | 32 | * |
| 106 | CMRP4623 | 4.0 | 20.7 | - |
| 107 | CMRP4624 | 4.0 | 20.7 | - |
| 108 | CMRP4625 | 4.4 | 12 | - |
| 109 | CMRP4626 | 4.2 | 16 | - |
| 110 | CMRP4627 | 4.0 | 19.3 | - |
| 111 | CMRP4628 | 3.7 | 26 | - |
| 112 | CMRP4629 | 3.9 | 22 | - |
| 113 | CMRP4630 | 4.4 | 12.7 | - |
| 114 | CMRP4631 | 4.2 | 16.7 | - |
| 115 | CMRP4632 | 4.0 | 20.7 | - |
| 116 | CMRP4633 | 4.5 | 10 | - |
| 117 | CMRP4634 | 4.2 | 15.3 | - |
| 118 | CMRP4635 | 4.0 | 20 | - |
| 119 | CMRP4636 | 4.1 | 18 | - |
| 120 | CMRP4637 | 3.3 | 34 | * |
| 121 | CMRP4638 | 4.3 | 14 | - |
| 122 | CMRP4639 | 3.7 | 26 | - |
| 123 | CMRP4640 | 4.2 | 15.3 | - |
| 124 | CMRP4641 | 4.6 | 8.7 | - |
| 125 | CMRP4642 | 4.3 | 14 | - |
| **126** | **CMRP4325** | **1.4** | **72.7** | ****** |
| 127 | CMRP4643 | 3.3 | 34.7 | * |
| **128** | **CMRP4326** | **2.7** | **46** | ****** |
| **129** | **CMRP4327** | **2.4** | **51.3** | ****** |
| 130 | CMRP4644 | 4.3 | 14.7 | - |
| 131 | CMRP4645 | 3.8 | 23.3 | - |
| 132 | CMRP4646 | 4.3 | 13.3 | - |
| **133** | **CMRP4328** | **0.9** | **82.7** | ******* |
| 134 | CMRP4647 | 4.1 | 18 | - |
| 135 | CMRP4648 | 3.1 | 37.3 | - |
| 136 | CMRP4649 | 4.2 | 15.3 | - |
| 137 | CMRP4650 | 4.3 | 13.3 | - |
| 138 | CMRP4651 | 4.1 | 17.3 | - |
| 139 | CMRP4652 | 4.1 | 17.3 | - |
| **140** | **CMRP4329** | **2.8** | **40.3** | ****** |
| 141 | CMRP4653 | 3.6 | 28 | - |
| 142 | CMRP4654 | 3.8 | 24 | - |
| 143 | CMRP4655 | 4.4 | 11.3 | - |
| 144 | CMRP4656 | 3.4 | 32 | * |
| **145** | **CMRP4330** | **1.4** | **71.3** | ****** |
| 146 | CMRP4657 | 4.6 | 7.3 | - |
| 147 | CMRP4658 | 4.3 | 14.7 | - |
| 148 | CMRP4659 | 3.6 | 27.3 | - |
| 149 | CMRP4660 | 4.5 | 9.3 | - |
| 150 | CMRP4661 | 4.6 | 8.7 | - |
| 151 | CMRP4662 | 4.6 | 8 | - |
| **152** | **CMRP4331** | **2.9** | **40** | ****** |
| 153 | CMRP4663 | 3.6 | 27.3 | - |
| 154 | CMRP4664 | 4.5 | 10 | - |
| 155 | CMRP4665 | 4.5 | 10.7 | - |
| 156 | CMRP4666 | 4.0 | 20.7 | - |
| 157 | CMRP4667 | 4.3 | 14.7 | - |
| **158** | **CMRP4332** | **2.0** | **65.3** | ****** |
| 159 | CMRP4668 | 4.4 | 11.3 | - |
| Methanol |  | 5.0 | 0 | - |
| Carbendazim |  | 2.6 | 47.3 | - |

*p=0.05 **p=0.01 ***p<0.01 - not significant;

In bold 12 isolates with the best results of activity against the pathogen *C. abscissum*.

Table S3. Endophytic fungi used in this study isolated from the medicinal plants *Stryphnodendron adstringens* (*S.a*) and *Vochysia divergens* (*V.d*) from leaves or petioles.

| **Class** | **Order** | **Identification** | **Strain** | **Host** | **Tissue** |
| --- | --- | --- | --- | --- | --- |
| Sordariomycetes | Diaporthales | *Diaporthe vochysiae* | CMRP4321 | *V.d* | Petiole |
|  |  |  | CMRP4322 | *V.d* | Leaf |
|  |  |  | CMRP4326 | *S.a* | Leaf |
|  |  |  | CMRP4332 | *V.d* | Leaf |
|  |  | *D. cerradensis* | CMRP4331 | *S.a* | Leaf |
|  |  |  | CMRP4324 | *S.a* | Leaf |
|  |  | *D.* cf. *heveae* 1 | CMRP4329 | *S.a* | Petiole |
|  |  | *Diaporthe* sp. | CMRP4330 | *V.d* | Petiole |
|  | Coniochaetales | *Coniochaeta* sp. | CMRP4325 | *V.d* | Petiole |
|  | Xylariales | *Nemania primolutea* | CMRP4323 | *S.a* | Leaf |
| Dothideomycetes | Botryosphaeriales | *Pseudofusicoccum stromaticum* | CMRP4328 | *S.a* | Leaf |
| Eurotiomycetes | Eurotiales | *Aspergillus* sp. section *Flavi* | CMRP4327 | *S.a* | Leaf |


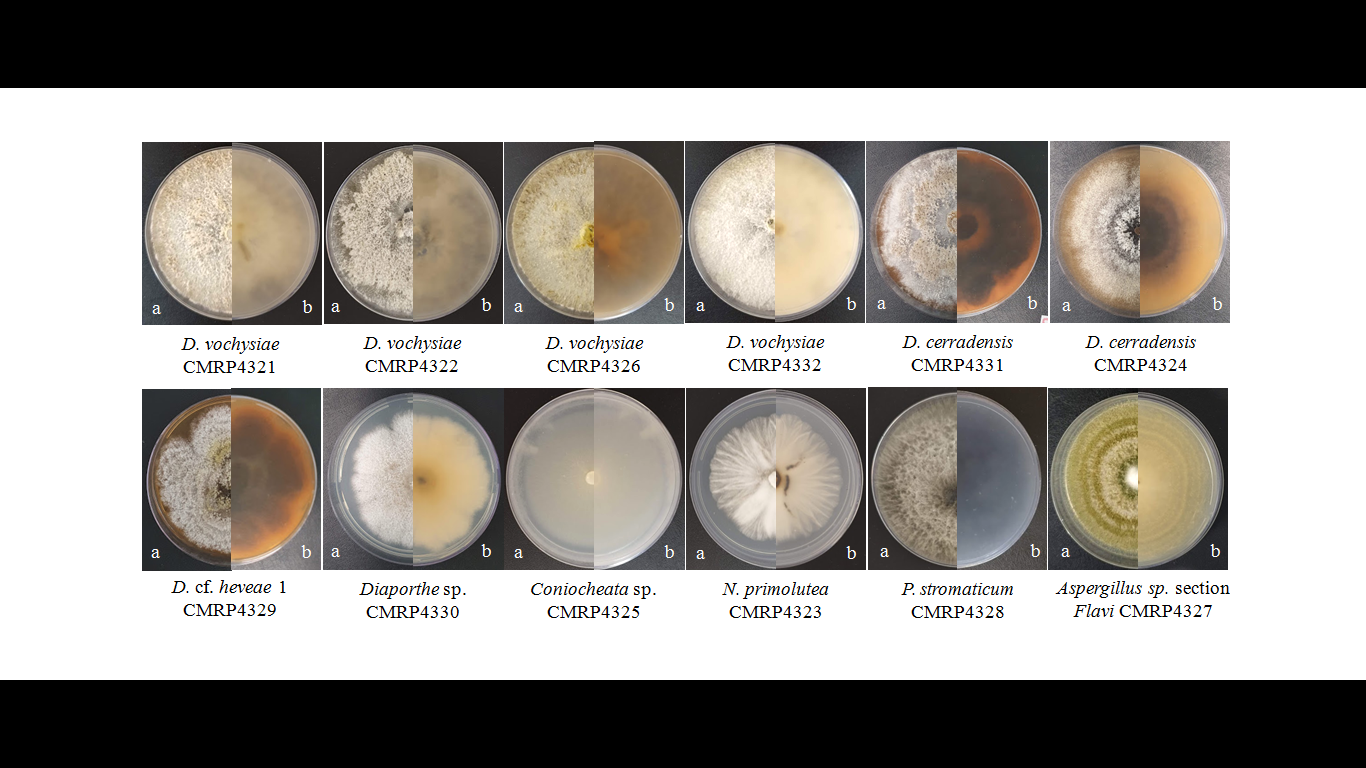


Figure S1. Front (a) and back view (b), respectively of colonies on PDA of twelve endophytic fungi used in this study isolated from the medicinal plants *Stryphnodendron adstringens* and *Vochysia divergens*.


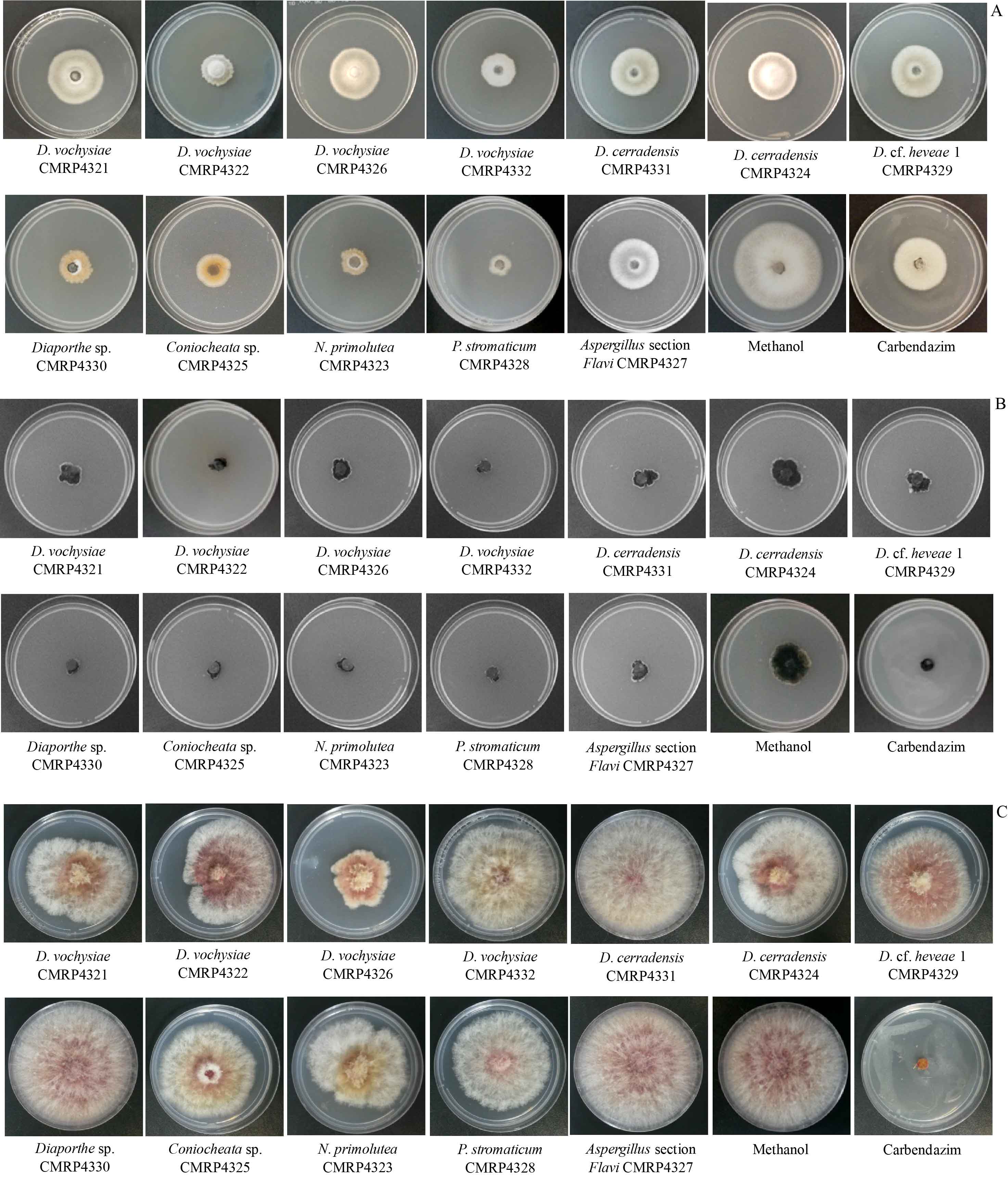


Figure S2. Antifungal activity of extracts of the isolates representing the twelve phenotypes against the mycelial growth of the pathogens *Colletotrichum abscissum* (A), *Phyllosticta citricarpa* (B) and *Fusarium graminearum* (C).

Table S4. Collection details and GenBank accession numbers of isolates of *Aspergillus* section *Flavi*, *Coniocheata*, *Nemania* and *Pseudofusicoccum* genus included in this study.

|  | **Collection number^1^** |  |  | **GenBank accession no.^2^** | |
| --- | --- | --- | --- | --- | --- |
| **Species** |  | **Host/source** | **Country** | **ITS** | ***tef1*** |
| *Aspergillus aflatoxiformans* | DTO 228-G2* | Agricultural soils | Nigeria | MG662388 | NE |
|  | DTO 087-A2 | Soil near road | Madagascar | MG662405 | NE |
| *A. alliaceus* | CBS 536.65* | - | USA | MH858707 | NE |
|  | DTO 368-C3 | soil | Turkey | MH279439 | NE |
| *A. arachidicola* | CBS 117610* | Peanut leaf | Argentina | MF668184 | NE |
|  | DTO 228-H9 | *Protea roupelliae* var. *roupelliae* | South Africa | MG662384 | NE |
| *A. aspearensis* | CBS 143672* | soil | Iran | MG662398 | NE |
|  | DTO 203-D4 | soil | Iran | MG662399 | NE |
| *A. austwickii* | CBS 143677* | stored rice grains from market | Nigeria | MG662391 | NE |
|  | DTO 228-G8 | Sesame kernels from market | Nigeria | MG662386 | NE |
| *A. avenaceus* | CBS 109.46* | - | Japan | AF104446 | NE |
| *A. bertholletius* | CCT 7615* | - | - | JX198673 | NE |
| *A. bombycis* | CBS 117187* | - | Japan | AF104444 | NE |
| *A. caelatus* | CBS 763.97* | - | USA | MH862672 | NE |
|  | NRRL 26100 | - | - | EF661550 | NE |
|  | CBS 143674* | stored rice grains from market | Nigeria | MG662394 | NE |
|  | DTO 228-E9 | stored rice grains from market | Nigeria | MG662393 | NE |
| *A. coremiiformis* | CBS 553.77* | - | - | AJ874114 | NE |
| *A. flavus* | CBS 100927* | - | - | KJ175414 | NE |
|  | NRRL 458 | - | - | EF661562 | NE |
|  | CBS 143688 | - | - | EF661563 | NE |
| *A. lanosus* | CBS 650.74* | - | India | FJ491471 | NE |
| *A. leporis* | CBS 151.66* | dung of *Lepus townsensii* | USA | MH279391 | NE |
|  | [CBS 125914](http://www.cbs.knaw.nl/collections/BioloMICS.aspx?Fields=All&ExactMatch=T&Table=CBS+strain+database&Name=CBS+125914) | A1 horizon soil | USA | MH279389 | NE |
| *A. luteovirescens* | CBS 620.95* | - | - | MG662406 | NE |
| *A. minisclerotigenes* | CBS 117635* | - | - | EF409239 | NE |
|  | DTO 009-F5 | - | - | MG662408 | NE |
| *A. mottae* | CBS 130016* | - | - | JF412767 | NE |
| *A. muricatus* | NRRL 35674* | - | - | [EF661434](https://www.ncbi.nlm.nih.gov/nuccore/EF661434) | NE |
| *A. neoalliaceus* | CBS 134375 | soil | Czech Republic | MH279441 | NE |
|  | CBS 143681* | soil | Czech Republic | MH279420 | NE |
| *A. nomius* | NRRL 13137* | - | - | AF027860 | NE |
|  | DTO 161-F1 | bamboo sample | Thailand | MH279387 | NE |
| *A. novoparasiticus* | CBS 126849* | sputum of a leukemic patient | Brazil | MG662397 | NE |
|  | DTO 223-C5 | air sample | Brazil | MH279415 | NE |
| *A. oryzae* | NRRL 447* | - | - | [EF661560](https://www.ncbi.nlm.nih.gov/nuccore/EF661560) | NE |
| *A. parasiticus* | CBS 100926* | - | - | KJ175437 | NE |
|  | DTO 046-C2 | - | - | MG662400 | NE |
| *A. pipericola* | CBS 143680* | Black pepper | Denmark | MG662385 | NE |
| *A. pseudocaelatus* | CBS 117616* | - | - | KY937935 | NE |
| *A. pseudonomius* | DTO 267-D6 | house dust | Micronesia | MH279416 | NE |
|  | NRRL 3353 | - | - | AF338643 | NE |
| *A. sergii* | CBS 130017* | - | - | JF412769 | NE |
| *A. sojae* | CBS 100928 | - | - | KJ175434 | NE |
|  | NRRL 1988 | - | - | EF661546 | NE |
| ***Aspergillus sp.*** | **CMRP4327** | ***Stryphnodendron adstringens*** | **Brazil** | [**MN173202**](https://www.ncbi.nlm.nih.gov/nuccore/MN173193) | **NE** |
| *A. subflavus* | CBS 143683* | soil | Romania | MH279429 | NE |
|  | S843b | moonmilk | Czech Republic | MH279449 | NE |
| *A. tamarii* | DTO 213-H5 | - | - | MG662403 | NE |
|  | CBS 104.13* | - | - | AF004929 | NE |
| *A. togoensis* | CBS 272.89 | - | - | AJ874113 | NE |
| *A. transmontanensis* | CBS 130015* | - | - | JF412774 | NE |
|  | CS13 | - | - | JF412772 | NE |
| *A. vandermerwei* | DTO 199-A9 | soil | USA | MH279390 | NE |
|  | NRRL 5108* | - | - | EF661567 | NE |
| *Coniochaeta acaciae* | MFLUCC 17-2298* | *Acacia* sp. | Uzbekistan | MG062735 | NE |
| *C. africana* | CBS 120868* | *Prunus salicina* | South Africa | GQ154539 | NE |
| *C. angustispora* | CBS 144.70* | - | Netherlands | MH859528 | NE |
|  | CBS 871.73 | - | Suriname | MH860816 | NE |
| *C. baysunika* | TASM 6131* | *Rosa* sp. | Uzbekistan | MG828880 | NE |
| *C. boothii* | CBS 381.74* | - | USA | MH860862 | NE |
| *C. canina* | UTHSC112460* | canine breed German Shepard | - | [JX481775](https://www.ncbi.nlm.nih.gov/nuccore/JX481775) | NE |
| *C. cateniformis* | UTHSC011644* | canine bone marrow | - | [HE610331](https://www.ncbi.nlm.nih.gov/nuccore/HE610331) | NE |
| *C. cephalothecoides* | L821 | *Trametes cinnabarina* | China | KY064029 | NE |
| *C. cipronana* | CBS 144016* | 19th century art lamina | Costa Rica | [MF422164](https://www.ncbi.nlm.nih.gov/nuccore/MF422164) | NE |
| *C. coluteae* | MFLUCC 17-2299* | *Colutea paulsenii* | Uzbekistan | MG137251 | NE |
| *C. cymbiformispora* | NBRC 32199* | swamp soi | Japan | LC146726 | NE |
| *C. decumbens* | CBS 153.42* | fruit | - | HE610337 | NE |
| *C. discospora* | CBS 168.58* | - | Canada | MH857740 | NE |
| *C. ellipsoidea* | CBS 137.68* | - | Japan | MH859091 | NE |
| *C. ershadii* | CBS 119785* | *Pistacia vera* | Iran | GU553328 | NE |
| *C. euphorbiae* | CBS 139768* | - | Iran | KP941076 | NE |
| *C. extramundana* | CBS 247.77* | - | USA | MH861057 | NE |
| *C. fasciculata* | CBS 205.38* | butter | - | HE610336 | NE |
| *C. fodinicola* | CBS 136963* | uranium mine process waters | Australia | JQ904603 | NE |
|  | CBS 136964 | uranium mine process waters | Australia | JQ904604 | NE |
| *C. gigantospora* | ILLS 60816* | - | France | NR 121521 | NE |
| *C. hoffmannii* | CBS 245.38* | butter | - | HE610332 | NE |
|  | CBS 997.68 | - | Austria | MH859265 | NE |
| *C. iranica* | CBS 139767* | - | Iran | KP941078 | NE |
| *C. lignicola* | CBS 267.33* | - | - | HE610335 | NE |
|  | CBS 127652 | - | USA | MH864654 | NE |
| *C. luteorubra* | CBS131710* | - | USA | MH865901 | NE |
| *C. luteoviridis* | CBS 206.38* | butter | - | HE610333 | NE |
| *C. mutabilis* | CBS 157.44* | river water | - | HE610334 | NE |
| *C. navarrae* | LTA3 CBS141016* | *Ulmus* sp. | Spain | [KU762326](https://www.ncbi.nlm.nih.gov/nuccore/KU762326) | NE |
| *C. nepalica* | NBRC 30584* | soil | Nepal | LC146727 | NE |
| *C. ostrea* | CBS 507.70* | - | USA | MH859821 | NE |
| *C. polymorpha* | CBS 132722* | endotracheal secretion | Kuwait | HE863327 | NE |
| *C. prunicola* | CBS 120875* | *Prunus armeniaca* | South Africa | GQ154540 | NE |
|  | CBS 121445 | *Prunus salicina* | South Africa | GQ154541 | NE |
| *C. rosae* | MFLUCC 17-0810 | *Rosa hissarica* | Uzbekistan | MG828883 | NE |
| *C. simbalensis* | NFCCI 4236* | soil | India | MG825743 | NE |
| *Coniochaeta* sp. | LGMF1575 | *Vochysia divergens* | Brazil | MG976384 | NE |
| ***Coniochaeta* sp.** | **CMRP4325** | ***Vochysia divergens*** | **Brazil** | **MN173203** | **NE** |
| *C. taeniospora* | CBS 141014* | *Quercus petraea* | Austria | KU762324 | NE |
|  | CBS 141015 | *Quercus petraea* | Austria | KU762325 | NE |
| *C. velutina* | CBS 120874* | *Prunus salicina* | South Africa | GQ154542 | NE |
|  | CBS 121444 | *Prunus armeniaca* | South Africa | GQ154544 | NE |
| *Chaetosphaeria garethjonesii* | MFLU 16-1019* | - | Thailand | NR 154840 | NE |
| *Nemania abortiva* | BISH 467* | decayed angiosperm wood | USA | GU292816 | NE |
| *N. aenea* | ATCC 60819 | - | - | AF201704 | NE |
| *N. beaumontii* | HAST 405 | bark | Martinique | GU292819 | NE |
| *N. bipapillata* | HAST 90080610 | bark | Taiwa | GU292818 | NE |
| *N. chestersii* | N23A | - | - | AJ390430 | NE |
| *N. diffusa* | HAST 91020401 | *Castanopsis carlesii* var. *sessilis* | Taiwa | GU292817 | NE |
| *N. illita* | YMJ 236 | wood | USA | EF026122 | NE |
| *N. macrocarpa* | CBS 109567* | *-* | USA | MH862830 | NE |
| *N. maritima* | HAST 89120401* | *Kandelia candel* | Taiwa | GU292822 | NE |
| *N. plumbea* | [HKUCC 10917*](https://www.ncbi.nlm.nih.gov/biocollections?term=HKUCC%5BUnique%20institution%20code%5D) | - | Thailand | DQ641634 | NE |
| *N. pouzarii* | ATCC 2612 | - | - | KC477228 | NE |
| *N. primolutea* | HAST 91102001* | *Artocarpus communis* | Taiwan | EF026121 | NE |
|  | **CMRP49323** | ***Stryphnodendron adstringens*** | **Brazil** | [**MN173201**](https://www.ncbi.nlm.nih.gov/nuccore/MN173193) | **NE** |
| *N. serpens* | HAST 235 | soil | Canada | GU292820 | NE |
|  | N20A | - | - | AJ390431 | NE |
| *Biscogniauxia nummularia* | MUCL 51395 | - | France | KY610382 | NE |
| *Pseudofusicoccum adansoniae* | CBS122055* | *Adansonia gibbosa* | Australia | EF585523 | EF585571 |
| *P. adansoniae* | MUCC726 | *Adansonia gregorii* | Australia | GU199383 | GU199403 |
| *P. ardesiacum* | CBS122062* | *Adansonia gibbosa* | Australia | EU144060 | EU144075 |
|  | CBS 122064 | *Eucalyptus* sp. | Australia | EU144062 | EU144077 |
| *P. artocarpi* | CPC 22796* | *Artocarpus heterophyllus* | Thailand | KM006452 | KM006483 |
| *P. calophylli* | MFLUCC 17-2533* | *Calophyllum inophyllum* | Thailand | [MK347764](https://www.ncbi.nlm.nih.gov/nuccore/MK347764) | MK340877 |
| *P. kimberleyense* | CBS122058* | *Acacia synchronicia* | Australia | EU144057 | EU144072 |
|  | BRIP24082 | *Mangifera indica* | Australia | MH183330 | MH188485 |
| *P. olivaceum* | CBS 124939* | *Pterocarpus angolensis* | South Africa | FJ888459 | FJ888437 |
|  | CMW22637 | *Pterocarpus angolensis* | South Africa | FJ888462 | FJ888438 |
| *P. stromaticum* | CBS 138362* | *Eucalyptus urophylla* | Venezuela | KP872348 | KP872378 |
|  | CBS 117448 | *-* | - | KF766223 | - |
|  | **CMRP4328** | ***S. adstringens*** | **Brazil** | **MN173204** | **MT331613** |
|  | LGMF1122 | *Vochysia divergens* | Brazil | JX559543 | - |
|  | LGMF1118 | *V. divergens* | Brazil | JX559540 | - |
| *P. violaceum* | CBS 124936* | *Pterocarpus angolensis* | South Africa | FJ888474 | FJ888442 |
| *Endomelanconiopsis microspora* | CBS 353.97 | *-* | Papua New Guinea | MH862651 | EU683636 |

^1^Collection – Type strains included in analysis are indicated with *; ATCC = American Type Culture Collection, Mayland, USA; BISH = Bishop Museum, Department of Natural Sciences, Hawaii; BRIP = Australian plant pathogen culture collection, Queensland, Australia; CBS = Culture Collection of the Westerdijk Fungal Biodiversity Institute, Utrecht, The Netherlands; CCT= Colecao de Culturas Tropical, Brazil; CMRP = Microbiological Collections of Paraná Network, Federal University of Paraná, Curitiba, Brazil; CMW = Forestry and Agricultural Biotechnology Institute, University of Pretoria, South Africa; CPC = Culture Collection of Pedro Crous, housed at CBS; DTO = The fungal working collection at Westerdijk Fungal Biodiversity Institute, Utrecht, the Netherlands; HAST = Research Center for Biodiversity, Academia Sinica; HKUCC = The University of Hong Kong Culture Collection; ILLS = Illinois Natural History Survey; LGMF= Culture Collection of Laboratory of Genetics of Microorganisms, Federal University of Paraná, Curitiba, Brazil; MFLUCC = Mae Fah Luang University Culture Collection; MUCL Agro- food & Environmental Fungal Collection; NBRC = NITE Biological Resource Center;; NRRL = Agricultural Research Service Culture Collection, USA; TASM = Uzbek Academy of Sciences, Laboratory of Mycology; UTHSC = University of Texas Health Science Center, USA.

^2^GenBank - ITS*:* internal transcribed spacers and intervening 5.8S nrDNA;*tef1*: translation elongaton factor 1-α;

- : Sequence not available; NE= not evaluated;

In bold the isolates of this study;

Table S5. GenBank megablast result for the first 10 sequences using ITS (internal transcribed spacer) partial sequence from CMRP4331 isolate.

| **Strain** | **Collection number** | **GenBank** | **Query Cover (%)** | **Identities (%)** |
| --- | --- | --- | --- | --- |
| *Diaporthe novem* | CBS 127270 | MH864503 | 100 | 96.49 |
| *Diaporthe novem* | CBS 127271 | MH864504 | 100 | 96.35 |
| *Diaporthe novem* | CBS 127269 | MH864502 | 99 | 96.21 |
| *Diaporthe novem* | BRIP54739a | MH020776 | 98 | 96.29 |
| *Diaporthe miriciae* | BRIP 55662c | KJ197283 | 99 | 95.79 |
| *Diaporthe novem* | BRIP56019a | MH020777 | 97 | 96.28 |
| *Diaporthe* sp. | LGMF1616 | MG976419 | 86 | 100.0 |
| *Diaporthe miriciae* | BRIP 54736j | KJ197282 | 98 | 95.76 |
| *Diaporthe novem* | BRIP57398a | MH020778 | 97 | 96.13 |
| *Diaporthe goulteri* | BRIP 55657a | KJ197290 | 97 | 95.87 |

# Table S6. GenBank megablast result for the first 10 sequences using *tub* (beta-tubulin) partial sequence from CMRP4331 isolate.

| **Strain** | **Collection number** | **GenBank** | **Query Cover (%)** | **Identities (%)** |
| --- | --- | --- | --- | --- |
| *Diaporthe* cf. *mayteni* | UFMGCB4807 | KP189348 | 99 | 99.59 |
| *Diaporthe* sp. | UFMGCB 7722 | MT418936 | 92 | 100.0 |
| *Phomopsis* sp. | MCF-2015 | KP189349 | 91 | 94.92 |
| *Diaporthe* sp. | UFMGCB 7255 | MT407181 | 96 | 93.53 |
| *Diaporthe passiflorae* | ZHKUCC20-0023 | MT409301 | 99 | 92.51 |
| *Diaporthe passiflorae* | ZHKUCC20-0022 | MT409300 | 98 | 92.67 |
| *Diaporthe passiflorae* | ZHKUCC20-0021 | MT409299 | 98 | 92.67 |
| *Diaporthe passiflorae* | ZHKUCC20-0020 | MT409298 | 98 | 92.67 |
| *Diaporthe passiflorae* | ZHKUCC20-0019 | MT409297 | 98 | 92.67 |
| *Diaporthe passiflorae* | ZHKUCC20-0018 | MT409296 | 98 | 92.67 |

# Table S7. GenBank megablast result for the first 10 sequences using *tef1* (translation elongation factor 1-alpha) partial sequence from CMRP4331 isolate.

| **Strain** | **Collection number** | **GenBank** | **Query Cover (%)** | **Identities (%)** |
| --- | --- | --- | --- | --- |
| *Diaporthe* cf. *mayteni* | UFMGCB4807 | KP189356 | 100 | 99.69 |
| *Diaporthe* sp.2 | LGMF932 | KC343930 | 83 | 85.29 |
| *Diaporthe* sp. | URM7973 | MK373015 | 83 | 84.87 |
| *Phomopsis* sp. | cgbsy3 | JQ954663 | 99 | 82.32 |
| *Diaporthe citriasiana* | XFKL-15-2 | MN894427 | 99 | 82.32 |
| *Diaporthe citriasiana* | XFAL-1-1 | MN894425 | 99 | 82.32 |
| *Phomopsis* sp. | pc080227GZ | JQ972716 | 99 | 81.71 |
| *Diaporthe citriasiana* | NFFL-2-41 | MN894426 | 99 | 81.71 |
| *Phomopsis* sp. | END-68 | MK754393 | 90 | 82.67 |
| *Phomopsis* sp. | END-65 | MK754392 | 90 | 82.67 |

Table S8. GenBank megablast result for the first 10 sequences using *his* (histone H3) partial sequence from CMRP4331 isolate.

| **Strain** | **Collection number** | **GenBank** | **Query Cover (%)** | **Identities (%)** |
| --- | --- | --- | --- | --- |
| *Diaporthe* sp.4 | LGMF944 | KC343690 | 100 | 93.19 |
| *Diaporthe malorum* | CAA953 | MT309439 | 100 | 92.96 |
| *Diaporthe malorum* | CAA951 | MT309437 | 100 | 92.96 |
| *Diaporthe* sp.2 | LGMF932 | KC343688 | 100 | 92.52 |
| *Diaporthe schini* | CBS 133181 | KC343675 | 100 | 92.33 |
| *Diaporthe* sp. | CAA740 | KY435650 | 97 | 92.81 |
| *Diaporthe* sp. | CAA734 | KY435648 | 97 | 92.81 |
| *Diaporthe schini* | LGMF910 | KC343676 | 99 | 92.27 |
| *Diaporthe paranensis* | CBS 133184 | KC343655 | 100 | 91.95 |
| *Diaporthe terebinthifolli* | LGMF913 | KC343703 | 100 | 91.88 |

# Table S9. GenBank megablast result for the first 10 sequences using *cal* (calmodulin) partial sequence from CMRP4331 isolate.

| **Strain** | **Collection number** | **GenBank** | **Query Cover (%)** | **Identities (%)** |
| --- | --- | --- | --- | --- |
| *Diaporthe* sp.2 | LGMF932 | KC343446 | 98 | 92.09 |
| *Diaporthe manihotia* | MFLU:19-2826 | MW014358 | 88 | 91.48 |
| *Diaporthe terebinthifolli* | LGMF909 | KC343460 | 88 | 90.93 |
| *Diaporthe* sp.3 | CBS 287.29 | KC343447 | 97 | 88.63 |
| *Diaporthe* sp.4 | LGMF944 | KC343448 | 99 | 88.13 |
| *Diaporthe granjae* | PSCG 489 | MK691202 | 88 | 90.86 |
| *Diaporthe mayteni* | CBS 133185 | KC343381 | 97 | 88.72 |
| *Diaporthe* sp. | CFCC 51986 | KY852499 | 88 | 90.62 |
| *Diaporthe* sp. | CAA752 | KY435661 | 99 | 88.07 |
| *Diaporthe granjae* | CBS 180.91 | KC343354 | 88 | 90.62 |

Figure S3. Bayesian Inference phylogenetic tree of *Diaporthe* species based on multiple alignment of ITS, *tub2*, *tef1*, *his3* and *cal* partial sequences. The data matrix had 60 taxa and 2439 characters. The species *Diaporthe amygdali* (CBS126679) was used as outgroup. Strains marked with a “T” correspond to type sequences. The scale bar of 0.02 represents the number of changes. The sequence of the isolates here studied is presented with its isolation code (CMRP4321, CMRP4322, CMRP4326 and CMRP 4332) highlighted in bold.

Figure S4. Bayesian Inference phylogenetic tree of *Diaporthe* species based on multiple alignment of ITS, *tef1*, *tub2*, *his3 and cal* partial sequences. The data matrix had 29 taxa and 2160 characters. The species *Diaporthe perjuncta* (CBS 114435 T) was used as outgroup. Strains marked with a “T” correspond to type sequences. The scale bar of 0.02 represents the number of changes. The sequence of the isolate here studied is presented with its isolation code (CMRP4329) highlighted in bold.

Figure S5. Bayesian Inference phylogenetic tree of *Diaporthe* species based on multiple alignment of ITS, *tef1*, *tub2*, *his3 and cal* partial sequences. The data matrix had 49 taxa and 2112 characters. The species *Diaporthe caulivora* (CBS 127268 T) was used as outgroup. Strains marked with a “T” correspond to type sequences. The scale bar of 0.03 represents the number of changes. The sequence of the isolate here studied is presented with its isolation code (CMRP4330) highlighted in bold.

Figure S6. Bayesian Inference phylogenetic tree of *Aspergillus* section *Flavi* species based on alignment of ITS partial sequences. The data matrix had 55 taxa and *Aspergillus muricatus* (NRRL 35674) was used as outgroup. Strains marked with a “T” correspond to type sequences. The scale bar of 0.02 represents the number of changes. The sequence of the isolate here studied is presented with its isolation code (CMRP4327) highlighted in bold.

Figure S7. Bayesian Inference phylogenetic tree of *Coniocheata* genus based on alignment of ITS partial sequences. The data matrix had 45 taxa and *Cheatosphaeria garethjonessi* (MFLU161019) was used as outgroup. Strains marked with a “T” correspond to type sequences. The scale bar of 0.05 represents the number of changes. The sequence of the isolate here studied is presented with its isolation code (CMRP4325) highlighted in bold.

Figure S8. Bayesian Inference phylogenetic tree of *Nemania* genus based on alignment of ITS partial sequences. The data matrix had 16 taxa and *Biscogniauxia nummularia* (MUCL51395) was used as outgroup. Strains marked with a “T” correspond to type sequences. The scale bar of 0.06 represents the number of changes. The sequence of the isolate here studied is presented with its isolation code (CMRP4323) highlighted in bold.

Figure S9. Bayesian Inference phylogenetic tree of *Pseudofusicoccum* genus based on alignment of *tef1* and ITS partial sequences. The data matrix had 16 taxa and *Endomelanconiopsis microspora* (CBS353.97) was used as outgroup. Strains marked with a “T” correspond to type sequences. The scale bar of 0.02 represents the number of changes. The sequence of the isolate here studied is presented with its isolation code (CMRP4328) highlighted in bold.

.


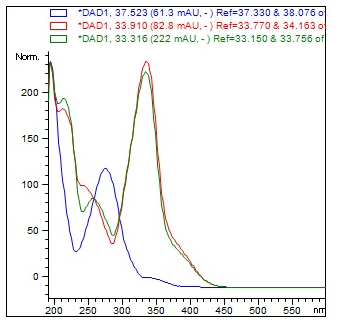

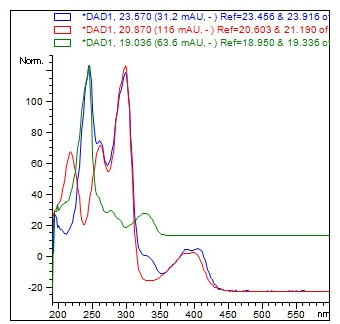

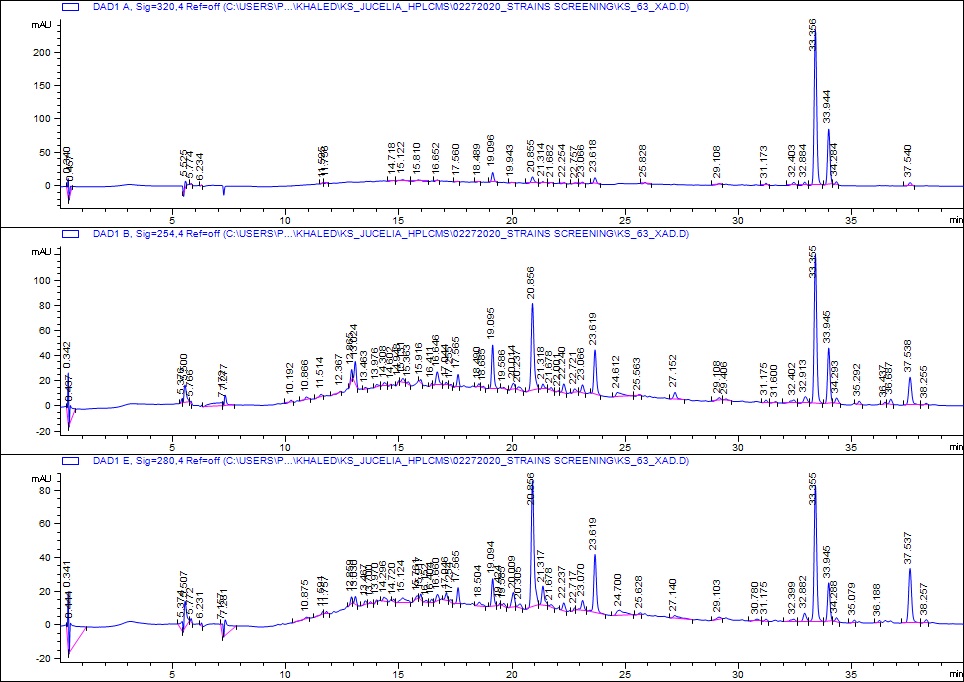


Figure S10. HPLC/UV analyses of the extract produced by *Diaporthe vochysiae* CMRP4321. HPLC-conditions: solvent A: H_2_O/0.1% FA; solvent B: CH_3_CN; flow rate: 0.5 mL min^-1^; 0-30 min, 5-100% B (linear gradient); 30-35 min, 100% B; 35-36 min, 100-5% B (linear gradient); 36-40 min, 5% B; 254 nm. UV-vis inset of full wavelength scan (190-600 nm).

**MW: 503**

**[(M-H_2_O)+H]^+^**

**[(M-H_2_O)+H]^+^**

**[M+H]^+^**

**MW: 690**

**[M+H]^+^**

**MW: 750**

**[M+Na]^+^**

**[M+H]^+^**

Figure S11. (+)-ESI-MS spectra of the compounds detected in the the extract produced by *Diaporthe vochysiae* CMRP4321. LCMS-conditions: solvent A: H_2_O/0.1% FA; solvent B: CH_3_CN; flow rate: 0.5 mL min^-1^; 0-30 min, 5-100% B (linear gradient); 30-35 min, 100% B; 35-36 min, 100-5% B (linear gradient); 36-40 min, 5% B. Note – no clear mass peaks were detected in the (–)-ESI-MS.


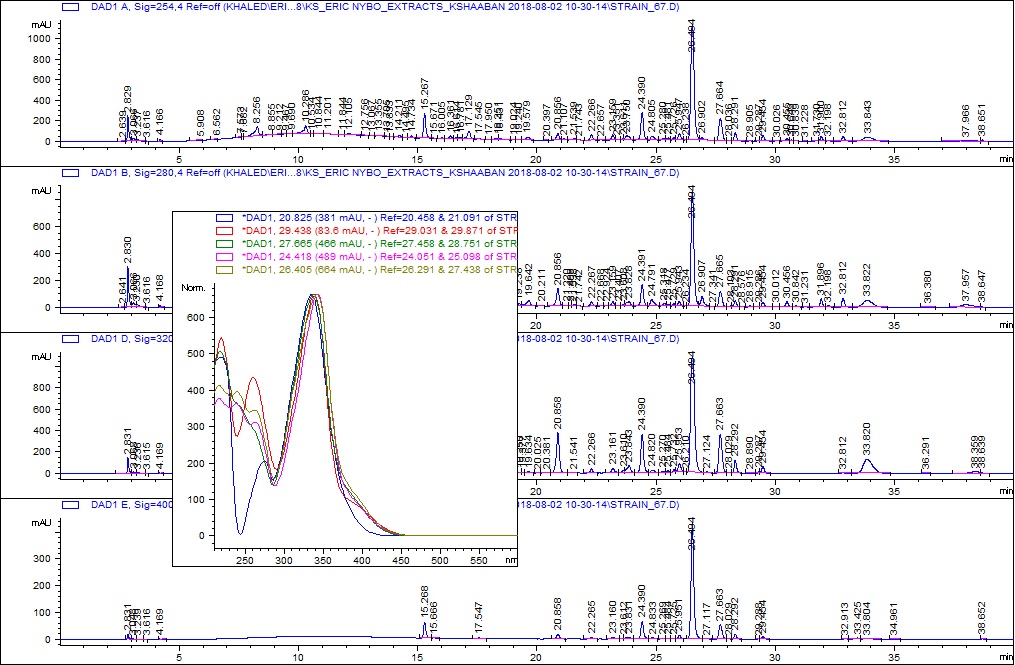


Figure S12. HPLC/UV analyses of the extract produced by *Diaporthe vochysiae* CMRP4322. HPLC-conditions: solvent A: H_2_O/0.1% FA; solvent B: CH_3_CN; flow rate: 0.5 mL min^-1^; 0-30 min, 5-100% B (linear gradient); 30-35 min, 100% B; 35-36 min, 100-5% B (linear gradient); 36-40 min, 5% B; 254 nm, 280 nm, 320 nm, 400 nm.UV-vis in set of full wavelength scan (190-600 nm).


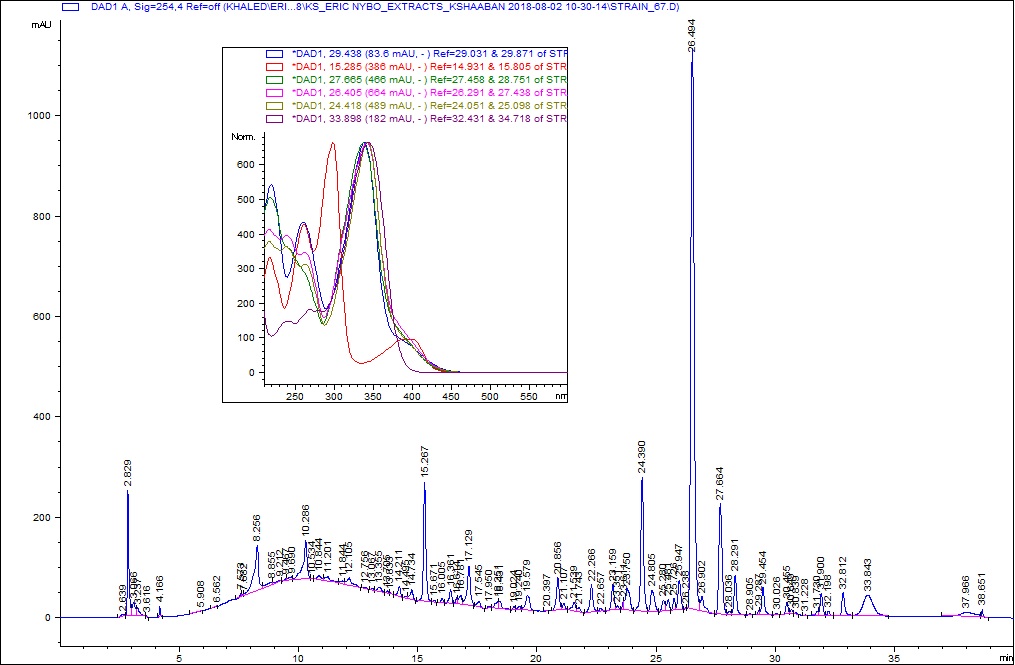


Figure S13. HPLC/UV analyses of the extract produced by *Diaporthe vochysiae* CMRP4322. HPLC-conditions: solvent A: H_2_O/0.1% FA; solvent B: CH_3_CN; flow rate: 0.5 mL min^-1^; 0-30 min, 5-100% B (linear gradient); 30-35 min, 100% B; 35-36 min, 100-5% B (linear gradient); 36-40 min, 5% B; 254 nm. UV-vis in set of full wavelength scan (190-600 nm).


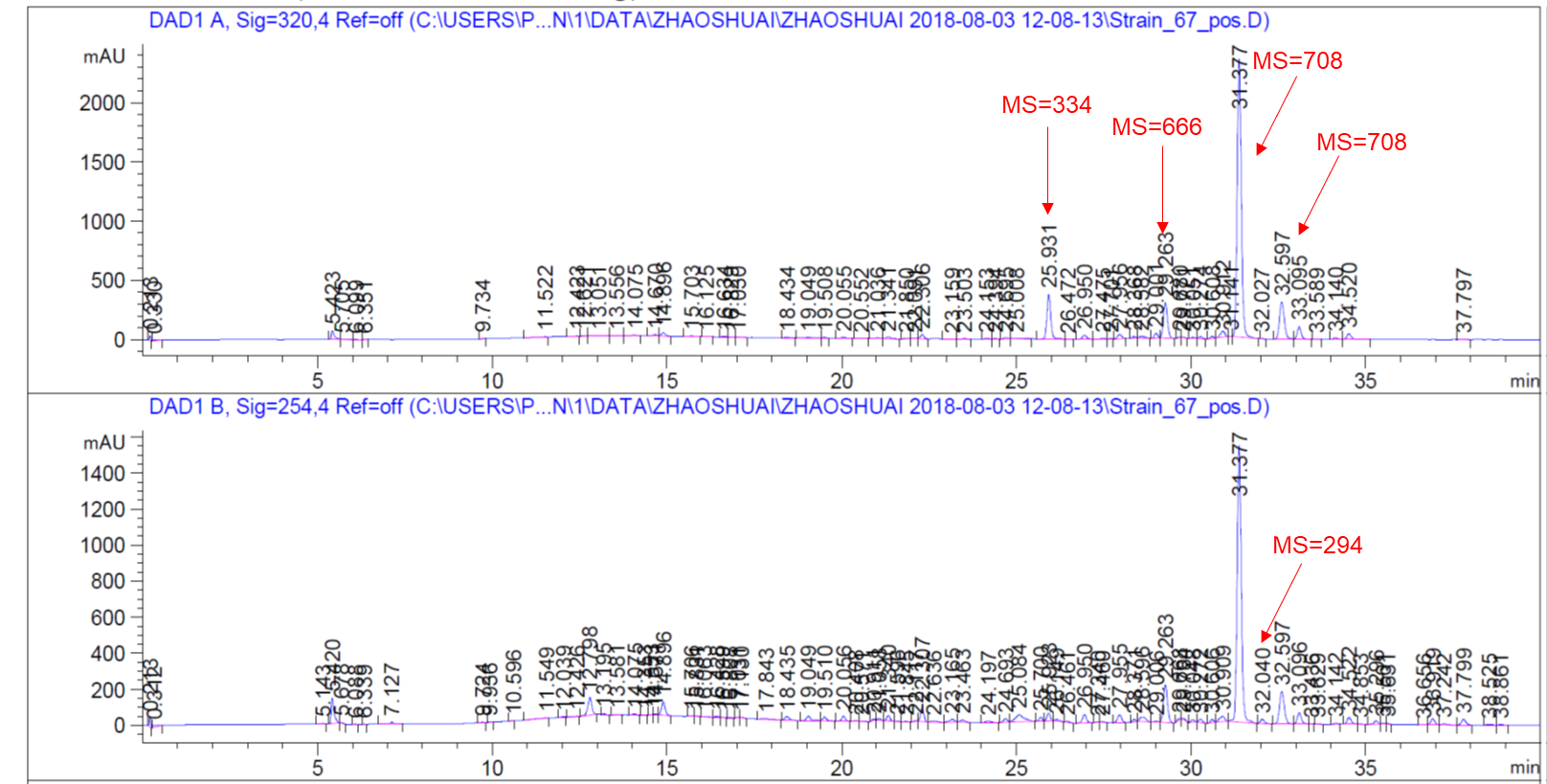


Figure S14. Summary of LCMS analyses of the compounds detected in the extract produced by *Diaporthe vochysiae* CMRP4322. LCMS-conditions: solvent A: H_2_O/0.1% FA; solvent B: CH_3_CN; flow rate: 0.5 mL min^-1^; 0-30 min, 5-100% B (linear gradient); 30-35 min, 100% B; 35-36 min, 100-5% B (linear gradient); 36-40 min, 5% B; 320 nm, 254 nm.

**MW: 666**

**[M+H]^+^**

**[M-H]**^−^

**MW: 708**

**[M-H]**^−^

**[M+H]^+^**

**[M-H]**^−^

**[M+H]^+^**

**MW: 708**

Figure S15. (+) and (–)-ESI-MS spectra of the compounds detected in the the extract produced by *Diaporthe vochysiae* CMRP4322. LCMS-conditions: solvent A: H_2_O/0.1% FA; solvent B: CH_3_CN; flow rate: 0.5 mL min^-1^; 0-30 min, 5-100% B (linear gradient); 30-35 min, 100% B; 35-36 min, 100-5% B (linear gradient); 36-40 min, 5% B.

**MW: 334**

**MW: 294**

**[M+Na]^+^**

**[M-H]**^−^

**[M+H]^+^**

**[M-H]**^−^

**[M+H]^+^**

Figure S16. (+) and (–)-ESI-MS spectra of the compounds detected in the the extract produced by *Diaporthe vochysiae* CMRP4322. LCMS-conditions: solvent A: H_2_O/0.1% FA; solvent B: CH_3_CN; flow rate: 0.5 mL min^-1^; 0-30 min, 5-100% B (linear gradient); 30-35 min, 100% B; 35-36 min, 100-5% B (linear gradient); 36-40 min, 5% B.


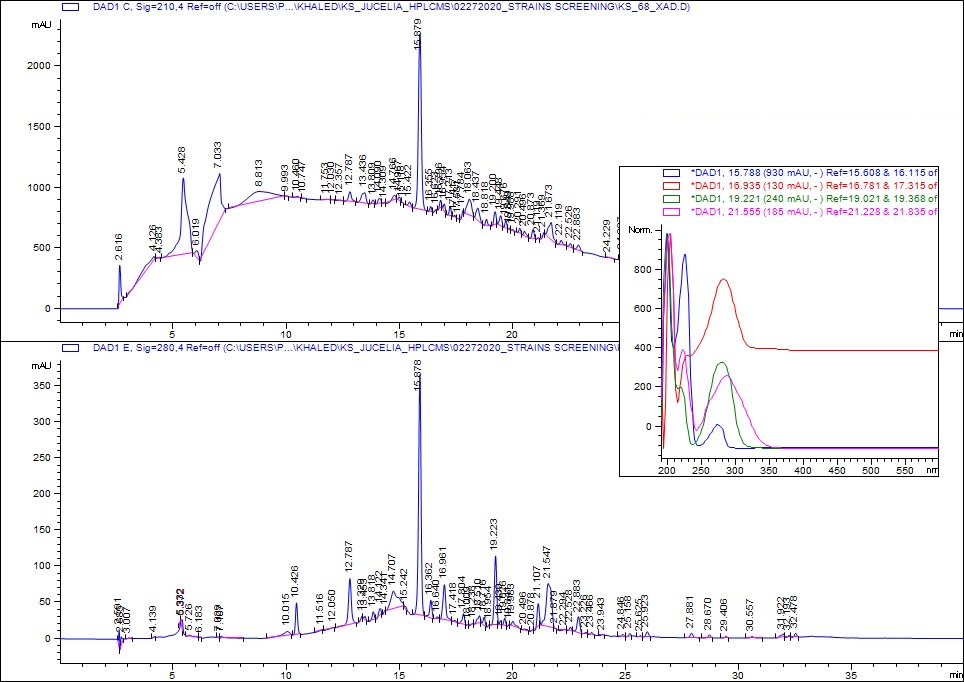


Figure S17. HPLC/UV analyses of the extract produced by *Nemania primolutea* CMRP4323. HPLC-conditions: solvent A: H_2_O/0.1% FA; solvent B: CH_3_CN; flow rate: 0.5 mL min^-1^; 0-30 min, 5-100% B (linear gradient); 30-35 min, 100% B; 35-36 min, 100-5% B (linear gradient); 36-40 min, 5% B; 210 nm, 254 nm. UV-vis in set of full wavelength scan (190-600 nm).

**MW: 174?**

**MW: 272?**

**[(M-H_2_O)+H]^+^**

**[M+H]^+^**

**[M+H]^+^**

Figure S18. (+) and (–)-ESI-MS spectra of the major compounds detected in the extract produced by *Nemania primolutea* CMRP4323. LCMS-conditions: solvent A: H_2_O/0.1% FA; solvent B: CH_3_CN; flow rate: 0.5 mL min^-1^; 0-30 min, 5-100% B (linear gradient); 30-35 min, 100% B; 35-36 min, 100-5% B (linear gradient); 36-40 min, 5% B.Note – no clear mass peaks were detected in the (–)-ESI-MS.


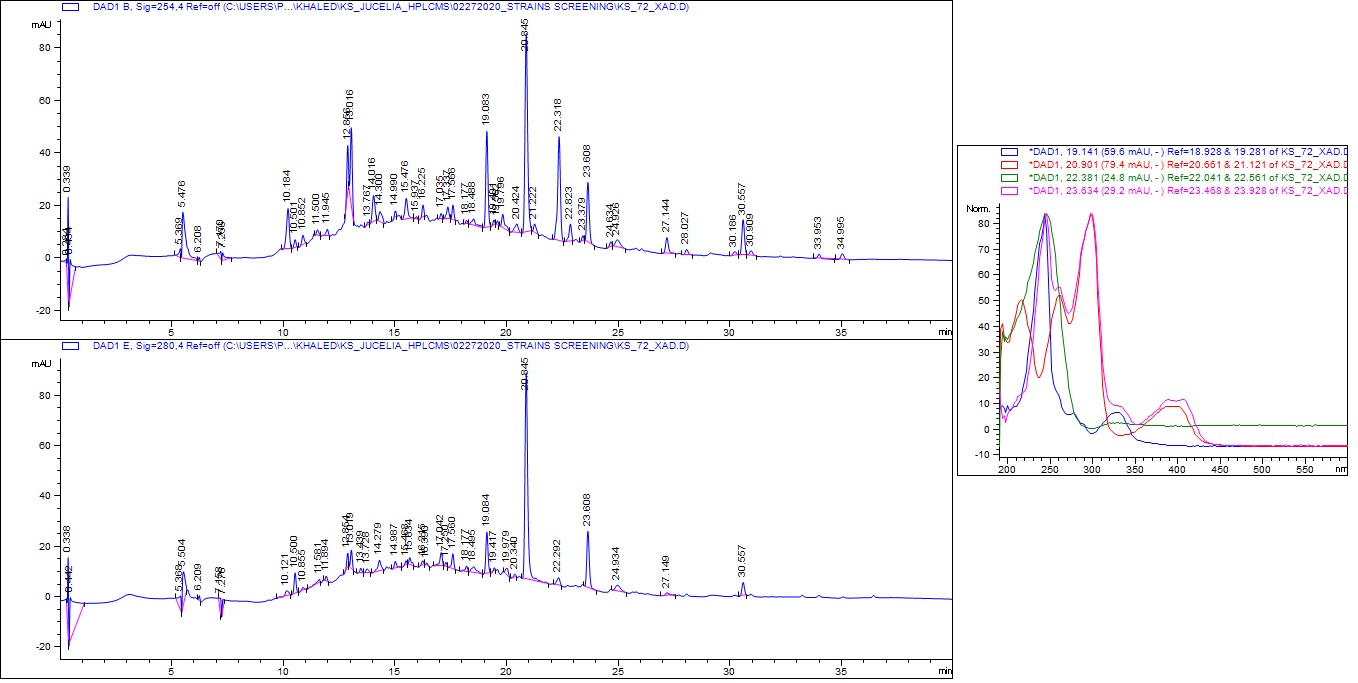


Figure S19. HPLC/UV analyses of the extract produced by *Diaporthe cerradensis*  CMRP4324. HPLC-conditions: solvent A: H_2_O/0.1% FA; solvent B: CH_3_CN; flow rate: 0.5 mL min^-1^; 0-30 min, 5-100% B (linear gradient); 30-35 min, 100% B; 35-36 min, 100-5% B (linear gradient); 36-40 min, 5% B; 254 nm. UV-vis in set of full wavelength scan (190-600 nm).

**[(M-H_2_O)+H]^+^**

**[M-H]**^−^

**[M+H]^+^**

**MW: 396**

Figure S20. (+) and (–)-ESI-MS spectra of the compounds detected in the extract produced by *Diaporthe cerradensis*  CMRP4324. LCMS conditions: solvent A: H_2_O/0.1% FA; solvent B: CH_3_CN; flow rate: 0.5 mL min^-1^; 0-30 min, 5-100% B (linear gradient); 30-35 min, 100% B; 35-36 min, 100-5% B (linear gradient); 36-40 min, 5% B.

Figure S21. (+) and (–)-ESI-MS spectra of the compounds detected in the the extract produced by *Diaporthe cerradensis*  CMRP4324. LCMS conditions: solvent A: H_2_O/0.1% FA; solvent B: CH_3_CN; flow rate: 0.5 mL min^-1^; 0-30 min, 5-100% B (linear gradient); 30-35 min, 100% B; 35-36 min, 100-5% B (linear gradient); 36-40 min, 5% B.


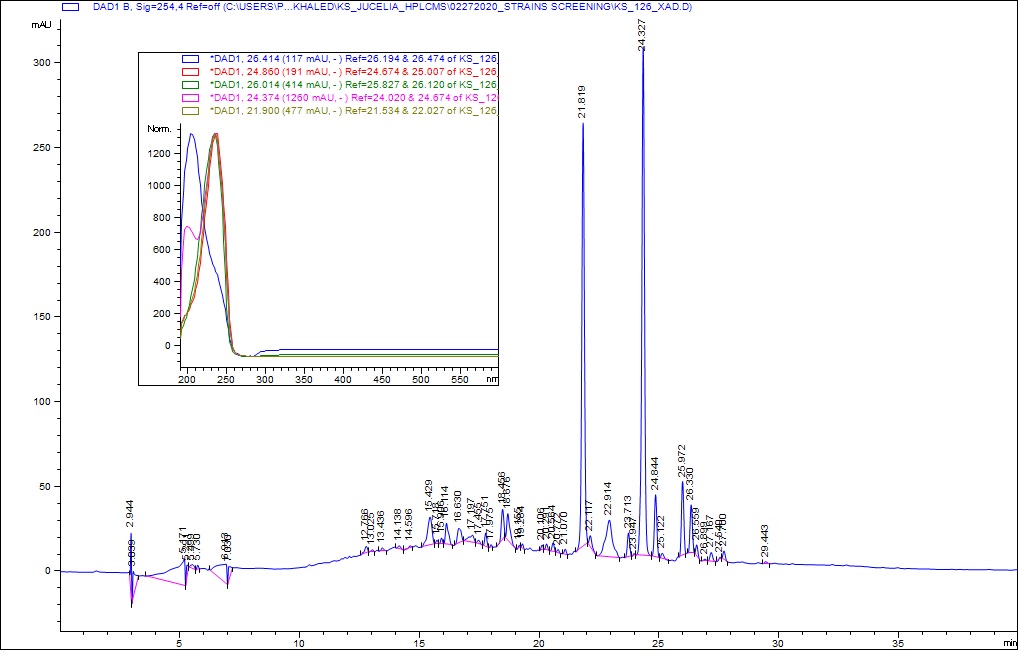


**MW: 306**

**MW: 324**

Figure S22. HPLC/UV analyses of the extract produced by *Coniochaeta* sp. CMRP4325. HPLC-conditions: solvent A: H_2_O/0.1% FA; solvent B: CH_3_CN; flow rate: 0.5 mL min^-1^; 0-30 min, 5-100% B (linear gradient); 30-35 min, 100% B; 35-36 min, 100-5% B (linear gradient); 36-40 min, 5% B; 254 nm. UV-vis inset of full wavelength scan (190-600 nm).

**[M+Na]^+^**

**[(M-H_2_O)+H]^+^**

**[(M-H_2_O)+H]^+^**

**[M+Cl]**^−^

**[M+H]^+^**

**[M-H]**^−^

**MW: 324**

**MW: 306**

Figure S23. (+) and (–)-ESI-MS spectra of the major compounds detected in the extract produced by *Coniochaeta* sp. CMRP4325. LCMS conditions: solvent A: H_2_O/0.1% FA; solvent B: CH_3_CN; flow rate: 0.5 mL min^-1^; 0-30 min, 5-100% B (linear gradient); 30-35 min, 100% B; 35-36 min, 100-5% B (linear gradient); 36-40 min, 5% B.


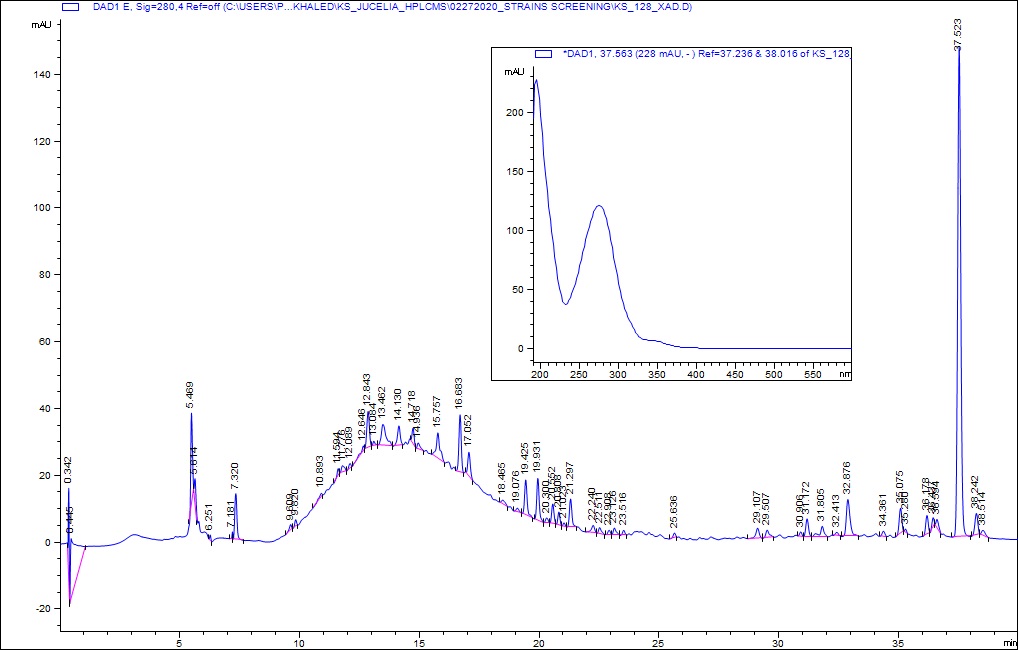


Figure S24. HPLC/UV analyses of the extract produced by *Diaporthe vochysiae* CMRP4326. HPLC-conditions: solvent A: H_2_O/0.1% FA; solvent B: CH_3_CN; flow rate: 0.5 mL min^-1^; 0-30 min, 5-100% B (linear gradient); 30-35 min, 100% B; 35-36 min, 100-5% B (linear gradient); 36-40 min, 5% B; 254 nm. UV-vis in set of full wavelength scan (190-600 nm).

**MW: 414**

**[M+Na]^+^**

**[M+H]^+^**

**MW: 503**

**[M+H]^+^**

**[(M-H_2_O)+H]^+^**

Figure S25. (+) and (–)-ESI-MS spectra of the major compounds detected in the extract produced by *Diaporthe vochysiae* CMRP4326. LCMS conditions: solvent A: H_2_O/0.1% FA; solvent B: CH_3_CN; flow rate: 0.5 mL min^-1^; 0-30 min, 5-100% B (linear gradient); 30-35 min, 100% B; 35-36 min, 100-5% B (linear gradient); 36-40 min, 5% B. Note – no clear mass was detected for the major peak at *R*_t_=37.52 min.


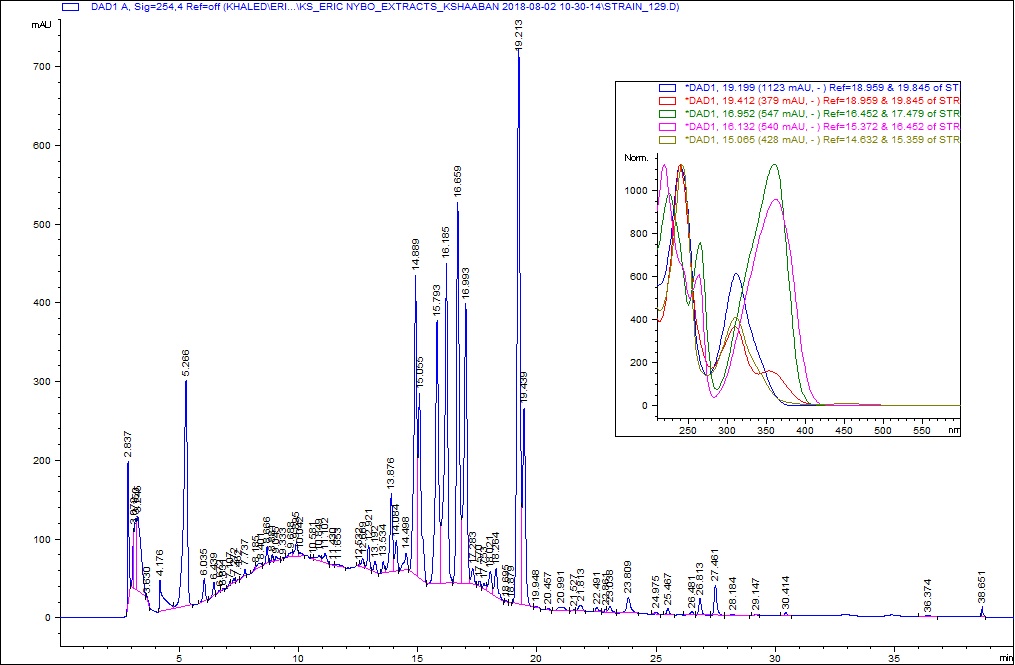


Figure S26. HPLC/UV analyses of the extract produced by *Aspergillus* sp. section *Flavi* CMRP4327. HPLC-conditions: solvent A: H_2_O/0.1% FA; solvent B: CH_3_CN; flow rate: 0.5 mL min^-1^; 0-30 min, 5-100% B (linear gradient); 30-35 min, 100% B; 35-36 min, 100-5% B (linear gradient); 36-40 min, 5% B; 254 nm. UV-vis inset of full wavelength scan (190-600 nm).


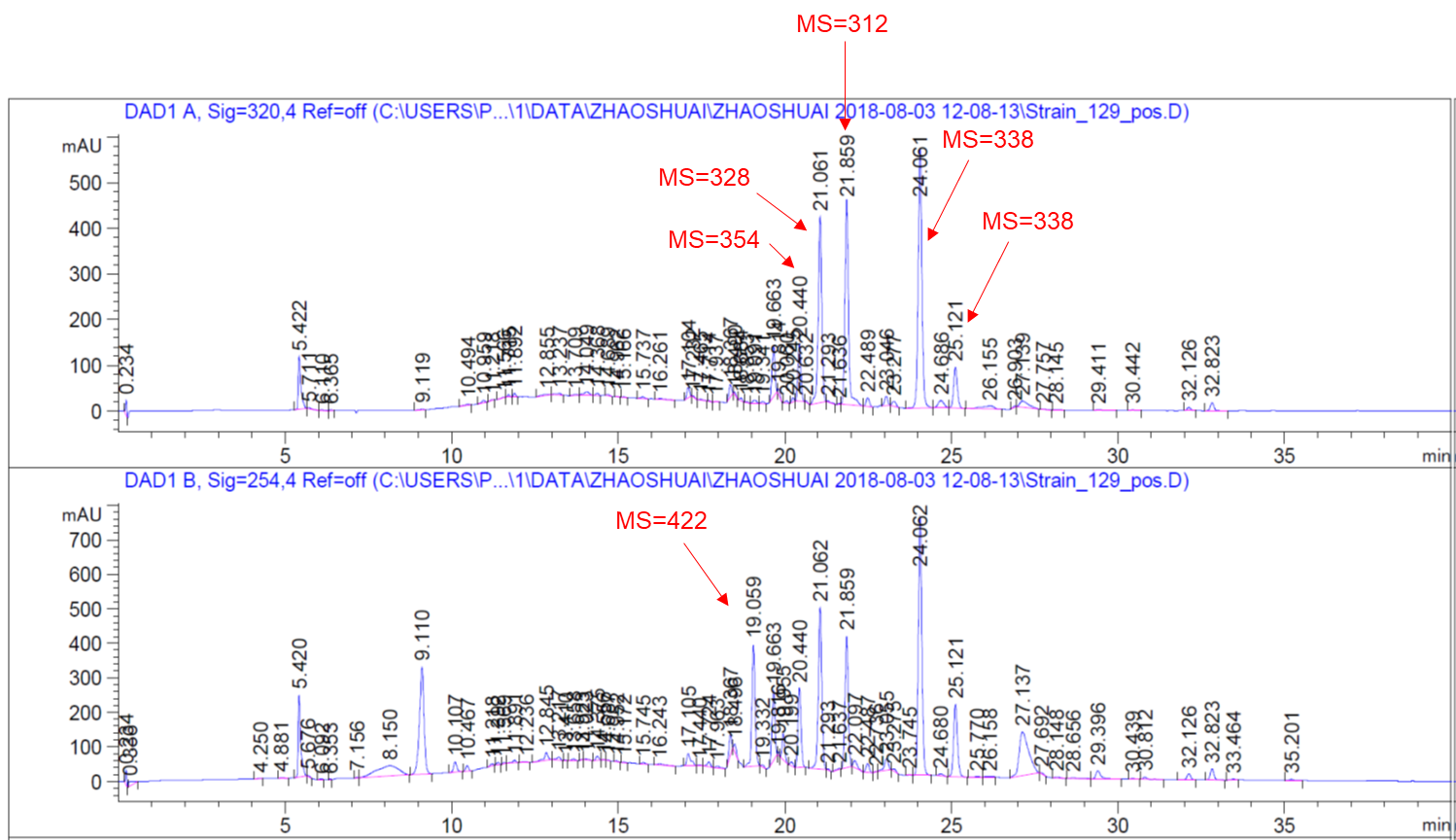


Figure S27. Summary of LCMS analysesof the compounds detected in the extract produced by *Aspergillus* sp. section *Flavi* CMRP4327. LC-MS conditions: H_2_O/0.1% formic acid (solvent A), CH_3_CN/0.1% formic acid (solvent B); flow rate: 0.5 mL min^-1^; 0-2 min, 5% B; 2-30 min, 5-100% B; 30-35 min, 100% B; 35-36 min, 100-5% B; 36-40 min, 5% B.

**[2M+Na]^+^**

**MW: 328**

**[M-H]**^−^

**[M+H]^+^**

**[M+H]^+^**

**[M-H]**^−^

**MW: 312**

**[M-H]**^−^

**MW: 338**

**[M+H]^+^**

Figure S28. (+) and (–)-ESI-MS spectra of the compounds detected in the extract produced by *Aspergillus* sp. section *Flavi* CMRP4327. LCMS conditions: solvent A: H_2_O/0.1% FA; solvent B: CH_3_CN; flow rate: 0.5 mL min^-1^; 0-30 min, 5-100% B (linear gradient); 30-35 min, 100% B; 35-36 min, 100-5% B (linear gradient); 36-40 min, 5% B.

**[2M-H]**^−^

**[M+HCOO]**^−^

**MW: 422**

**MW: 338**

**[2M+Na]^+^**

**[M+Cl]**^−^

**[M+H]^+^**

**[M-H]**^−^

**MW: 354**

**[M+H]^+^**

**[M+H]^+^**

Figure S29. (+) and (–)-ESI-MS spectra of the compounds detected in the the extract produced by *Aspergillus* sp. section *Flavi* CMRP4327.LCMS conditions: solvent A: H_2_O/0.1% FA; solvent B: CH_3_CN; flow rate: 0.5 mL min^-1^; 0-30 min, 5-100% B (linear gradient); 30-35 min, 100% B; 35-36 min, 100-5% B (linear gradient); 36-40 min, 5% B.


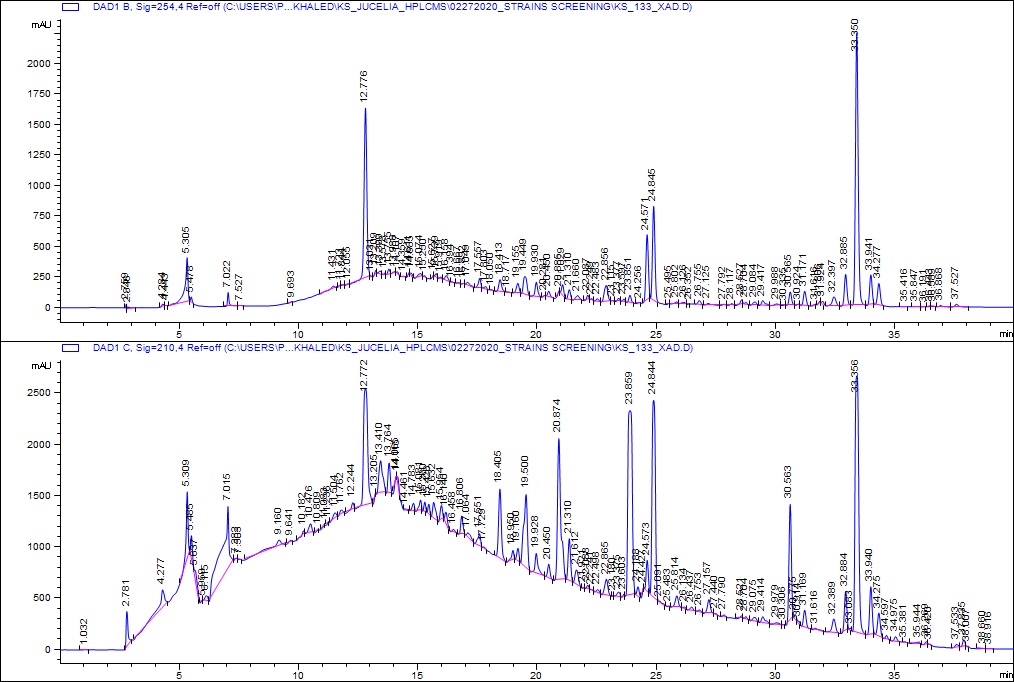

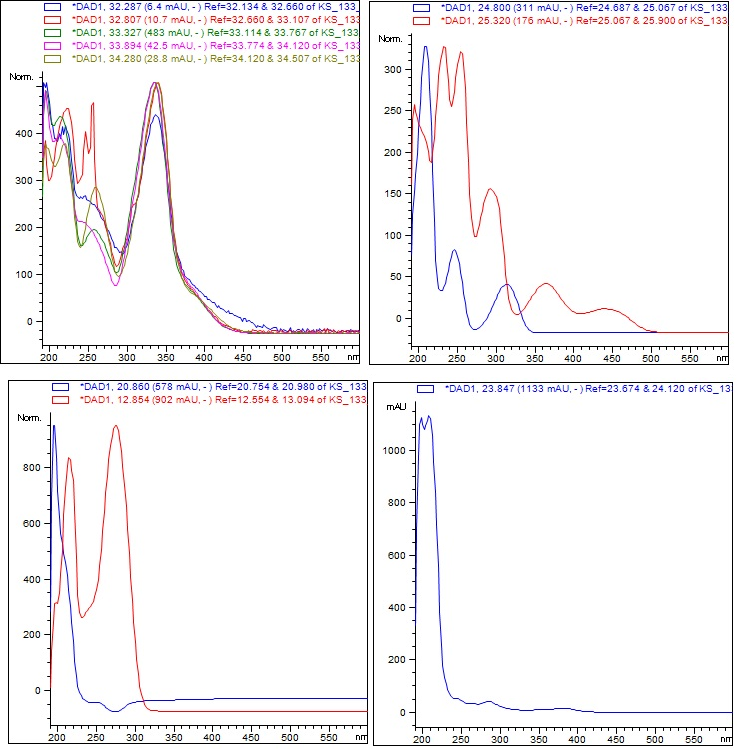


Figure S30. HPLC/UV analyses of the extract produced by *Pseudofusicoccum stromaticum* CMRP4328. HPLC-conditions: solvent A: H_2_O/0.1% FA; solvent B: CH_3_CN; flow rate: 0.5 mL min^-1^; 0-30 min, 5-100% B (linear gradient); 30-35 min, 100% B; 35-36 min, 100-5% B (linear gradient); 36-40 min, 5% B; 210 nm and 254 nm. UV-vis inset of full wavelength scan (190-600 nm).

**MW: 270**

**[M-H]**^−^

**[M+H]^+^**

Figure S31. (+) and (–)-ESI-MS spectra of the compounds detected in the the extract produced by *Pseudofusicoccum stromaticum* CMRP4328. LCMS conditions: solvent A: H_2_O/0.1% FA; solvent B: CH_3_CN; flow rate: 0.5 mL min^-1^; 0-30 min, 5-100% B (linear gradient); 30-35 min, 100% B; 35-36 min, 100-5% B (linear gradient); 36-40 min, 5% B. Note – no clear mass was detected for the major peaks at *R*_t_=30.5 – 34.2 min.


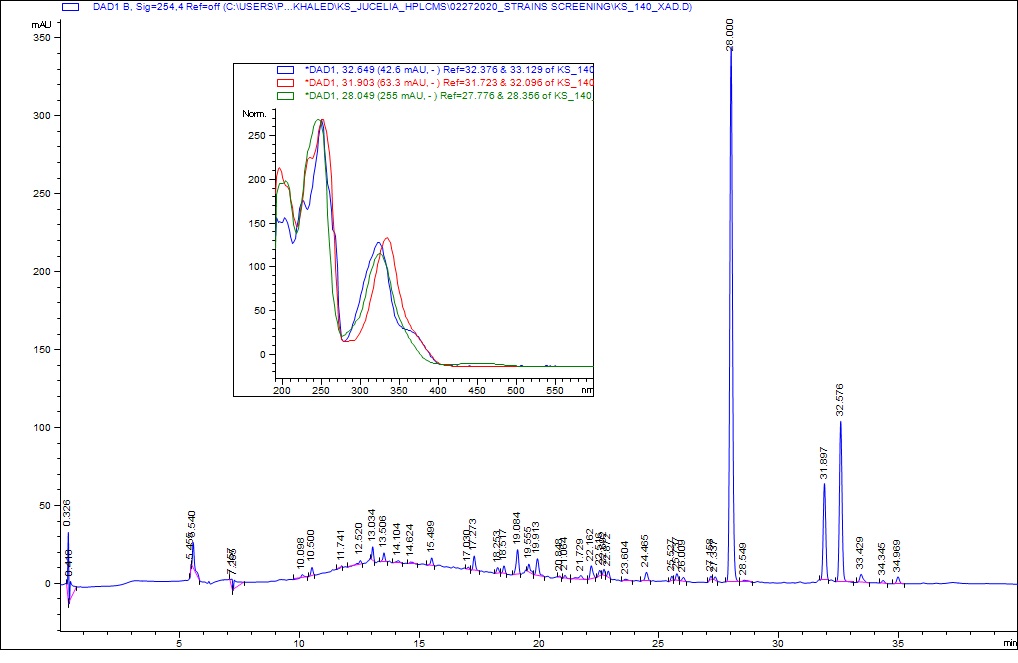


Figure S32. HPLC/UV analyses of the extract produced by *Diaporthe* cf. *heveae* 1 CMRP4329. HPLC-conditions: solvent A: H_2_O/0.1% FA; solvent B: CH_3_CN; flow rate: 0.5 mL min^-1^; 0-30 min, 5-100% B (linear gradient); 30-35 min, 100% B; 35-36 min, 100-5% B (linear gradient); 36-40 min, 5% B; 254 nm. UV-vis inset of full wavelength scan (190-600 nm).

**MW: 324**

**[2M+Na]^+^**

**[M+Na]^+^**

**[M+H]^+^**

Figure S33. (+) and (–)-ESI-MS spectra of the compounds detected in the the extract produced by *Diaporthe* cf. *heveae* 1 CMRP4329. LC-MS conditions: H_2_O/0.1% formic acid (solvent A), CH_3_CN/0.1% formic acid (solvent B); flow rate: 0.5 mL min^-1^; 0-2 min, 5% B; 2-30 min, 5-100% B; 30-35 min, 100% B; 35-36 min, 100%-5% B; 36-40 min, 5% B. Note – no clear mass was detected for the peaks at *R*_t_=31.89 and 32.57 min.


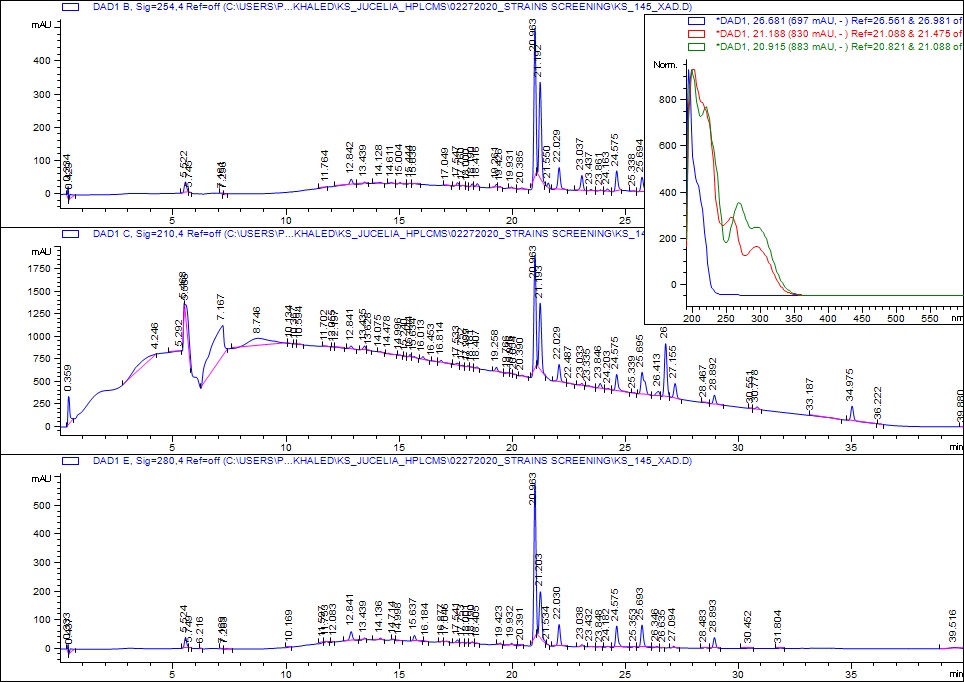


Figure S34. HPLC/UV analyses of the extract produced by *Diaporthe* sp. CMRP4330. HPLC-conditions: solvent A: H_2_O/0.1% FA; solvent B: CH_3_CN; flow rate: 0.5 mL min^-1^; 0-30 min, 5-100% B (linear gradient); 30-35 min, 100% B; 35-36 min, 100%-5% B (linear gradient); 36-40 min, 5% B; 254 nm, 210 nm, 280 nm. UV-vis in set of full wavelength scan (190-600 nm).

**[M-H]**^−^

**MW: 338**

**[M+Na]^+^**

**[M+H]^+^**

**[M+Na]^+^**

**[M+H]^+^**

**MW: 338**

Figure S35. (+) and (–)-ESI-MS spectra of the compounds detected in the extract produced by *Diaporthe* sp. CMRP4330. LC-MS conditions: H_2_O/0.1% formic acid (solvent A), CH_3_CN/0.1% formic acid (solvent B); flow rate: 0.5 mL min^-1^; 0-2 min, 5% B; 2-30 min, 5-100% B; 30-35 min, 100% B; 35-36 min, 100%-5% B; 36-40 min, 5% B.


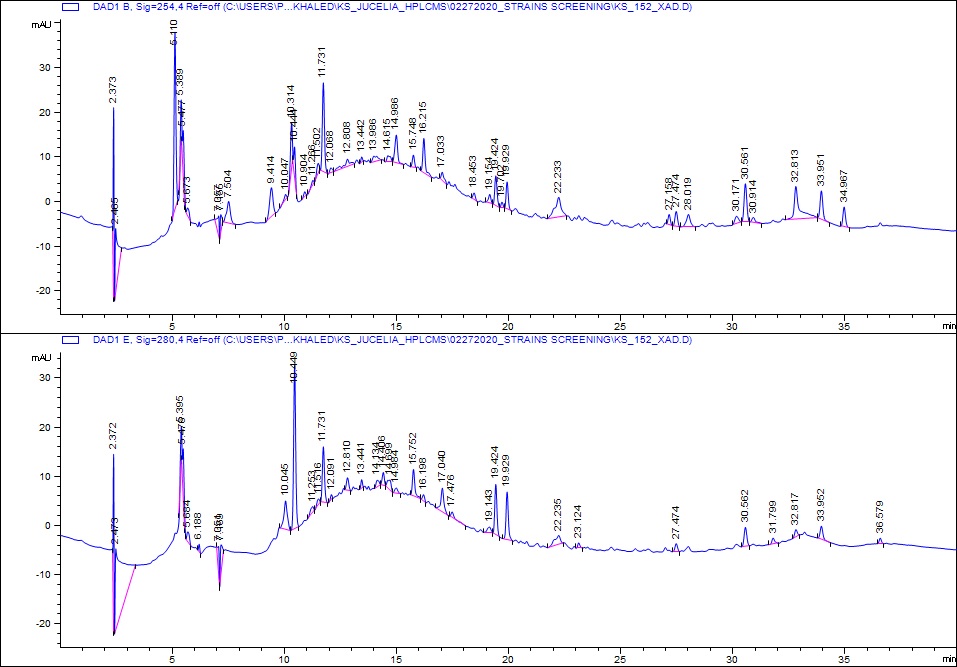


Figure S36. HPLC/UV analyses of the extract produced by *Diaporthe cerradensis* CMRP4331. HPLC-conditions: solvent A: H_2_O/0.1% FA; solvent B: CH_3_CN; flow rate: 0.5 mL min^-1^; 0-30 min, 5-100% B (linear gradient); 30-35 min, 100% B; 35-36 min, 100%-5% B (linear gradient); 36-40 min, 5% B; 254 nm, 280 nm. No major peaks were detected in the HPLC-UV analysis of this fungal extract.

**[M-(132)+H]^+^**

**[M-(132)+H]^+^**

**[M-(88)+H]^+^**

**[M-(44)+H]^+^**

**[M+H]^+^**

**MW: 405?**

**MW: 282**

**[M+H]^+^**

**[M+Na]^+^**

**MW: 414**

**[M+Na]^+^**

**[M+H]^+^**

Figure S37. (+)-ESI-MS spectra of the compounds detected in tthe extract produced by *Diaporthe cerradensis* CMRP4331. LC-MS conditions: H_2_O/0.1% formic acid (solvent A), CH_3_CN/0.1% formic acid (solvent B); flow rate: 0.5 mL min^-1^; 0-2 min, 5% B; 2-30 min, 5-100% B; 30-35 min, 100% B; 35-36 min, 100%-5% B; 36-40 min, 5% B.Note – no clear mass peaks were detected in the (–)-ESI-MS.


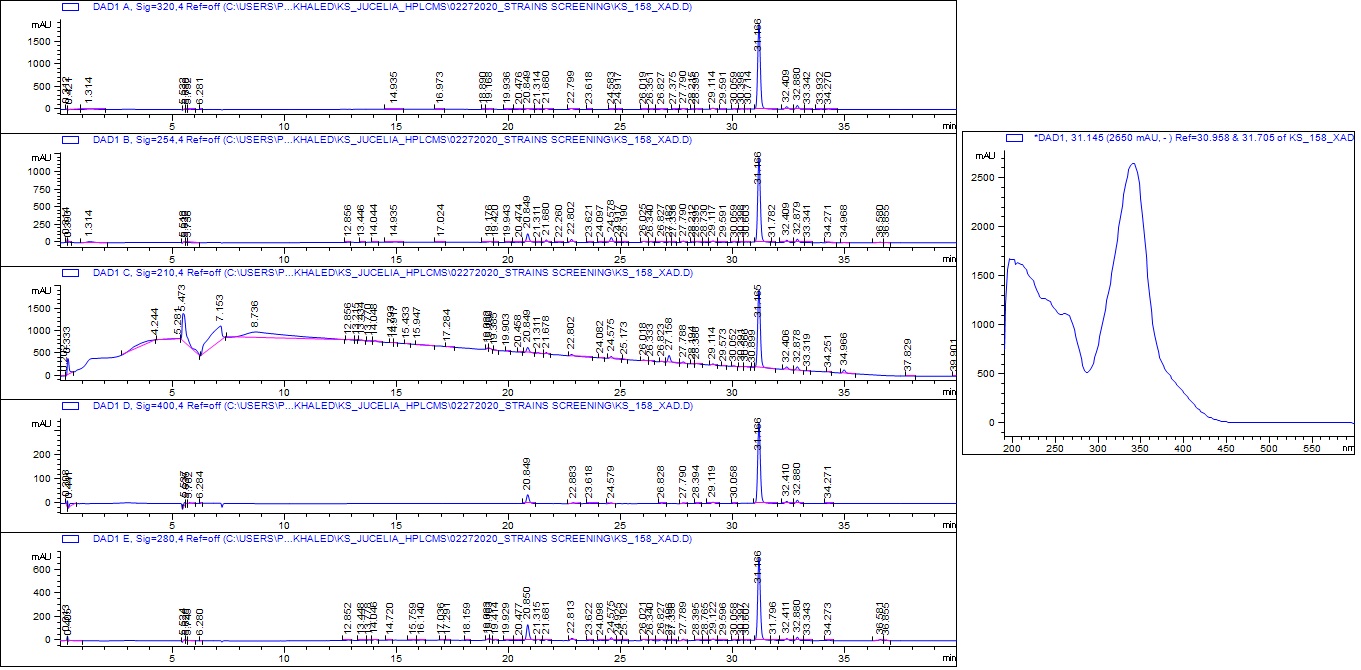


Figure S38. HPLC/UV analyses of the extract produced by *Diaporthe vochysiae* CMRP4332. HPLC-conditions: solvent A: H_2_O/0.1% FA; solvent B: CH_3_CN; flow rate: 0.5 mL min^-1^; 0-30 min, 5-100% B (linear gradient); 30-35 min, 100% B; 35-36 min, 100%-5% B (linear gradient); 36-40 min, 5% B; 320 nm, 254 nm, 210 nm, 400 nm, 280 nm. UV-vis in set of full wavelength scan (190-600 nm).


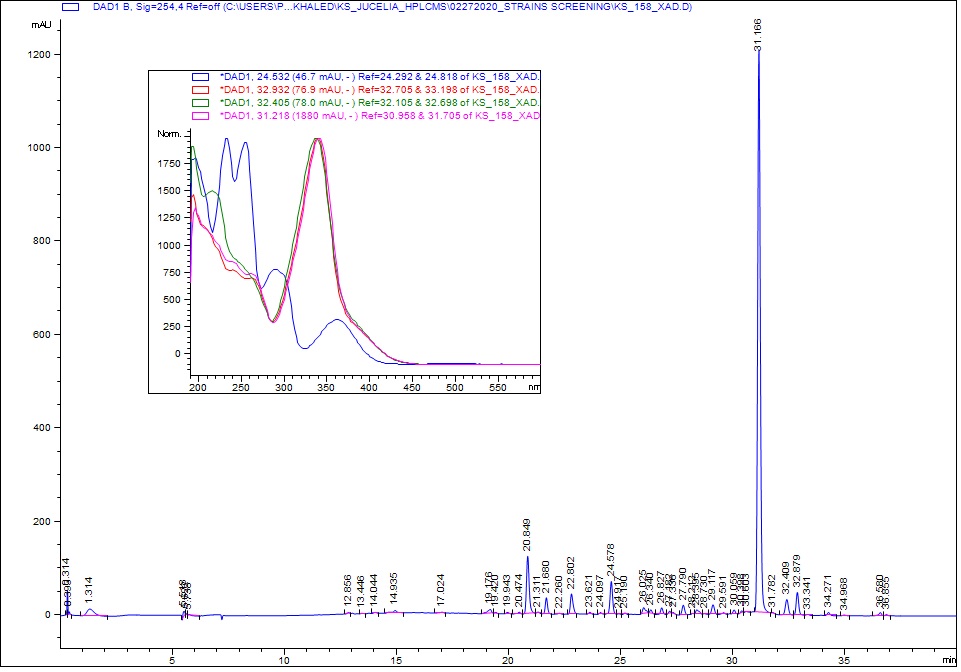


**MW: 414**

**MW: 270**

**MW: 708**

**MW: 708**

Figure S39. HPLC/UVand summary of LCMS analysesof the compounds detected in the extract produced by *Diaporthe vochysiae* CMRP4332. HPLC-conditions: solvent A: H_2_O/0.1% FA; solvent B: CH_3_CN; flow rate: 0.5 mL min^-1^; 0-30 min, 5-100% B (linear gradient); 30-35 min, 100% B; 35-36 min, 100%-5% B (linear gradient); 36-40 min, 5% B; 254 nm. UV-vis inset of full wavelength scan (190-600 nm).

**[M+H]^+^**

**[M+Na]^+^**

**MW: 414**

**MW: 270**

**[M+H]^+^**

Figure S40. (+) and (–)-ESI-MS spectra of the compounds detected in the extract produced by *Diaporthe vochysiae* CMRP4332. LC-MS conditions: H_2_O/0.1% formic acid (solvent A), CH_3_CN/0.1% formic acid (solvent B); flow rate: 0.5 mL min^-1^; 0-2 min, 5% B; 2-30 min, 5-100% B; 30-35 min, 100% B; 35-36 min, 100%-5% B; 36-40 min, 5% B.

**MW: 708**

**[M+H]^+^**

**[M+Na]^+^**

**MW: 708**

**[M+H]^+^**

**[M-H]**^−^

Figure S41.(+) and (–)-ESI-MS spectra of the compounds detected in the extract produced by *Diaporthe vochysiae* CMRP4332. LC-MS conditions: H_2_O/0.1% formic acid (solvent A), CH_3_CN/0.1% formic acid (solvent B); flow rate: 0.5 mL min^-1^; 0-2 min, 5% B; 2-30 min, 5-100% B; 30-35 min, 100% B; 35-36 min, 100%-5% B; 36-40 min, 5% B.


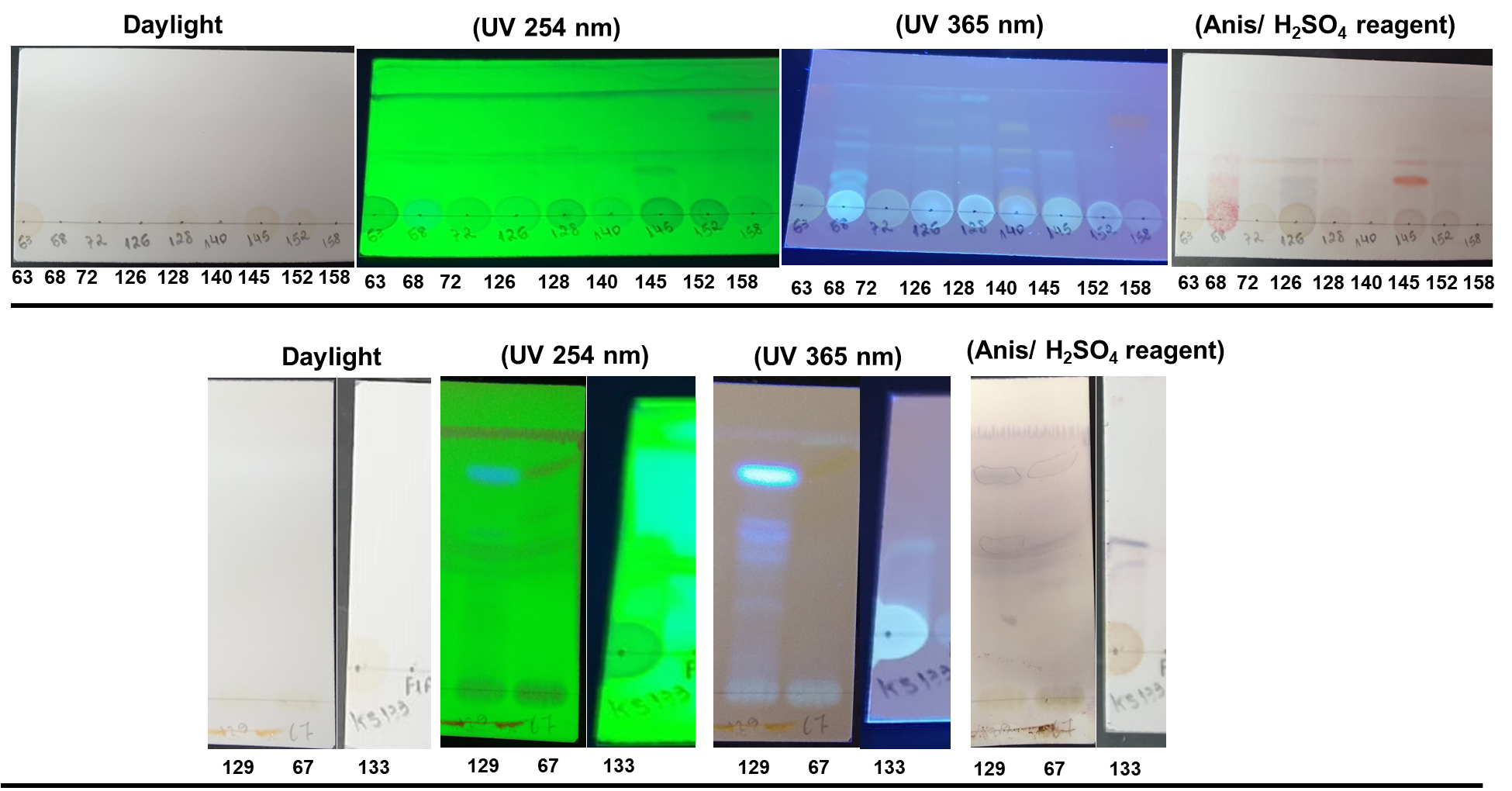


Figure S42. TLC (CH_2_Cl_2_/10%MeOH) screening of the extracts produced by 12 selected endophytic fungi (*Diaporthe vochysiae* CMRP4321 (63), *Diaporthe vochysiae* CMRP4322 (67), *Nemania primolutea* CMRP4323 (68), *Diaporthe cerradensis* CMRP4324 (72), *Coniochaeta* sp. CMRP4325 (126), *Diaporthe vochysiae* CMRP4326 (128), *Aspergillus* sp. section *Flavi* CMRP4327 (129), *Pseudofusicoccum stromaticum* CMRP4328 (133), *Diaporthe* cf. *heveae* 1 CMRP4329 (140), *Diaporthe* sp. CMRP4330 (145), *Diaporthe cerradensis*  CMRP4331 (152), *Diaporthe vochysiae* CMRP4332 (158)).
